# Timing of neonatal mortality and severe morbidity during the postnatal period: a systematic review

**DOI:** 10.11124/JBIES-21-00479

**Published:** 2022-10-24

**Authors:** Justine Dol, Brianna Hughes, Mercedes Bonet, Rachel Dorey, Jon Dorling, Amy Grant, Etienne V. Langlois, Joelle Monaghan, Rachel Ollivier, Robin Parker, Nathalie Roos, Heather Scott, Hwayeon Danielle Shin, Janet Curran

**Affiliations:** 1 Faculty of Health, Dalhousie University, Halifax, NS, Canada; 2 Aligning Health Needs and Evidence for Transformative Change (AH-NET-C): A JBI Centre of Excellence, Dalhousie University, Halifax, NS, Canada; 3 School of Nursing, Dalhousie University, Halifax, NS, Canada; 4 UNDP/UNFPA/UNICEF/WHO/World Bank Special Programme of Research, Development and Research Training in Human Reproduction (HRP), Department of Sexual and Reproductive Health and Research, World Health Organization, Geneva, Switzerland; 5 Division of Neonatal Perinatal Medicine, Department of Pediatrics, Faculty of Medicine, Dalhousie University and IWK Health Centre, Halifax, NS, Canada; 6 Maritime SPOR Support Unit, Halifax, NS, Canada; 7 Partnership for Maternal, Newborn and Child Health, World Health Organization, Geneva, Switzerland; 8 Centre for Research in Family Health, IWK Health Centre, Halifax, NS, Canada; 9 W.K. Kellogg Health Sciences Library, Dalhousie Libraries, Dalhousie University, Halifax, NS, Canada; 10 Clinical Epidemiology Division, Department of Medicine, Karolinska Institutet, Stockholm, Sweden; 11 Department of Obstetrics and Gynecology, Faculty of Medicine, Dalhousie University, Halifax, NS, Canada

**Keywords:** infant, neonatal morbidity, neonatal mortality, newborn, postnatal care, postnatal complications

## Abstract

The objective of this review was to determine the timing of overall and cause-specific neonatal mortality and severe morbidity during the postnatal period (1-28 days).

**Introduction:**

Despite significant focus on improving neonatal outcomes, many newborns continue to die or experience adverse health outcomes. While evidence on neonatal mortality and severe morbidity rates and causes are regularly updated, less is known on the specific timing of when they occur in the neonatal period.

**Inclusion criteria:**

This review considered studies that reported on neonatal mortality daily in the first week; weekly in the first month; or day 1, days 2-7, and days 8-28. It also considered studies that reported on timing of severe neonatal morbidity. Studies that reported solely on preterm or high-risk infants were excluded, as these infants require specialized care. Due to the available evidence, mixed samples were included (eg, both preterm and full-term infants), reflecting a neonatal population that may include both low-risk and high-risk infants.

**Methods:**

MEDLINE, Embase, Web of Science, and CINAHL were searched for published studies on December 20, 2019, and updated on May 10, 2021. Critical appraisal was undertaken by 2 independent reviewers using standardized critical appraisal instruments from JBI. Quantitative data were extracted from included studies independently by 2 reviewers using a study-specific data extraction form. All conflicts were resolved through consensus or discussion with a third reviewer. Where possible, quantitative data were pooled in statistical meta-analysis. Where statistical pooling was not possible, findings were reported narratively.

**Results:**

A total of 51 studies from 36 articles reported on relevant outcomes. Of the 48 studies that reported on timing of mortality, there were 6,760,731 live births and 47,551 neonatal deaths with timing known. Of the 34 studies that reported daily deaths in the first week, the highest proportion of deaths occurred on the first day (first 24 hours, 38.8%), followed by day 2 (24-48 hours, 12.3%). Considering weekly mortality within the first month (n = 16 studies), the first week had the highest mortality (71.7%). Based on data from 46 studies, the highest proportion of deaths occurred on day 1 (39.5%), followed closely by days 2-7 (36.8%), with the remainder occurring between days 8 and 28 (23.0%). In terms of causes, birth asphyxia accounted for the highest proportion of deaths on day 1 (68.1%), severe infection between days 2 and 7 (48.1%), and diarrhea between days 8 and 28 (62.7%). Due to heterogeneity, neonatal morbidity data were described narratively. The mean critical appraisal score of all studies was 84% (SD = 16%).

**Conclusion:**

Newborns experience high mortality throughout the entire postnatal period, with the highest mortality rate in the first week, particularly on the first day. Ensuring regular high-quality postnatal visits, particularly within the first week after birth, is paramount to reduce neonatal mortality and severe morbidity.

## Introduction

Although great strides have been made to improve neonatal outcomes, many newborns worldwide still face severe health outcomes within the first 28 days after birth. In 2019, there were an estimated 2.4 million neonatal deaths globally, suggesting that 6700 newborns died each day.^1^ Neonatal death, defined as mortality among live-born infants during the first 28 days of life, can be further categorized as early neonatal deaths (ie, within the first 7 days after birth) and late neonatal deaths (ie, from the 8th to 28th day after birth).^2^ The most common causes of neonatal mortality include sepsis, intrapartum trauma, and prematurity, which constitute nearly 75% of neonatal deaths.^3^ What remains largely unknown is a global perspective on the timing and causes of neonatal mortality within the first 28 days. Earlier work by Sankar *et al.* 4 published in 2016 found that in low- and middle-income countries (LMICs), almost 60% of neonatal deaths occur within the first 3 days of life.

To explore the timing and causes of neonatal mortality globally, this review focuses on neonates who reflect generally healthy newborns. It must be recognized that differences in mortality timing and causes exist based on when a neonate is born. Previous work has established that the risk of mortality is high immediately after birth, particularly when associated with being born too early or too small,^5^,^6^ indicating that there is variation in risk of mortality based on infant gestation at birth. Additionally, given the known risk factors around preterm infants, preterm or small-for-gestational-age infants are often admitted to a neonatal intensive care unit (NICU) to receive specialized care and improve their chances of survival. Therefore, this review focuses on generally healthy newborns (eg, community-based, non-NICU data) in order to shed light on the timing and causes of neonatal mortality from a broader perspective limiting bias, given that preterm infants tend to have higher mortality and morbidity, and present conditions specific to prematurity or low birth weight.

Although neonatal mortality remains an ongoing concern, rates of severe neonatal morbidity have been increasing with a growing concern across high-income countries and LMICs alike.^7^,^8^ Severe neonatal morbidities that may occur in an apparently healthy newborn can include sepsis, acute respiratory infection/pneumonia, or seizures.^9^-^11^ There are long-term repercussions associated with neonatal morbidities, especially related to early developmental outcomes, school performance, and future hospitalization.^10^,^12^ The severity of complications that can occur during the postnatal period as a result of neonatal morbidity warrants further exploration related to postnatal care.

Despite substantial contextual differences between high-income countries and LMICs,^13^ synthesized evidence is needed on the cause and timing of death and severe morbidity among newborns globally within the first 28 days. In line with the Sustainable Development Goals,^14^ there have been improvements to neonatal care through enhanced training for health care providers and coverage for health care interventions for women and children (eg, immunizations, trained health care providers at birth, essential newborn care) in LMICs.^15^,^16^ Still, coverage for essential interventions targeting the postnatal period (eg, support of breastfeeding initiation and maintenance, quality postnatal visits for mothers and newborns) is suggested to be insufficient.^13^,^17^ Furthermore, coverage is threatened by disruptions of essential newborn care services due to the COVID-19 pandemic, which has been linked to increased adverse neonatal outcomes.^18^ Gaining further insight into when and why neonatal mortality and morbidity occur during the postnatal period will inform policy and recommendations for timely postnatal care.

To address this need, an evidence synthesis was necessary to identify the timing and causes of neonatal mortality and severe morbidity to inform the update of global recommendations related to postnatal care of the mother and newborn. In 2022, the World Health Organization (WHO) updated their 2013 recommendations on postnatal care of the mother and newborn.^19^,^20^ Recommendations state that postnatal care should be provided within the first 24 hours after birth at a health facility or following a home birth. This is to be followed by a minimum of 3 postnatal contacts, with one occurring between 48 and 72  hours, one between days 7 and 14, and one at 6  weeks after birth.^19^,^20^ For the postnatal care guideline update, information from this review was used as part of the evidence to ensure the timing recommendations for postnatal contact are aligned with periods when newborns are experiencing the greatest health challenges.

A preliminary search of PROSPERO, MEDLINE, the Cochrane Database of Systematic Reviews, and the *JBI Evidence Synthesis* was conducted, and no current or ongoing systematic reviews on the timing of overall or cause-specific newborn mortality and morbidity in the postnatal period were identified. Existing reviews focused on specific aspects, such as neonatal mortality timing in LMICs^4^ or maternal and perinatal mortality using institutional data in LMICs.^21^ Building on Sankar *et al.*'s^4^ review, which was conducted in 2012 and focused solely on LMICs, the current review includes all countries, with an updated search in 2021. There is variation in neonatal mortality and morbidity risks largely influenced by where a birth occurs (ie, high-, middle-, or low-income country), with the greatest risk known to occur in low-income countries.^22^ Despite substantial contextual differences across high-income countries and LMICs, the high mortality rates and growing morbidity rates for newborns continue to be a global priority, supporting the broad approach of the current review.

The objective of this review was to determine the timing of overall and cause-specific neonatal mortality and severe morbidity.

## Review questions

What is the timing of overall and cause-specific neonatal mortality and severe morbidity in the postnatal period?

In particular:

- When are newborns dying within the first 28 days after birth (overall and cause-specific)?
- When are newborns experiencing severe morbidity within the first 28 days after birth (overall and cause-specific)?

## Inclusion criteria

### Participants

This review considered reports that included newborns (without known risk factors for complications) from birth to 28 days postnatal, consistent with current WHO definitions.^2^ To be included, studies must have stated that they followed infants up to 28 days regardless of where they were born (ie, at home or at hospital). Studies that reported solely on preterm infants (ie, born before 37 weeks’ gestation) and high-risk infants (eg, malformations, small for gestational age, intrauterine growth restriction, multiples) were excluded from this review. While the original protocol aimed to include only healthy, low-risk neonates, due to the available evidence, mixed samples were included (eg, both preterm and full-term infants) and these are noted in the study characteristics table. Studies that reported data solely on preterm or high-risk infants or from NICUs were excluded because these infants require specialized care, and the timing of deaths and causes are known to vary from those of the general population of all newborns.

### Condition

This review sought to locate existing evidence on the timing of overall and cause-specific neonatal mortality and severe neonatal morbidity during the postnatal period. Neonatal death was defined as deaths among live births during the first 28 completed days.^2^ Neonatal morbidity only included severe morbidities identified after birth and before the end of the neonatal period. Causes of mortality and severe morbidity may have originated in the antenatal or intrapartum period but resulted in death or morbidity during the neonatal period. Considering that mixed neonatal populations were included (ie, sample contained both preterm and full-term newborns), the neonatal causes of death included birth asphyxia, congenital anomalies, prematurity, severe infection, diarrhea, and other/not specified. Causes were identified using the International Statistical Classification of Diseases 10^th^ Revision (ICD-10)^11^,^23^ or as reported by study authors.

### Context

This review considered reports that identified neonates born in a health facility or at home. Although the significant burden of newborn mortality occurs in LMICs,^24^ given that the Sustainable Development Goals focus on development for all countries,^25^ no limits were placed on country. Due to the potential impact of the COVID-19 pandemic on neonatal mortality and severe morbidity, studies that reported on data collected solely after January 2020 were excluded.

### Outcomes

The primary outcomes for this review were timing:

- Of neonatal mortality: overall
- Of neonatal mortality: cause-specific
- And type of severe neonatal morbidity.

Similar to the review by Sankar *et al.*,^4^ timing of neonatal mortality was considered at different time points. Estimates were calculated:

- Daily within the first week after birth (day 1 through day 7)
- By week within the first month (week 1 through week 4)
- By first day (day 1), days 2–7, and days 8–28 (this is an expansion from Sankar *et al.* 4).

First-day mortality was defined as death that occurred within 24 hours of birth, which varied across studies (eg, sometimes described as “first day of life” or “within 24 hours”). Early mortality was typically defined as death that occurred between days 2 and 7. Given the high rate of mortality on the first day and the potential relation to antenatal and intrapartum causes,^4^,^26^,^27^ early mortality was separately analyzed at 2 time points: first day (day 1) and days 2 and 7. Late mortality was defined as death that occurred between day 8 and up to 28 days after birth.^28^

### Types of studies

This review considered reports that provided prevalence or incidence rates for neonatal mortality and severe morbidity outcomes. This included, but was not limited to, population studies, facility-based studies, empirical studies (non-experimental), and/or civil registration vital statistics and population-based records as available through accessing ministry of health websites of the 193 WHO Member States^29^ and WHO Mortality Database. Only quantitative studies reporting on prevalence or incidence data were included, excluding qualitative studies and modeling or estimate data (eg, Bayesian modeling, country-level estimates of mortality or morbidity). Relevant systematic reviews were searched to identify any additional original articles not previously captured in the search. Reports that did not define timing based on any of the above outcomes were excluded, such as Demographic and Health Survey data and Child Health Epidemiology Reference Group, which only reported early (days 1 to 7) and late (days 8 to 28) mortality.

## Methods

This systematic review was conducted in accordance with JBI methodology for systematic reviews of prevalence and incidence.^30^ An advisory panel with clinical expertise in the areas of neonatology and obstetrics was established to provide external consultation and guidance to the team throughout all stages of the review. This review was conducted in accordance with an a priori peer-reviewed, published protocol.^31^ Of note, the protocol included both maternal and neonatal outcomes. The maternal outcomes findings are reported separately.^32^ The methods section describes the approach used for this review, noting any deviations from the protocol. Due to lack of reporting in the included studies, the originally defined secondary outcomes in the protocol^31^ (timing of rehospitalization/readmission by cause and unscheduled use of health services) were not included in this review.

### Search strategy

The search strategy aimed to locate both published and unpublished reports. An initial limited search of MEDLINE (Ovid) and CINAHL (EBSCOhost) was undertaken to identify articles on the topic. The text words contained in the titles and abstracts of relevant articles, and the index terms used to describe the articles were used by an experienced information specialist (RP) to develop a full search strategy for MEDLINE through Ovid (see Appendix I). The search strategy, including all identified keywords and index terms, was adapted for each included information source and peer-reviewed by a second information specialist.^33^ No language limitations were applied to the searches. In order to capture the broad range of complications and conditions in this review, the search strategy included terms for the outcomes (eg, timing). However, to ensure complete capture, we attempted to identify any articles missed by the searches through the following approaches. Select gray literature sources were searched iteratively to fill gaps identified in the included studies, as were sources identified through stakeholder consultation. Gray literature was manually gathered to attain country-level reports on maternal and newborn health-related outcomes in the postnatal period from health ministry websites and the WHO Mortality Database. A Google Scholar search was conducted between July 2 and 6, 2020, and updated June 9–12, 2021, using each of the WHO Member States^29^ and (maternal OR neonatal) AND (mortality OR morbidity) to further identify potential sources. The reference lists of all studies selected for critical appraisal were screened for additional studies.

Studies published in English, French, and Spanish were included. All reports published since 2000 that reported data after 2000 were considered for this review. This cut-off was selected to include recent evidence for updating the 2013 WHO Recommendations on Postnatal Care of the Mother and Newborn.^19^ Additionally, given the introduction of the Millennium Development Goals in 2000, there was a worldwide shift in measurement of mortality and morbidity, resulting in an increase in data quality and quantity after this period.^34^ Studies that reported on data before 2000 were excluded. If data were reported separately by year, data older than 2000 were excluded.

The databases searched included MEDLINE ALL (Ovid), CINAHL with Full Text (EBSCOhost), Web of Science Core Collection (Web of Science), and Embase on December 20, 2019. These were updated by rerunning the searches on May 10, 2021. Searches were limited to publications since January 1, 2000. Sources of unpublished studies and gray literature included health ministry country websites and the WHO Mortality Database. Additional articles were identified through previous systematic reviews on maternal and neonatal mortality and morbidity that used data published from 2003 to 2012.^4^,^35^ Due to the searching function limitations of the publicly available database interface, we were unable to complete the search in LILACS (BIREME/PAHO/WHO website) as stated in the protocol.

### Study selection

**Figure 1:**
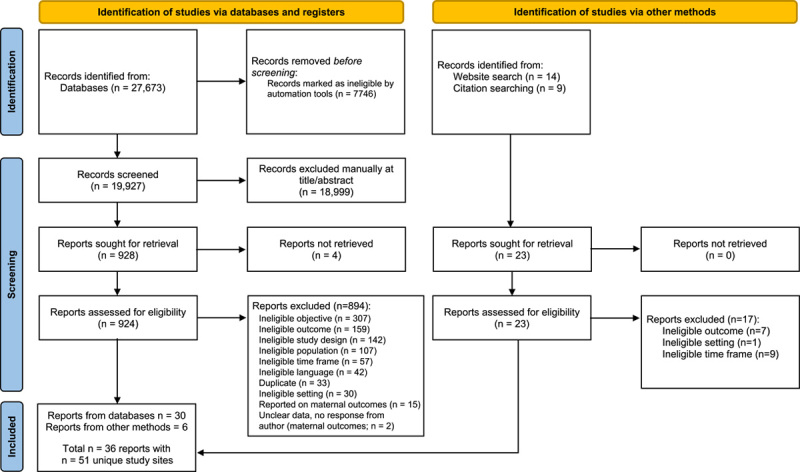
Search results and study selection and inclusion process.^37^

Following the search, all identified citations were collated and uploaded into Covidence (Veritas Health Innovation, Melbourne, Australia)^36^ and duplicates were removed through the Covidence automated duplicate identification tool. Titles and abstracts and full texts were then screened by 2 independent reviewers (JM, RO, RD, HDS, JSD, BH) for assessment against the inclusion criteria for the review. Reasons for exclusion of full-text studies that did not meet the inclusion criteria were recorded (see Appendix II). Any disagreements between reviewers at each stage of the study selection process were resolved with a third reviewer (JSD, BH, JC, MB) or through discussion. The results of the search are presented in a Preferred Reporting Items for Systematic Reviews and Meta-Analyses (PRISMA) flow diagram (Figure 1).^37^

### Assessment of methodological quality

Relevant studies were retrieved in full and their citation details were imported into the JBI System for the Unified Management, Assessment and Review of Information (JBI SUMARI; JBI, Adelaide, Australia).^38^ Eligible studies were critically appraised by 2 independent reviewers (JSD, BR for the studies in English and MB, NR for the studies in French and Spanish) for methodological quality using standardized critical appraisal instruments from JBI, as appropriate.^39^,^40^ Any disagreements that arose were resolved through discussion. The results of the critical appraisal are reported in narrative format and in tables. All studies, regardless of their methodological quality, were included in data extraction and synthesis.

### Data extraction

Data were extracted from papers included in the review by at least 2 independent reviewers (BH, JM, RO, RD, HDS, MB, and NR) using a data extraction tool developed by the reviewers (see Appendix III). Data relevant to the review question and study objectives were extracted, including specific details about populations, study methods, and outcomes of interest (ie, overall timing of mortality, morbidity, and causes). The data extraction tool was modified and revised through piloting before full data extraction. Any disagreements between reviewers were resolved through a third reviewer (JSD) for the English studies and through discussion for the French/Spanish studies. Authors of 8 papers were contacted to request additional data for clarification, with 7 responding with the requested information. For the other paper, we were able to use the data as reported in the Sankar *et al.* 4 study. For 3 of the included studies,^41^-^43^ exact mortality data were not possible to extract from the original figures; thus, the data reported in Sankar *et al.* 4 were used in this review.

### Data synthesis

There were deviations from the protocol regarding data synthesis. First, we estimated missing data if sufficient data points were available, and second, STATA (Stata Corp LLC, Texas, USA) was used instead of RevMan 5.3 (Copenhagen: The Nordic Cochrane Centre, Cochrane). Detailed below are the specific steps taken in the revised data synthesis approach.

To be included in the review, studies must have reported data on a minimum of first-day mortality (day 1), days 2-7, and late mortality (days 8-28). For studies that provided incomplete mortality breakdown during the first week, the study must have provided data for at least 3 time points in the first week after birth to be used in pooling (eg, day 1, day 2, and days 3-7; days 1-3, days 4-7, and days 8-28). Studies that provided full data on the weekly breakdown in the first 28 days (ie, week 1, week 2, week 3, and week 4) were also included, but no pooling or extrapolation was done for weekly data. For pooling of the data, we followed the steps below:

**Step 1:** Studies that had data at each time point were used to obtain the summary estimate (proportion) for each time point.
**Step 2:** This estimate was used to calculate the proportion for the missing time points in other studies (extrapolation). For studies where missing data were estimated for daily deaths, the proportions per day estimated in step 1 were applied to each day with missing data. If data were reported for some of the days (eg, days 1 and 2, but then combined for days 4-6), the proportion for each day estimated in step 1 was applied to the sum of deaths across that group of days. For example, if a study provided data for days 1, 2, 3, and days 4-7, we split the data from days 4-7 into data for day 4, 5, 6, and 7 based on the proportion for each day obtained from actual data in step 1 for these time points. As another example, if a study provided data for days 1-2, and days 3-7, the data were extrapolated for days 1 and 2, based on the proportions obtained in step 1 for a sum of days 1 and 2. Subsequently, the data for days 3, 4, 5, 6, and 7 would be extrapolated from the days 3-7 sum from the pooled proportions for each day obtained from step 1.
**Step 3:** The data were pooled again, using the data from studies that had data for all time points, and the extrapolated data for which estimates of daily deaths were made based on steps 1 and 2 of the pooling processes.
**Step 4:** The pooled results for each day in the first week; each week in the first 4 weeks; and by first day, days 2-7, and days 8-28 are presented as bar graphs with the forest plots in Appendices IV to VI. To determine pooled estimates, analysis took place using the STATA v.14.0 (Stata Corp LLC, Texas, USA) metaprop command for binomial data. Random effects models were run to pool incidence proportion of overall maternal deaths for most analyses. When there were 0 deaths for a specific period (eg, in early mortality), random effects models using the Freeman-Tukey double arcsine transformation to compute the weighted pooled estimate, with a back-transformation on the pooled estimate, were carried out.

The proportional neonatal mortality ratio was defined and calculated as the number of neonatal deaths during a given period over the total number of neonatal deaths known during the postnatal period. For cause-specific analysis, number of deaths due to a specific cause was the numerator and the total number of newborns who died due to that cause in that period was the denominator (eg, number of deaths on day 1 related to infection divided by the total number of newborns who died due to infection).

Subgroup analysis for overall neonatal mortality was conducted based on high-, upper-middle, lower-middle, and low-income countries according to the World Bank.^44^ Although not in the original protocol, an analysis was also conducted to compare studies that reported on data collected in or before 2010 and from 2011 onwards to reflect the changes in neonatal mortality that may have occurred over time. Due to an insufficient number of studies in each category, subgroup analysis on location of birth (facility vs. home) and type of study (population vs. facility-based) was not possible. Where statistical pooling was not possible, the findings are presented in narrative format, including tables and figures to aid in data presentation where appropriate.

Two reports provided data from multiple countries individually,^45^,^46^ so findings are reported separately at the country level, whereas three reports^42^,^47^,^48^ provided data from multiple countries combined, so findings are reported collectively. All other reports provided data from a single country. Hereafter, results of reports with multiple countries are referred to as “studies,” although data in multiple “studies” may have originated from a single published article.

## Results

### Study inclusion

Based on the combined search for maternal and neonatal outcomes, 27,673 records were identified through the original search strategy, and 23 records were identified through other methods (eg, Google Scholar, ministry of health websites). After duplicates were removed, 19,927 records were screened using titles and abstracts, after which 18,999 records were excluded. A total of 924 full-text records were retrieved with 894 excluded, leaving 30 records (see Figure 1).^37^ Of the 23 records identified through website and citation searching, 17 were excluded and 6 included. In total, 36 reports were identified, some of which reported on multiple sites, resulting in a total of 51 unique study sites. Of these 51 studies, 48 reported on neonatal mortality outcomes and 3 reported on severe morbidity outcomes.

### Methodological quality

Articles that met the inclusion criteria were critically appraised for methodological quality as appropriate to their study design (see Tables 1 through 3 for full methodological quality assessment). Six articles were analytical cross-sectional studies, with critical appraisal scores ranging from 57% to 100%.^46^,^49^-^53^ The greatest concerns were whether study subjects and settings were described sufficiently and whether appropriate statistical analysis was used. The most prominent type of article included was cohort study, with 19 studies having critical appraisal scores ranging from 55% to 100%.^15^,^41^-^43^,^45^,^47^,^54^-^66^ For this study type, the greatest concerns were whether confounding factors were identified and dealt with appropriately, as well as whether they used the appropriate statistical analysis. The remaining 11 articles were prevalence studies, with critical appraisal scores ranging from 56% to 100%.^48^,^67^-^76^ For prevalence studies, the greatest concern was whether the study subjects and the setting were described in sufficient detail. The mean critical appraisal score was 84% (SD = 16%) and median was 88%.

**Table 1:** Critical appraisal of analytical cross-sectional studies

Citation	Q1	Q2	Q3	Q4	Q5	Q6	Q7	Q8	%
Batieha *et al.* 2015^49^	Y	Y	Y	Y	Y	Y	Y	Y	100
Bogale *et al.*, 2017^50^	Y	Y	Y	Y	Y	Y	Y	Y	100
Fottrell *et al.*, 2015^46^	U	Y	Y	Y	Y	Y	Y	U	75
Ivanova *et al.*, 2020^51^	Y	N	Y	Y	N	N/A	Y	U	57
Limaso *et al.*, 2020^52^	Y	Y	Y	Y	Y	Y	Y	Y	100
Upadhyay *et al.*, 2013^53^	Y	U	Y	Y	Y	Y	U	U	63
%	83	67	100	100	83	100	83	50	

Y, yes; N, no; U, unclear; N/A, not applicable JBI critical appraisal checklist for analytical cross-sectional studies Q1. Were the criteria for inclusion in the sample clearly defined? Q2. Were the study subjects and the setting described in detail? Q3. Was the exposure measured in a valid and reliable way? Q4. Were objective, standard criteria used for measurement of the condition? Q5. Were confounding factors identified? Q6. Were strategies to deal with confounding factors stated? Q7. Were the outcomes measured in a valid and reliable way? Q8. Was appropriate statistical analysis used?

**Table 2:** Critical appraisal of cohort studies

Citation	Q1	Q2	Q3	Q4	Q5	Q6	Q7	Q8	Q9	Q10	Q11	%
AMANHI, 2018^45^	Y	Y	Y	U	Y	Y	Y	Y	U	U	Y	73
Bapat *et al.*, 2012^54^	Y	Y	Y	Y	Y	Y	Y	Y	Y	Y	Y	100
Baqui *et al.*, 2006^55^	Y	U	Y	Y	U	Y	Y	Y	U	U	U	55
Belizan *et al.*, 2012^42^	Y	Y	Y	Y	U	Y	Y	Y	Y	N/A	U	80
Chowdhury *et al.*, 2010^56^	Y	Y	Y	U	Y	Y	Y	Y	U	N/A	Y	80
Diallo *et al.*, 2011^57^	Y	Y	Y	Y	Y	Y	Y	Y	Y	Y	Y	100
Edmond *et al.*, 2008^58^	Y	Y	Y	Y	U	Y	Y	Y	Y	N/A	Y	90
Jehan *et al.*, 2009^59^	Y	Y	Y	Y	Y	Y	Y	Y	Y	Y	Y	100
Khatun *et al.*, 2012^41^	Y	Y	Y	Y	U	Y	Y	Y	Y	N/A	U	80
Mengesha *et al.*, 2016^60^	Y	Y	Y	Y	Y	Y	Y	Y	Y	Y	Y	100
Mersha *et al.*, 2019^15^	Y	Y	Y	Y	Y	Y	Y	Y	Y	Y	Y	100
Mullany *et al.*, 2009^61^	Y	Y	Y	Y	Y	Y	Y	Y	Y	Y	Y	100
Munjanja, 2007^62^	Y	Y	Y	U	U	Y	Y	Y	U	N/A	U	60
New Zealand MoH, 2012^63^	Y	Y	Y	Y	U	Y	Y	Y	Y	N/A	U	80
Nga *et al.*, 2012^43^	Y	Y	Y	U	U	Y	Y	Y	Y	N/A	U	70
Niswade *et al.*, 2011^64^	Y	Y	Y	Y	Y	Y	Y	Y	Y	N/A	Y	100
Saleem *et al.*, 2014^47^	Y	Y	Y	U	U	Y	Y	Y	Y	N/A	Y	80
Waiswa *et al.*, 2010^66^	Y	Y	Y	U	N	Y	Y	Y	U	Y	Y	73
Welaga *et al.*, 2013^65^	Y	Y	Y	Y	Y	Y	Y	Y	Y	N/A	Y	100
%	100	95	100	68	53	100	100	100	74	78	68	

Y, yes; N, no; U, unclear; N/A, not applicable JBI critical appraisal checklist for cohort studies Q1. Were the two groups similar and recruited from the same population? Q2. Were the exposures measured similarly to assign people to both exposed and unexposed groups? Q3. Was the exposure measured in a valid and reliable way? Q4. Were confounding factors identified? Q5. Were strategies to deal with confounding factors stated? Q6. Were the groups/participants free of the outcome at the start of the study (or at the moment of exposure)? Q7. Were the outcomes measured in a valid and reliable way? Q8. Was the follow-up time reported and sufficient to be long enough for outcomes to occur? Q9. Was follow-up complete, and if not, were the reasons to loss to follow-up described and explored? Q10. Were strategies to address incomplete follow-up utilized? Q11. Was appropriate statistical analysis used?

**Table 3:** Critical appraisal of studies reporting prevalence

Citation	Q1	Q2	Q3	Q4	Q5	Q6	Q7	Q8	Q9	%
Al-Sheyab *et al.*, 2020^67^	N	Y	Y	Y	Y	Y	Y	Y	Y	89
Auger *et al.*, 2015^68^	Y	Y	Y	N	Y	Y	Y	Y	N/A	88
Guinsburg *et al.*, 2021^69^	Y	Y	Y	N	Y	Y	Y	Y	Y	89
Kulkarni *et al.*, 2007^70^	Y	Y	Y	N	U	Y	Y	U	U	56
Leonard *et al.*, 2019^71^	Y	Y	Y	N	Y	Y	Y	U	N/A	75
Parashar *et al.*, 2017^72^	Y	Y	Y	U	Y	Y	Y	U	Y	78
Puri *et al.*, 2021^73^	Y	U	U	N	U	Y	Y	Y	Y	56
Rasaily, 2008^74^	Y	U	U	U	Y	U	Y	Y	Y	56
Saleem *et al.*, 2020^48^	Y	Y	Y	Y	Y	Y	Y	Y	Y	100
WHO, 2021^75^	Y	Y	Y	Y	Y	Y	Y	Y	Y	100
Yaya *et al.*, 2014^76^	Y	Y	N	Y	Y	Y	Y	Y	Y	89
%	91	82	73	36	82	91	100	73	89	

Y, yes; N, no; U, unclear; N/A, not applicable JBI critical appraisal checklist for studies reporting prevalence data Q1. Was the sample frame appropriate to address the target population? Q2. Were study participants sampled in an appropriate way? Q3. Was the sample size adequate? Q4. Were the study subjects and the setting described in detail? Q5. Was the data analysis conducted with sufficient coverage of the identified sample? Q6. Were valid methods used for the identification of the condition? Q7. Was the condition measured in a standard, reliable way for all participants? Q8. Was there appropriate statistical analysis? Q9. Was the response rate adequate, and if not, was the low response rate managed appropriately?

### Characteristics of included studies

Overall, 36 reports were identified that reported on a total of 51 study sites, with 16 studies that collected data in or before 2010, 25 studies that collected data in 2011 or after, and 10 studies that spanned both periods. Most of the studies were population-based (n = 46). In terms of country-level income classification, 11 studies were conducted in low-income countries, 32 from lower-middle-income countries, 5 from upper-middle-income countries, and 3 from high-income countries. When separate data by year were available, only the most recent data were included, which applied to Auger at al.^68^ with only data from 2001–2012 included. Only one study provided data separately on healthy, full-term newborns.^69^ The remaining articles included a mixed neonatal population in their sample (ie, full-term, preterm, and/or multiples). Not all studies reported on each outcome: 46 studies reported data on overall neonatal mortality timing outcomes, 10 studies reported on cause-specific timing outcomes, and 3 studies reported on neonatal morbidity outcomes. Among the studies that reported on mortality, there were 6,760,731 live births and 47,551 neonatal deaths with timing known. Characteristics of studies reporting neonatal mortality are presented in Table 4, and characteristics of studies reporting neonatal morbidity are presented in Table 5.

**Table 4:** Characteristics of neonatal mortality studies

Study/country	Methods	Study population	Live births	Neonatal deaths	Summary of data collection	Results	Neonatal inclusion criteria	Limitations/ comments
Al-Sheyab *et al.*,^67^ Jordan	Prevalence study; August 2019-January 2020	Population based	10,226	144	National neonatal death surveillance system; health center data	Day 1: 36 (25%) Day 2: 28 (19.4%) Day 3: 23 (16.0%) Day 4: 4 (2.8%) Day 5: 10 (6.9%) Day 6: 8 (5.6%) Day 7: 5 (3.5%) Week 1: 114 (79.2%) Week 2: 23 (16.0%) Week 3: 5 (3.5%) Week 4: 2 (1.4%) Day 1: 36 (25%) Day 2-7: 78 (54.2%) Day 8-28: 30 (20.8%)	Any birth, stillbirth, and neonatal death that occurred within the 5 hospitals and with data entered into the surveillance system.	—
AMANHI^45^ Bangladesh, DRC, India, Pakistan, Ghana, Kenya, Tanzania, Zambia	Prospective study; July 2012-February 2016	Population based	Bangladesh: 26,295 India (H): 35,000 India (U): 37,813 Pakistan (M): 27,062 Pakistan (K): 17,189 DRC: 6145 Ghana: 23,640 Kenya: 30,992 Tanzania (I): 8128 Tanzania (P): 18,882 Zambia: 25,082	Bangladesh: 995 India (H): 1287 India (U): 1575 Pakistan (M): 1198 Pakistan (K): 803 DRC: 147 Ghana: 681 Kenya: 359 Tanzania (I): 218 Tanzania (P): 259 Zambia: 354	Verbal autopsy	Day 1 Bangladesh: 420 (42.2%) India (H): 36 (41.6%) India (U): 822 (52.2%) Pakistan (M): 497 (41.5%) Pakistan (K): 337 (42.0%) DRC: 80 (54.4%) Ghana: 308 (45.2%) Kenya: 172 (47.9%) Tanzania (I): 74 (33.9%) Tanzania (P): 131 (50.6%) Zambia: 124 (35.0%) Day 2 Bangladesh: 158 (15.9%) India (H): 140 (10.9%)) India (U): 91 (5.8%) Pakistan (M): 147 (12.3%) Pakistan (K): 86 (10.7%) DRC: 15 (10.2%) Ghana: 44 (6.5%) Kenya: 54 (15.0%) Tanzania (I): 39 (17.9%) Tanzania (P): 39 (15.1%) Zambia: 69 (19.5%) Day 3 Bangladesh: 91 (9.1%) India (H): 107 (8.3%) India (U): 111 (7.0%) Pakistan (M): 112 (9.3%) Pakistan (K): 74 (9.2%) DRC: 12 (8.2%) Ghana: 52 (7.6%) Kenya: 30 (8.4%) Tanzania (I): 39 (17.9%) Tanzania (P): 26 (10.0%) Zambia: 33 (9.3%) Day 4 Bangladesh: 41.0 (4.1%) India (H): 60.9 (4.7%) India (U): 99.8 (6.3%) Pakistan (M): 66.1 (5.5%) Pakistan (K): 46.2 (5.8%) DRC: 6.3 (4.3%) Ghana: 42.5 (13.8%) Kenya: 11.5 (3.2%) Tanzania (I): 2.2 (5.6%) Tanzania (P): 10.3 (3.9%) Zambia: 19.6 (5.5%) Day 5 Bangladesh: 28.9 (2.9%) India (H): 43.1 (3.3%) India (U): 70.5 (3.5%) Pakistan (M): 46.7 (3.9%) Pakistan (K): 32.6 (4.1%) DRC: 4.4 (3.0%) Ghana: 30.0 (9.7%) Kenya: 8.1 (2.3%) Tanzania (I): 8.6 (3.9%) Tanzania (P): 7.3 (2.8%) Zambia: 13.8 (3.9%) Day 6 Bangladesh: 20.9 (2.1%) India (H): 31.2 (2.4%) India (U): 50.9 (3.2%) Pakistan (M): 33.8 (2.8%) Pakistan (K): 23.6 (2.9%) DRC: 3.2 (2.2%) Ghana: 21.7 (7.0%) Kenya: 5.9 (1.6%) Tanzania (I): 6.2 (2.8%) Tanzania (P): 5.3 (2.0%) Zambia: 10.0 (2.8%) Day 7 Bangladesh: 20.0 (2.0%) India (H): 29.8 (2.3%) India (U): 48.8 (3.1%) Pakistan (M): 32.3 (2.7%) Pakistan (K): 22.6 (2.8%) DRC: 3.1 (2.1%) Ghana: 20.8 (6.8%) Kenya: 5.6 (1.6%) Tanzania (I): 5.9 (2.7%) Tanzania (P): 5.1 (1.9%) Zambia: 6 (2.7%) Day 1 Bangladesh: 420 (42.2%) India (H): 536 (41.6%) India (U): 822 (52.2%) Pakistan (M): 497 (41.5%) Pakistan (K): 337 (42.0%) DRC: 80 (54.4%) Ghana: 308 (45.2%) Kenya: 172 (47.9%) Tanzania (I): 74 (33.9%) Tanzania (P): 131 (50.6%) Zambia: 124 (35.0%) Day 2-7 Bangladesh: 360 (36.2%) India (H): 412 (32.0%) India (U): 472 (30.0%) Pakistan (M): 438 (36.6%) Pakistan (K): 285 (35.5%) DRC: 44 (29.9%) Ghana: 211 (31.0%) Kenya: 115 (32.0%) Tanzania (I): 111 (50.9%) Tanzania (P): 93 (35.9%) Zambia: 155 (43.8%) Day 8-28 Bangladesh: 215 (21.6%) India (H): 339 (26.3%) India (U): 281 (17.8%) Pakistan (M): 263 (22.0%) Pakistan (K): 181 (22.5%) DRC: 23 (15.6%) Ghana: 162 (23.8%) Kenya: 72 (20.1%) Tanzania (I): 33 (15.1%) Tanzania (P): 35 (13.5%) Zambia: 75 (21.2%	NR	Data extrapolated for first week analysis
Auger *et al.*,^68^ Canada	Prevalence study; 1981-2012	Population based	NR	2382	Data from birth and death registries	Day 1: 1227 (51.5%) Day 2-7: 569 (23.9%) Day 8-28: 586 (24.6%)	Live-born infants weighing greater than or equal to 500g	Birth location hospital only; data from after 2000 only
Bapat *et al.*,^54^ India	Cohort study; October 2005-September 2007	Population based	11,305	116	Verbal autopsy	Day 1: 40 (34.5%) Day 2: 12 (10.3%) Day 3: 11 (9.5%) Day 4: 8.9 (7.6%) Day 5: 6.3 (5.4%) Day 6: 4.5 (3.9%) Day 7: 4.3 (3.7%) Day 1: 40 (34.5%) Day 2-7: 47 (40.5%) Day 8-28: 29 (25%)	NR	Data extrapolated for first-week analysis
Baqui *et al.*,^55^ India	Cohort study, time not specified	Population based	NR	618	Data collected by data collectors Cause of death assigned by WHO verbal autopsy algorithm	Day 1: 197 (31.9%) Day 2: 48 (7.8%) Day 3: 62 (10.0%) Day 4: 46 (7.4%) Day 5: 25 (4.0%) Day 6: 28 (4.5%) Day 7: 31 (5.0%) Day 1: 197 (31.9%) Day 2-7: 240 (38.8%) Day 8-28: 181 (29.3%)	All live births that resulted in deaths on 0-27 postnatal days	Also reported on cause-specific mortality
Batieha *et al.*,^49^ Jordan	Analytical cross-sectional study; March 2011-April 2012	Facility based	22,330	327	Health center data and verbal autopsy	Day 1: 137 (41.9%) Day 2-7: 129 (39.4%) Day 8-28: 61 (18.7%)	NR	—
Belizan *et al.*,^42^ Argentina, Guatemala, Kenya, Zambia, India, and Pakistan	Cohort study; October 2009- March 2011	Population based	153,728	3882	Collected data from health centers, national data registries, and the Global Network for Women's and Children's Health Research Registry	Day 1: 2019 (52.0%) Day 2: 427 (11.0%) Day 3: 272 (7.0%) Day 4: 194 (5.0%) Day 5: 116 (3.0%) Day 6: 78 (2.0%) Day 7: 78 (2.0%) Week 1: 3184 (82.0%) Week 2: 349 (9.0%) Week 3: 233 (6.0%) Week 4: 116 (3.0%) Day 1: 2019 (52.0%) Day 2-7: 1165 (30.0%) Day 8-28: 698 (18.0%)	All births	Used data from Sankar *et al.*
Bogale *et al.*,^50^ Ethiopia	Analytical cross-sectional study; March 16-24, 2016, previous 18 months	Population based	NR	37	Verbal autopsy; Dabat Health and Demographic Surveillance System	Day 1: 19 (51.4%) Day 2-7: 9 (24.3%) Day 8-28: 9 (24.3%)	All neonatal deaths	Also reported on cause-specific mortality
Chowdhury *et al.*,^56^ Bangladesh	Cohort study; 2003-2004	Population based	11,291	365	Verbal autopsy; Health and Demographic Surveillance System	Day 1: 136 (37.3%) Day 2: 57 (15.6%) Day 3: 56 (15.3%) Day 4: 30 (8.2%) Day 5: 8 (2.2%) Day 6: 6 (1.6%) Day 7: 6 (1.6%) Day 1: 136 (37.3%) Day 2-7: 163 (44.7%) Day 8-28: 66 (18.1%)	All live-born infants who died within first 28 days of life	
Diallo *et al.*,^57^ Burkina Faso	Cohort study; June 2006–May 2007	Population based	864	40	Verbal autopsy; monthly supervisory visits	Day 1: 8 (20%) Day 2-7: 15 (37.5%) Day 8-28: 17 (42.5%)	NR	Study nested within RCT
Edmond *et al.*,^58^ Ghana	Cohort study; January 2003-June 2004	Population based	19,621	590	Verbal autopsy	Day 1: 242 (41.0%) Day 2-7: 195 (33.1%) Day 8-28: 153 (25.9%)	NR	Study nested within RCT Also reported on cause-specific mortality
Fottrell *et al.*,^46^ Bangladesh, Malawi, India, and Nepal	Analytical cross-sectional study; 2001-2011	Population based	Bangladesh: 42,241 India (E): 8819 Nepal (D): 15,299 Malawi: 22,563 India (S): 10,029 Nepal (M): 6735	Bangladesh: 1324 India (E): 518 Nepal (D): 528 Malawi: 730 India (S): 87 Nepal (M): 204	Verbal autopsy; site-specific surveillance systems	Day 1 Bangladesh: 418 (31.6%) India (E): 176 (34.0%) Nepal (D): 196 (37.1%) Malawi: 293 (40.1%) India (S): 30 (34.5%) Nepal (M): 58 (28.4%) Day 2 Bangladesh: 199 (15.0%) India (E): 64 (12.4%) Nepal (D): 66 (12.5%) Malawi: 120 (16.4%) India (S): 9 (10.3%) Nepal (M): 15 (7.4%) Day 3 Bangladesh: 159 (12.0%) India (E): 50 (9.7%) Nepal (D): 49 (9.3%) Malawi: 63 (8.6%) India (S): 5 (5.7%) Nepal (M): 14 (6.9%) Day 4 Bangladesh: 114 (8.6%) India (E): 21 (4.1%) Nepal (D): 51 (9.7%) Malawi: 50 (6.8%) India (S): 3 (3.4%) Nepal (M): 16 (7.8%) Day 5 Bangladesh: 59 (4.5%) India (E): 28 (5.4%) Nepal (D): 23 (4.4%) Malawi: 24 (3.3%) India (S): 2 (2.3%) Nepal (M): 8 (3.9%) Day 6 Bangladesh: 41 (3.1%) India (E): 19 (3.7%) Nepal (D): 13 (2.5%) Malawi: 25 (3.4%) India (S): 4 (4.6%) Nepal (M): 13 (6.4%) Day 7 Bangladesh: 34 (2.6%) India (E): 10 (1.9%) Nepal (D): 13 (2.5%) Malawi: 22 (3.0%) India (S): 2 (2.3%) Nepal (M): 4 (2.0%) Week 1 Bangladesh: 1024 (77.3%) India (E): 368 (71.0%) Nepal (D): 411 (77.8%) Malawi: 597 (81.8%) India (S): 55 (63.2%) Nepal (M): 128 (62.7%) Week 2 Bangladesh: 148 (11.2%) India (E): 74 (14.3%) Nepal (D): 57 (10.7%) Malawi: 77 (10.5%) India (S): 15 (17.2%) Nepal (M): 32 (15.7%) Week 3 Bangladesh: 85 (6.4%) India (E): 47 (9.1%) Nepal (D): 34 (6.4%) Malawi: 28 (3.8%) India (S): 10 (11.5%) Nepal (M): 25 (12.3%) Week 4 Bangladesh: 67 (5.1%) India (E): 29 (5.6%) Nepal (D): 26 (4.9%) Malawi: 28 (3.8%) India (S): 7 (8.0%) Nepal (M): 19 (9.3%) Day 1 Bangladesh: 418 (31.6%) India (E): 176 (34.0%) Nepal (D): 196 (37.1%) Malawi: 293 (40.1%) India (S): 30 (34.5%) Nepal (M): 58 (28.4%) Day 2-7 Bangladesh: 606 (45.8%) India (E): 192 (37.1%) Nepal (D): 215 (40.7%) Malawi: 304 (41.6%) India (S): 25 (28.7%) Nepal (M): 70 (34.3%) Day 8-28 Bangladesh: 300 (22.7%) India (E): 150 (29.0%) Nepal (D): 117 (22.2%) Malawi: 133 (18.2%) India (S): 32 (36.8%) Nepal (M): 76 (37.3%)	All neonatal deaths and stillbirths in study sites	Secondary analysis Also reported on cause-specific mortality
Guinsburg *et al.*,^69^ Brazil	Prevalence study; 2004-2013	Population based	5,285,112	12,589	Data registry, Civil Registry of São Paulo State	Day 1: 3921 (31.1%) Day 2: 1528 (12.1%) Day 3: 994 (7.9%) Day 4: 696 (5.5%) Day 5: 544 (4.3%) Day 6: 379 (3.0%) Day 7: 372 (3.0%) Week 1: 8434 (67.0%) Week 2: 1970 (15.6%) Week 3: 1268 (10.1%) Week 4: 917 (7.2%) Day 1: 3921 (31.1%) Day 2-7: 4513 (35.8%) Day 8-28: 4155 (33.0%)	All infants born, >400g and/or gestational age >22 weeks in São Paulo State to mothers residing in the state in 2004-2013	Data from gestational age 37-41 only
Ivanova *et al.*,^51^ Macedonia	Analytical cross-sectional study; 2011-2017	Facility based	36,733	912	Health center data	Day 1: 335 (36.7%) Day 2-7: 373 (40.9%) Day 8-28: 204 (22.4%)	Neonatal death of live-born neonates at the facility in the period of 0-28 days after delivery, with birth weight more than 500g and full 22 gestational weeks on the day of delivery	—
Jehan *et al.* 59 Pakistan	Cohort study; September 2003-August 2005	Population based	1237	53	Health center data; clinician interview	Day 1: 18.1 (34.2%) Day 2-7: 21 (39.6%) Day 8-28: 14 (26.4%)	NR	Data extrapolated for first week analysis and day 0 and days 1-6
Khatun *et al.*,^41^ Bangladesh	Cohort study; January 2008-December 2009	Population based	NR	260	Verbal autopsy; house supervisory visits	Day 1: 94 (36.2%) Day 2: 31 (12.0%) Day 3: 23 (8.8%) Day 4: 16 (6.2%) Day 5: 8 (3.1%) Day 6: 10 (3.8%) Day 7: 10 (3.8%) Week 1: 192 (73.8%) Week 2: 39 (15%) Week 3: 17 (6.5%) Week 4: 12 (4.6%) Day 1: 94 (36.2%) Day 2-7: 98 (37.7%) Day 8-28: 68 (26.2%)	All deaths among children <5 years of age who were residents of the slums (based on program identification numbers) in the study area	Used data from Sankar *et al.*
Kulkarni *et al.*,^70^ India	Prevalence study; 2003-2005	Population based	NR	63	Health center data; house supervisory visits	Day 1: 31 (49.2%) Day 2-7: 21 (33.3%) Day 8-28: 11 (17.5%)	All perinatal deaths	—
Limaso *et al.*,^52^ Ethiopia	Analytical cross-sectional study January 2018-March 2018	Population based	584	24	Community supervisory	Week 1: 15 (62.5%) Week 2: 5 (20.8%) Week 3: 3 (12.5%) Week 4: 1 (4.2%)	All term pregnancies (≥37 weeks’ gestational age) who live in the study kebeles, neonates followed up for a total of 28 days	—
Mengesha *et al.*,^60^ Ethiopia	Cohort study; April-July 2014	Population based	1152	68	Verbal autopsy; community supervisory	Day 1: 15 (22.1%) Day 2-7: 35 (51.5%) Day 8-28: 18 (26.5%)	Neonates of mothers who gave live birth in the study hospitals or admitted within 6 hours	—
Mersha *et al.*,^15^ Ethiopia	Cohort study; April 2018-March 2019	Facility based	6769	52	Health center data; verbal autopsy	Day 1: 24 (46.2%) Day 2: 9.6 (18.5%) Day 3: 6.7 (12.9%) Day 4: 4.7 (9.0%) Day 5: 2.1 (4.0%) Day 6: 1.5 (2.9%) Day 7: 1.4 (2.7%) Day 1: 24 (46.2%) Day 2-7: 26 (50%) Day 8-28: 2 (3.8%)	Neonates born at the 6 study hospitals who died within 28 days of life	Data extrapolated for first week analysis
Munjanja,^62^ Zimbabwe	Cohort study; January-December 2006	Population based	44,242	506	Verbal autopsy; national data; birth and death registries	Day 1: 250 (49.4%) Day 2: 66 (13.0%) Day 3: 31 (6.1%) Day 4: 13 (2.6%) Day 5: 16 (3.2%) Day 6: 17 (3.4%) Day 7: 25 (4.9%) Day 1: 250 (49.4%) Day 2-7: 168 (33.2%) Day 8-28: 88 (17.4%)	All infants of women recruited to the study	—
New Zealand Ministry of Health,^63^ New Zealand	Cohort study; 2008-2009	Population based	128,618	385	National data	Day 1: 205 (53.2%) Day 2-7: 93 (24.2%) Day 8-28: 87 (22.6%)	All infant with a registered death in 2008-2009	Hospital births only
Nga *et al.*,^43^ Vietnam	Cohort study; 2008-2010	Population based	14,453	233	Verbal autopsy	Day 1: 136 (58.4%) Day 2: 15 (6.4%) Day 3: 14 (6.0%) Day 4: 6 (2.6%) Day 5: 4 (1.7%) Day 6: 6 (2.6%) Day 7: 7 (3.0%) Day 1: 136 (58.4%) Day 2-7: 52 (22.3%) Day 8-28: 45 (19.3%)	NR	Used data from Sankar *et al.* Also reported on cause-specific mortality
Niswade *et al.*,^64^ India	Cohort study; November 2006-October 2007	Population based	1103	36	Health center data; verbal autopsy; surveillance data	Day 1: 15 (41.7%) Day 2-7: 11 (30.6%) Day 8-28: 10 (27.8%)	NR	—
Parashar *et al.*,^72^ India	Prevalence study; July-September 2015	Population based	NR	24	Verbal autopsy	Day 1: 9 (37.5%) Day 2-7: 8 (33.3%) Day 8-28: 7 (29.2%)	All infants who died during the specified period and the parents or family members were available and gave consent	—
Rasaily,^74^ India	Prevalence study; January-July 2003	Population based	29,850	1521	Verbal autopsy; community surveys and supervisory visits	Day 1: 597 (39.3%) Day 2: 111 (7.3%) Day 3: 155 (10.2%) Day 4: 94 (6.2%) Day 5: 84 (5.5%) Day 6: 43 (2.8%) Day 7: 43 (2.8%) Week1: 1127 (74.1%) Week 2: 192 (12.6%) Week 3: 155 (10.2%) Week 4: 47 (3.1%) Day 1: 597 (39.3%) Day 2-7: 530 (34.8%) Day 8-28: 394 (25.9%)	All infants born during reference year	—
Saleem *et al.*, 2014,^47^ Argentina, Guatemala, India, Kenya, Pakistan, Zambia)	Cohort study; 2010-2012	Population based	207,857	5230	Health center data; community supervisory	Day 1: 1804 (34.5%) Day 2: 755 (14.4%) Day 3: 508.2 (9.7%) Day 4: 353.1 (6.8%) Day 5: 249.5 (4.8%) Day 6: 180.5 (3.5%) Day 7: 172.6 (3.3%) Day 1: 1804 (34.5%) Day 2-7: 2219 (42.4%) Day 8-28: 1207 (23.1%)	All infants of women included in study	Data extrapolated for first-week analysis
Saleem *et al.* 2020,^48^ Kenya, Zambia, DRC, Pakistan, India, Guatemala	Prevalence study; January 2010-December 2018	Population based	382,635	4884	Maternal Newborn Health Registry (Global Network for Women's and Children's Health Research)	Day 1: 1787.1 (36.6%) Day 2: 586.9 (12.0%) Day 3: 495.1 (10.1%) Day 4: 343.9 (7.0%) Day 5: 243.1 (5.0%) Day 6: 175.8 (4.0%) Day 7: 168.1 (3.4%) Day 1: 1787.1 (36.6%) Day 2-7: 2013 (41.2%) Day 8-28: 1084 (22.2%)	All live-born infants weighing more or equal to 2500g	Data extrapolated for first week analysis
Upadhyay *et al.*,^53^ India	Analytical cross-sectional study; 2010	Population based	NR	50	Verbal autopsy	Day 1: 22 (44%) Day 2-7: 16 (32%) Day 8-27: 12 (24%)	All infant deaths	—
Waiswa *et al.*,^66^ Uganda	Cohort study; January 2005-December 2008	Population based	NR	64	Verbal autopsy; Health and Demographic Surveillance System	Day 1: 15 (23.4%) Day 2: 15 (23.4%) Day 3: 8 (12.5%) Day 4: 5 (7.8%) Day 5: 6 (9.4%) Day 6: 1 (1.6%) Day 7: 0 (0%) Day 1: 15 (23.4%) Day 2-7: 35 (54.7%) Day 8-28: 14 (21.9%)	Newborns at the time of the study	—
Welaga *et al.*,^65^ Ghana	Cohort study; January 2003-December 2009	Population based	17,751	424	Verbal autopsy	Day 1: 119 (28.1%) Day 2: 55 (13.0%) Day 3: 20 (4.7%) Day 4: 29 (6.8%) Day 5: 16 (3.8%) Day 6: 19 (4.5%) Day 7: 17 (4.0%) Week 1: 275 (64.9%) Week 2: 67 (15.8%) Week 3: 51 (12.0%) Week 4: 31 (7.3%) Day 1: 119 (28.1%) Day 2-7: 156 (36.8%) Day 8-28: 149 (35.1%)	NR	—
World Health Organization,^75^ Macedonia	Prevalence study; January 2019-December 2019	Population based	NR	97	National institutional database systems	Day 1: 31 (32.0%) Day 2-7: 42 (43.3%) Day 8-28: 24 (24.7%)	All stillbirths and neonatal deaths identified during 2019 were entered in a predesigned database	—
Yaya *et al.*,^76^ Ethiopia	Prevalence study; January 2006-December 2010	Population based	11,536	308	Community supervisory	Week 1: 143 (46.4%) Week 2: 72 (23.4%) Week 3: 63 (20.5%) Week 4: 30 (9.7%)	All pregnancies	—

DRC, Democratic Republic of Congo; H, Haryana; I, Ifakara; K, Karachi; M, Matiari; NR, not reported; P, Pemba; RCT, randomized controlled trial; U, Uttar Pradesh

**Table 5:** Characteristics of studies reporting solely on neonatal morbidity

Study/country	Methods	Study population	Live births	Neonatal deaths	Summary of data collection	Morbidity focus	Neonatal inclusion criteria	Limitations/ comments
Leonard *et al.*,^71^ United Kingdom	Prevalence study; January 2010-December 2016	Population based	1,598,069	NR	Health center data	Neonatal group A streptococcus infection	All laboratory-confirmed severe group A streptococcus cases in neonates in London and the Southeast of England with a date of onset within 28 days of birth	—
Mullany *et al.*,^61^ Tanzania	Cohort study (secondary analysis of RCT) September 2004-December 2005	Population based	1,653	NR	Community supervisory visits	Umbilical cord infection	All live-born babies born to women enrolled in a cluster-randomized community-based trial	—
Puri *et al.* 73 Democratic Republic of the Congo, Kenya, and Nigeria	Prevalence study; time period NR	Population based	84,759	237	Community supervisory	Infection, signs of infection	All births	Secondary analysis

NR, not reported

### Review findings

#### Overall neonatal mortality

**Figure 2:**
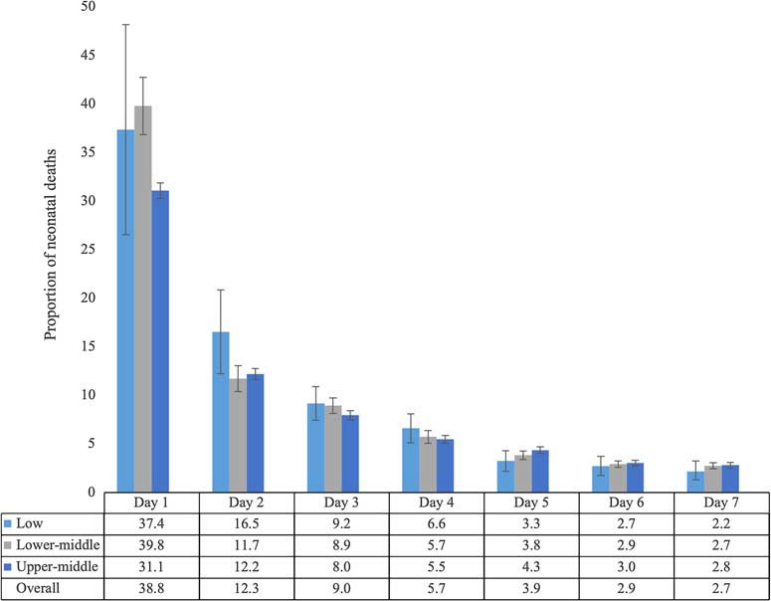
Proportion of neonatal deaths in the first week after birth, overall and by country income level (n = 34 studies).

Based on data from 34 studies, the highest proportion of neonatal deaths within the first week occurred on day 1 (first 24 hours), followed by day 2 (see Figure 2). This is consistent across country income levels. The overall proportion of deaths on each day of the first week was consistent in studies in or before 2010 and in or after 2011. No studies from high-income countries reported on daily deaths within the first week.

**Figure 3:**
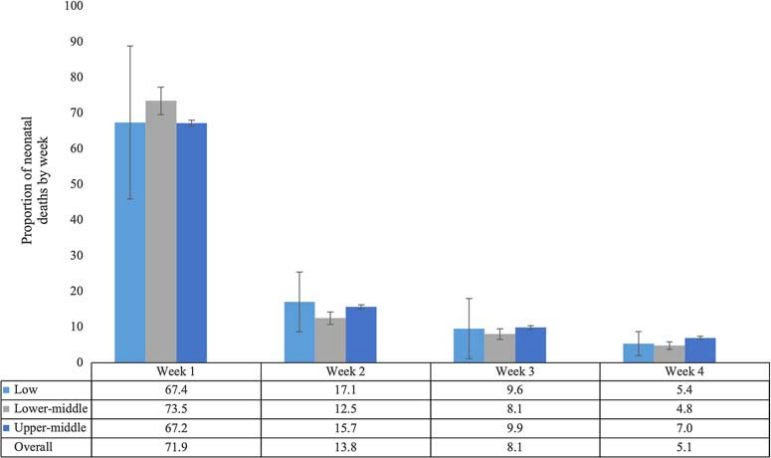
Proportion of neonatal deaths by week, overall and by country income level (n = 16 studies).

Based on data from 16 studies, the highest proportion of deaths was within the first week, followed by the second week (see Figure 3). This was consistent across country income levels, although lower-middle-income countries had a slightly higher proportion of neonatal mortality in the first week, and low-income countries had a higher proportion than other country income levels in the second week. No studies from high-income countries reported on deaths by week. The proportion of deaths by week was similar in studies conducted in or before 2010 and in or after 2011.

**Figure 4:**
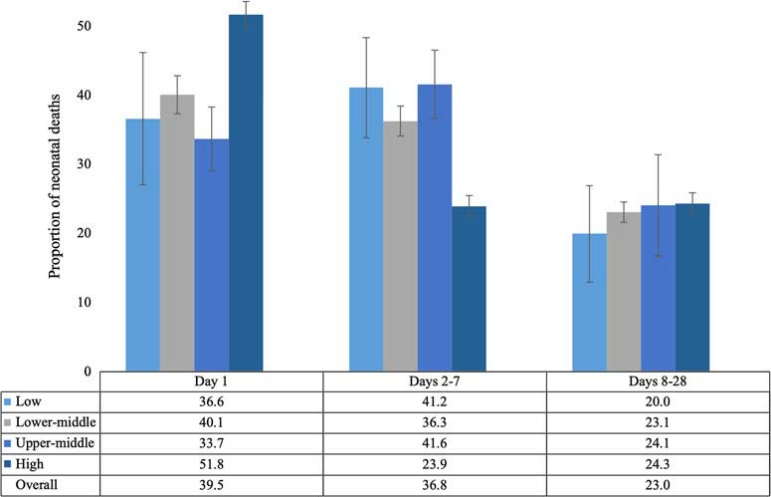
Proportion of neonatal deaths by day 1, days 2-7, and days 8-28, overall and by country income level (n = 46 studies).

Based on data from 46 studies, the highest proportion of deaths occurred on day 1 (39.5%), followed closely by days 2-7 (36.8%, see Figure 4). High-income countries had the highest proportion of mortality on day 1 (51.8%) and the lowest proportion on days 2-7 (23.9%), compared to the other country-level income groups. On the other hand, upper-middle-income countries had the lowest proportion of mortality on day 1 (33.7%) and the highest proportion on days 2-7 (41.6%). The proportion of mortality in the late period (days 8-28) is similar regardless of income classification. The overall proportion of neonatal deaths by day 1, days 2-7, and days 8-28 was similar in studies conducted in or before 2010 and in or after 2011.

#### Cause-specific neonatal mortality

Ten studies reported on cause-specific timing of neonatal mortality.^43^,^46^,^50^,^55^,^58^ Of the total 48,606 neonatal deaths identified, causes were available for 4419 deaths (9.1%). Timing of neonatal mortality was grouped by i) birth asphyxia, ii) congenital anomalies, iii) prematurity, iv) severe infection, v) diarrhea, and vi) other/not specified. As stated previously, these deaths occurred in the neonatal period, but the onset of the cause leading to death may have been during the antenatal/intrapartum period.

**Figure 5:**
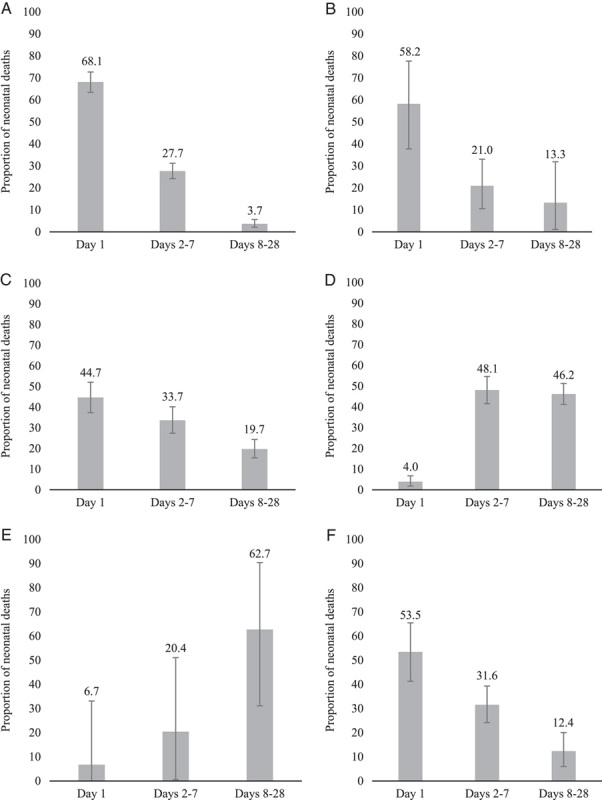
Proportion of neonatal deaths on day 1, days 2-7, and days 8-28 by causes: *(A)* birth asphyxia (n = 10 studies, 1326 deaths); *(B)* congenital anomalies (n = 9 studies, 157 deaths); *(C)* prematurity (n = 10 studies, 968 deaths); *(D)* severe infection (n =10 studies, 1562 deaths); *(E)* diarrhea (n = 4 studies, 17 deaths); *(F)* other/not specified (n = 10 studies, 389 deaths)

**Figure 6:**
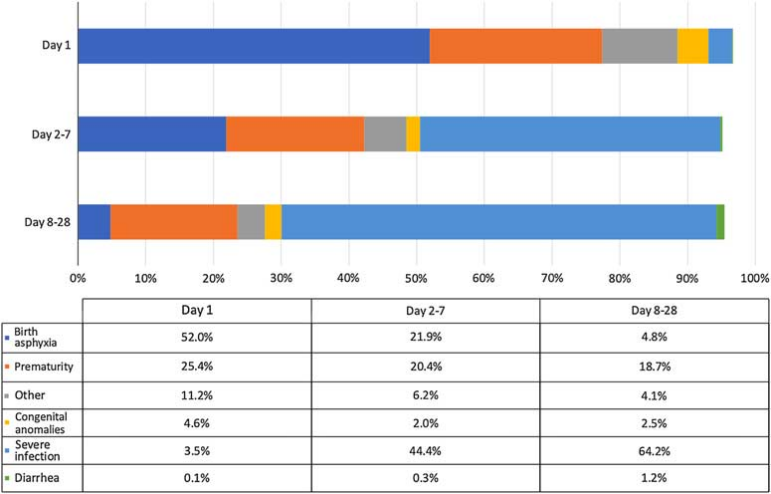
Cumulative proportion of neonatal mortality on day 1, days 2-7, and days 8-28 by causes at each time point.

As seen in Figure 5, neonatal mortality varied widely in the total number of deaths per cause, ranging from 17 deaths from diarrhea to 1562 deaths from severe infection. For the first analysis, we considered the number of deaths by cause and by time over the total number of cause-specific deaths stratified by cause (eg, number of day 1 deaths due to prematurity/total deaths by prematurity). The causes with the highest proportion of deaths on day 1 were birth asphyxia (68.1%) followed by congenital anomalies (58.2%). Between days 2 and 7, causes with the highest proportion were severe infection (48.1%) and prematurity (33.7%). Between days 8 and 28, causes with the highest proportion were diarrhea (62.7%) and severe infection (46.2%). Due to the low number of studies in each outcome, no subgroup analysis was possible. When considering the number of deaths by cause over all cause-specific deaths at each time point (Figure 6), it is clear that birth asphyxia is the most common cause of death on day 1 (52.0%) with severe infection the most common cause of death on days 2-7 (44.4%) and days 8-28 (64.2%).

#### Neonatal morbidity

Three studies report on neonatal morbidity outcomes. Because of the difference in outcomes, this is reported narratively. Leonard *et al.* 71 reported on infants with severe group A streptococcal infections born in London and the Southeast of England between 2010 and 2016. For infants diagnosed with severe group A streptococcal disease, the median onset time was 12 days after delivery (IQR of 7-15 days).^71^ Multiple clinical presentations were noted in 50% of the sample: sepsis (41%), pyrexia, (29%), respiratory distress (12%), infections of the umbilicus or ear (12%), or hypoxic ischemic encephalopathy (6%).^71^

Mullany *et al.* 61 reported on umbilical cord infection in Zanzibar, Tanzania, as part of a secondary analysis of a randomized controlled trial between September 2004 and December 2005. The authors developed 6 sign-based definitions from 4 possible signs of cord infection (pus, redness, swelling, and foul odor), with infection status defined as one or more of the possible signs, with a range of severity (none, mild, moderate, severe).^61^ Among the 1653 infants in the study, the mean onset ranged from 3.0 days (± 2.1 days) for moderate/severe redness to 4.2 days (± 1.8 days) for pus and foul odor alone. Additional signs had a mean onset of 3.2 days (± 1.6) for presence of moderate/severe redness and pus or foul odor, 3.3 days (± 1.8) for moderate/severe redness with pus, 3.5 days (± 1.5) for any redness with pus and foul odor, and 3.7 days (± 1.8) for any redness with pus. Of note, less than 5% of assessments for pus, redness, swelling, and foul odor were positive in the first 48 hours after birth, with 90% of occurring by day 7.^61^

Puri *et al.* 73 reported on serious bacterial infections across 3 countries (Democratic Republic of the Congo, Kenya, and Nigeria) as part of a secondary analysis of the African Neonatal Sepsis Trial. Local infections were identified, including umbilical, skin, eye, and mixed infections, with day 7 (14 per 1000 infants) and day 14 (12 per 1000 infants) being the highest frequency, and day 28 (3 per 1000 infants) and day 1 (4 per 1000 infants) being the lowest frequency. They also reported on signs of systemic infection, with fast breathing being the highest on day 3 (17 per 1000 infants) and day 7 (16 per 1000 infants), and high body temperature being the highest on day 3 (10 per 1000 infants) and day 1 (9 per 1000 infants). All other signs of systemic infections (eg, severe chest indrawing, stopped feeding well, no movement, multiple signs) were less common, fewer than 8 per 1000 at each time point.^73^

## Discussion

This review provides insight into the timing of overall and cause-specific neonatal mortality daily within the first week; weekly in the first month; and comparing day 1, days 2-7, and days 8-28. Across all analyses, the first day after birth (day 1) had the highest number of neonatal deaths. High-income countries had the highest proportion of mortality on day 1 and the lowest proportion on days 2-7, whereas upper-middle-income countries had the lowest proportion of mortality on day 1 and the highest proportion on days 2-7. Similar proportions of mortality on day 1 and days 2-7 were found in lower-middle- and low-income countries. In terms of causes, birth asphyxia, prematurity, and congenital anomalies were the predominant causes of neonatal mortality on day 1, which is expected given their association with intrapartum events. Severe infection is the leading cause of mortality for days 2-7 and days 8-28. Due to heterogeneity, no specific conclusions could be drawn relating to severe neonatal morbidity.

### Overall neonatal mortality

Looking at neonatal mortality, most newborns died on the first day, with over one-third of infants who died in the neonatal period dying on the first day, followed by 12.3% on day 2 and 9.0% on day 3. Looking at early mortality within the first week, 39.9% of infants died on day 1 and 36.4% died between days 2 and 7, which means that more than 75% of infants who died did so in the first week. When looking at country income level differences, high-income countries had a higher proportion of mortality on day 1 (51.8%) and a lower proportion on days 2-7 (23.9%), compared to the other country income level classifications, yet this difference was not apparent between days 8 and 28, which had a relatively low proportion of deaths on average (23.1%). Overall, the findings are in line with Sankar *et al.*'s review^4^ as well as Oza *et al.* 77 who also found that the first day and the first week were the most significant in terms of risk of neonatal mortality. Interestingly, Oza *et al.* 77 found that developed regions (similar to high-income countries in our study) had a lower risk of death on day 1, whereas our review found that high-income countries had a greater proportion on deaths on day 1 versus other country income level groups. This may be related to the differences in data collection, analysis approach, and the limited number of high-income countries included in this review.

It is also interesting to note that there were some variations in terms of timing of neonatal mortality rates within countries as well as between countries. Additionally, due to the breadth of timing of the included studies (2000–2020), there were variations in neonatal mortality rates, although we did not find any significant variation when comparing data collected in or before 2010 or in or after 2011. However, a systematic review exploring the trends on neonatal mortality between 1990 and 2017 found that neonatal mortality rates have been declining since 1990, with the greatest reduction between 2000–2017 compared with 1990–2000.^22^ Therefore, it is important to reflect on differences in findings, which could be related to the inclusion criteria of neonates (eg, community sample containing high-risk and low-risk neonates, low-risk hospital births) or differences in data collection related to follow-up or record-keeping of timing of neonatal mortality. Furthermore, there has been a global increase in the focus on improving neonatal outcomes since the establishment of the Millennium Development Goals in 2000 and the subsequent Sustainable Development Goals in 2015.^14^,^78^

Nevertheless, the first few days and the first week are a critical time for neonatal survival, which makes access to high-quality perinatal health care, education, and counseling essential. Greater emphasis and access to care and support may be required for the whole early neonatal period, beyond just the first day. Early community-based interventions—defined as a multiple-intervention approach offered through a variety of strategies, such as community support groups; women's groups; timely, quality antenatal care; skilled birth attendants; community mobilization; home visitation; and training community health workers—have been found to be effective at reducing neonatal mortality and severe morbidity.^79^ Ensuring access to timely, evidence-based interventions may be an important way to reduce neonatal mortality in the postnatal period.

### Neonatal cause-specific mortality

Looking at the causes of neonatal mortality, most newborns died due to infection after the first day, with deaths due to birth asphyxia, prematurity, and congenital malformations occurring predominantly on day 1. These findings are similar to others showing that the risk of sepsis increased over time in the first week after birth.^4^ Oza *et al.* 80 similarly found that preterm birth and intrapartum complications accounted for most early neonatal deaths (days 1-7), whereas infections caused nearly one-half of neonatal deaths occurring between days 8 and 28. The finding that infection is of greatest risk to newborns who survive the first day is important to note in targeting areas for improved care across the whole neonatal period. Bottlenecks related to neonatal infection include the following: a shortage of health workforce personnel with adequate knowledge, a lack of access to quality antenatal care and assessment, challenges with access to antibiotics, as well as delays in receiving care, either through delayed access due to cultural norms (ie, laying in practices) or challenges being transferred to higher-level facilities.^81^

Additionally, Sankar and colleagues^4^ note that while the proportion of deaths due to birth asphyxia, prematurity, and congenital malformations declined after the first day, there were still a number of deaths that occurred up to the first week, suggesting that ongoing monitoring and regular assessment of newborns by a skilled health care provider is needed in the week after birth. In this review, we excluded studies that reported solely on preterm infants (ie, born before 37 weeks’ gestation) and high-risk infants (eg, malformations, small for gestational age, intrauterine growth restriction, multiples). Almost all of our samples were mixed, generally reflecting population-level estimates, suggesting that even among the community population, it is important to consider birth asphyxia, prematurity, and congenital malformations as key outcomes during the postnatal period, as there is opportunity to improve outcomes for newborns in the postnatal period if adequate, quality care is available.

It is important to acknowledge that causes of neonatal mortality may vary both between and within countries, and only 9% of total neonatal deaths could be attributed to a cause in our analysis. Therefore, our findings related to the timing of cause-specific neonatal mortality must be interpreted with caution. For instance, in an analysis of neonatal mortality trends in India, it was found that not only did the rate of neonatal mortality vary between districts, but the causes of death varied between districts.^82^ Furthermore, because our sample included both high- and low-risk neonates, this may have influenced our findings related to the causes of neonatal mortality, reflecting a mixed-sample population. Future work is needed to further understand the causes of neonatal mortality within the first 28 days to explore both within and between country differences in order to develop appropriate interventions.

### Neonatal morbidity

Studies reporting on severe morbidity were heterogeneous, which did not allow for pooling of data, and thus made it difficult to identify any trends or patterns in the results. It does appear that onset of severe neonatal morbidity occurs early in the neonatal period, with 63% of infants with severe infection diagnosed between day 3^61^ and day 7.^73^ Given the common early onset, it is important that newborns have access to health care providers for early detection and management of infections across the postnatal period. Enhanced discharge counseling and education to ensure families are advised on preventive measures, such as clean cord care, as well as danger signs and symptoms of infection, such as changes in respiratory rates, may be a benefit. However, because the data on morbidity are scarce and heterogeneous, limited conclusions can be drawn about the timing of morbidity. Further work is needed to expand on the knowledge around timing of onset of neonatal morbidity before specific conclusions can be drawn.

### Limitations

While this study has several strengths, there are some limitations that must be acknowledged. First, there are several countries that had data from more than one study from the same geographical area, which may have influenced the incidence rate. However, data were collected from different studies at different time points and with different purposes, and thus it was important to include all studies to provide a broad overview of timing of mortality and morbidity.

Another limitation was the inability to focus on newborns born at low risk of complications, as most studies included a mixed sample that included both preterm and full-term infants and/or multiples. Part of this challenge is that many infants born in LMICs are considered small for gestational age or are born preterm, particularly in countries with high representation in our data (ie, India, Pakistan, and Bangladesh).^83^,^84^ Despite the mixed population in our sample, the findings provide insight into the timing of neonatal mortality and severe morbidity around the world in a community-based sample.

A limitation of the cause-specific mortality data is that causes were not always linked with timing, and only 9.1% of all neonatal deaths reported in the included studies were able to be included in the cause-specific analysis. Studies would often report on the causes of mortality but in reference to the whole neonatal period, without the breakdown by day 1, days 2-7, and days 8-28. Additionally, not all studies reported on causes of death when reporting timing of death. This could partly be due to the challenges in determining causes of death in low-resource areas where autopsies may not be available or feasible.^85^ Furthermore, we reported on the causes of death as reported in studies; however, the number of deaths due to these conditions may only be an estimate, as an infant could have more than one condition, with one cause of death assigned as the primary.

Not all studies reported on each time point, requiring extrapolation, which may not accurately reflect the actual deaths at each time point. Due to limitations in data collection, it is possible that first-day deaths were misclassified as fresh stillbirths and vice versa, as previous studies have acknowledged the challenges of correctly classifying early neonatal mortality in LMICs.^86^,^87^ There is a need for improved data systems for newborns to ensure data accuracy, including neonatal mortality audits/reviews.

Additionally, while there is potential for facility-based factors to influence the neonatal mortality rates, all six facility-based studies followed newborns up to 28 days, similar to all population-based studies. Three of the studies were in countries where most of the births occur in health facilities,^49^,^51^,^67^ suggesting good coverage of births that would occur in the population. However, due to the limited number of studies and how data were reported, we were unable to compare whether birth in a facility or at home impacted neonatal morbidity or mortality. This is an important consideration because newborns born at home may face more complications, and timing of events may not be as clear or well-documented.

It is also important to consider the confounding factor of country income level classification that may influence the level of risk for neonatal mortality when interpreting these findings, such as proximity to health centers, access to primary health care for postnatal assessment, and postnatal advice on neonatal danger signs. Another potential confounding factor is the influence of time on mortality outcomes, as the number of neonatal deaths has been declining over time. However, due to challenges with a lack of data on high-income countries after 2010 and limited country representation of lower-middle-income countries, further analysis was limited.

## Conclusions

This is the first review examining the timing of neonatal mortality and morbidity, both overall and cause-specific, comparing across country income level classification, including high-income countries. Newborns are most likely to die during the first day and the first week, with less than one-fourth of all newborns dying between days 8 and 28. On the first day, the most common causes of death were birth asphyxia, prematurity, and congenital malformations, with infection being the most common cause after day 1. It is important to increase focus on improving access to care throughout the entire postnatal period as an essential way to improve neonatal outcomes and achieve the Sustainable Development Goals of reducing the neonatal mortality rate.^14^

### Recommendations for practice

It is important that newborns continue to receive health care by a skilled health care provider within the first 24 hours after birth, regardless of whether they are born at a health facility or at home (Grade A recommendation^88^). Ready access to quality health care providers who are trained in essential newborn care during this period could potentially decrease the number of deaths that occur on the first day. Early interventions have been found to reduce mortality,^89^ including skin-to-skin contact,^90^,^91^ exclusive breastfeeding,^92^,^93^ and education and training of health care providers.^26^ Furthermore, the provision of quality antenatal care and assessment, as well as having the birth assisted by a skilled attendant, are known to improve both maternal and neonatal outcomes.^94^-^97^ Therefore, given the important burden that mortality and morbidity represent across the first 28 days for newborns, including the late neonatal period, there is a need to ensure continuity in access to and use of postnatal care services, including through effective service delivery models such as midwife-led continuity of care models.^98^

A second recommendation for practice is to continue with regular postnatal follow-up visits within the postnatal period as recommended by the WHO^19^ (Grade A recommendation^88^). Because the incidence of mortality between days 2 and 7 is almost as high as first day mortality, it is important that newborns continue to have access to care during this period. Additionally, given the continued risk of mortality during the late neonatal period, consideration of continuing care during this period is essential.

In addition to the number of postnatal follow-up visits, health care providers should be well-trained, knowledgeable, and have access to life-saving equipment and medication to provide a high quality of care across the high-risk time points.^81^,^99^ In a systematic review and meta-analysis conducted by Langlois and colleagues,^13^ they reported that postnatal care services in LMICs are inequitable and vary depending on socioeconomic status and between urban and rural locations. Thus, investment must be made to strengthen the global health care system with an increased focus on the postnatal period, enhancing not only the quantity of postnatal care follow-up visits, but also the quality in order to deliver high-quality care for newborns, as well as identify newborns left behind.

### Recommendations for research

The reporting period on neonatal mortality should be standardized. For instance, there was a range of definitions of “first day” throughout the studies, from first 24 hours, day 0, and day 1. For future studies and increased ease of meta-analysis, first day mortality should be defined as death that occurs within the first 24 hours. Additionally, reporting early mortality separately from first-day mortality is recommended. Given the high number of deaths that occur on the first day, it is important to tease apart improvements in reducing early neonatal death from the first week and the first day, as some first day mortality cases may be related to antenatal or intrapartum events and outcomes. Another recommendation is to report, in the full study or in a supplementary file, the number of deaths that occur on each day of the neonatal period. This will again allow further insight into when newborns die and when follow-up visits should be scheduled to detect danger signs and provide timely treatment to minimize the number of deaths in the neonatal period.

Further research is also needed on the causes of mortality and severe morbidity linked to timing of death and onset. This area was considerably weaker than the overall timing of mortality data, making recommendations for focused care difficult. Future research is needed to know why newborns are dying in order to provide targeted care and education on those issues.

Additionally, consideration of assessment of place of birth and/or presence of a skilled attendant at birth are recommended. Due to an insufficient number of studies in each category, subgroup analysis on location of birth (facility/home) and type of study (population vs. facility-based) were not possible despite our intention. Therefore, further studies should consider comparing timing of neonatal mortality based on these outcomes as well.

A final recommendation is for more studies on timing of mortality conducted in high-income countries and countries not typically represented in published literature (eg, Middle East, Asia and Pacific). There were few studies that reported on data from countries in these areas, while much work has been conducted in exploring mortality in LMICs. Although data from LMICs help provide a picture of the timing of neonatal mortality and morbidity, many other countries are not represented in this meta-analysis.

## Funding

This project was supported with funding in part by the Canadian Institutes of Health Research (CIHR) under the Strategy for Patient-Oriented Research (SPOR) initiative through the SPOR Evidence Alliance and the UNDP/UNFPA/UNICEF/WHO/World Bank Special Programme of Research, Development and Research Training in Human Reproduction (HRP), Department of Reproductive Health and Research, World Health Organization, Geneva, Switzerland. The funders had no role in study design, data collection and analysis, decision to publish, or preparation of the manuscript.

## Appendix I: Search strategy

### Ovid MEDLINE ALL

Search conducted: December 12, 2019, with 8442 studies identified; updated on May 10, 2021, with 1289 new studies identified after deduplication (using Covidence default duplicate identification)

**Table:**

#	Search
1	((After or following) adj2 (birth^∗^ or deliver^∗^) adj2 (complication^∗^ or morbidit^∗^ or mortalit^∗^ or death^∗^ or hemorrhag^∗^ or haemorrhag^∗^ or bleed^∗^ or anaemi^∗^ or anemi^∗^ or infected or infection^∗^ or sepsis or septic)).ti,ab,kf,kw.
2	((Postnatal^∗^ or post natal^∗^ or post partum or postpartum or puerperal) adj2 (complication^∗^ or morbidit^∗^ or mortalit^∗^ or death^∗^ or hemorrhag^∗^ or haemorrhag^∗^ or bleed^∗^ or anaemi^∗^ or anemi^∗^ or infected or infection^∗^ or septic or sepsis)).ti,ab,kf,kw.
3	((Perinatal or neonat^∗^ or newborn or new born) adj2 (mortalit^∗^ or death^∗^ or infection^∗^ or sepsis or septic or asphyxia or jaundice or fever^∗^ or hypothermi^∗^ or anaemi^∗^ or anemi^∗^)).ti,ab,kf,kw.
4	Perinatal mortality/
5	Perinatal death/
6	Eclampsia/
7	Pre-eclampsia/
8	Postpartum hemorrhage/
9	Maternal death/
10	Maternal mortality/
11	Puerperal Infection/
12	((Maternal or mother^∗^ or pregnan^∗^) adj2 (mortalit^∗^ or death^∗^)).ti,ab,kf,kw.
13	((emergency or unplanned) adj2 (caesarean or cesarean or c-section)).ti,ab,kf,kw.
14	Puerperal Disorders/
15	Asphyxia Neonatorum/
16	exp Anemia, Neonatal/
17	exp Jaundice, Neonatal/
18	Neonatal Sepsis/
19	or/1-18
20	Time factors/
21	(Time or timing).ti,ab,kf,kw.
22	20 or 21
23	19 and 22
24	(incidence or prevalence or epidemiolog^∗^ or cohort^∗^ or survey^∗^ or cross-section^∗^ or population or observational or quantitative).ti,ab,kf,kw.
25	exp epidemiologic methods/
26	exp Epidemiologic Studies/
27	or/24-26
28	23 and 27
29	exp animals/ not humans/
30	(comment or editorial or letter).pt.
31	29 or 30
32	28 not 31
33	limit 32 to yr=“2000 -Current”

Update on 10 May 2021: 33 AND (201912^∗^ or 2020^∗^ or 2021^∗^).dt,ez,ed. = 1589 results

### CINAHL with Fulltext (EBSCOhost)

Search conducted: December 20, 2019, with 3400 studies identified; updated on May 10, 2021, with 217 new studies identified after deduplication (using Covidence default duplicate identification)

**Table:**

S37	S36 Limiters - Published Date: 20000101-
S36	S35 AND NOT S33
S35	S25 AND S34
S34	S28 OR S29 OR S30
S33	S31 AND NOT S32
S32	(MH “Human”)
S31	(MH “Animals+”) OR (MH “Mammals+”)
S30	(MH “Empirical Research”) OR (MH “Case Control Studies+”) OR (MH “Correlational Studies”) OR (MH “Cross Sectional Studies”) OR (MH “Prospective Studies+”) OR (MH “Retrospective Design”) OR (MH “Quasi-Experimental Studies+”) OR (MH “Repeated Measures”)
S29	(MH “Epidemiological Research”) OR (MH “Descriptive Research”) OR (MH “Health Services Research+”) OR (MH “Administrative Research”) OR (MH “Analytic Research”) OR (MH “Applied Research”) OR (MH “Clinical Research”) OR (MH “Survey Research”) OR (MH “Secondary Analysis”) OR (MH “Trend Studies”) OR (MH “Predictive Research”)
S28	S26 OR S27
S27	AB incidence or prevalence or epidemiolog^∗^ or cohort^∗^ or survey^∗^ or cross-section^∗^ or population or observational or quantitative
S26	TI incidence or prevalence or epidemiolog^∗^ or cohort^∗^ or survey^∗^ or cross-section^∗^ or population or observational or quantitative
S25	(S23 AND S24)
S24	(MH “Time Factors”) OR TI (time OR timing) OR AB (time or timing)
S23	S3 OR S6 OR S9 OR S12 OR S15 OR S16 OR S17 OR S18 OR S19 OR S20 OR S21 OR S22
S22	(MH “Jaundice, Neonatal”)
S21	(MH “Anemia, Neonatal”) OR (MH “Neonatal Sepsis”) OR (MH “Asphyxia Neonatorum”)
S20	(MH “Eclampsia”) OR (MH “Pre-Eclampsia”)
S19	(MH “Puerperal Disorders”) OR (MH “Postpartum Hemorrhage”) OR (MH “Puerperal Infection”)
S18	(MH “Infant Death”) OR (MH “Perinatal Death”)
S17	(MH “Infant Mortality”)
S16	(MH “Maternal Mortality”)
S15	S13 OR S14
S14	AB ((emergency or unplanned) N2 (caesarean or cesarean or c-section))
S13	TI ((emergency or unplanned) N2 (caesarean or cesarean or c-section))
S12	S10 OR S11
S11	AB ((Maternal or mother^∗^ or pregnan^∗^) N2 (mortalit^∗^ or death^∗^))
S10	TI ((Maternal or mother^∗^ or pregnan^∗^) N2 (mortalit^∗^ or death^∗^))
S9	S7 OR S8
S8	AB ((Perinatal or neonat^∗^ or newborn or new born) N2 (mortalit^∗^ or death^∗^ or infection^∗^ or sepsis or septic or asphyxia or jaundice or fever^∗^ or hypothermi^∗^ or anaemi^∗^ or anemi^∗^)).
S7	TI ((Perinatal or neonat^∗^ or newborn or new born) N2 (mortalit^∗^ or death^∗^ or infection^∗^ or sepsis or septic or asphyxia or jaundice or fever^∗^ or hypothermi^∗^ or anaemi^∗^ or anemi^∗^)).
S6	S4 OR S5
S5	AB ((Postnatal^∗^ or post natal^∗^ or post partum or postpartum or puerperal) N2 (complication^∗^ or morbidit^∗^ or mortalit^∗^ or death^∗^ or hemorrhag^∗^ or haemorrhag^∗^ or bleed^∗^ or anaemi^∗^ or anemi^∗^ or infected or infection^∗^ or septic or sepsis))
S4	TI ((Postnatal^∗^ or post natal^∗^ or post partum or postpartum or puerperal) N2 (complication^∗^ or morbidit^∗^ or mortalit^∗^ or death^∗^ or hemorrhag^∗^ or haemorrhag^∗^ or bleed^∗^ or anaemi^∗^ or anemi^∗^ or infected or infection^∗^ or septic or sepsis))
S3	S1 OR S2
S2	AB ((After or following) N2 (birth^∗^ or deliver^∗^) N2 (complication^∗^ or morbidit^∗^ or mortalit^∗^ or death^∗^ or hemorrhag^∗^ or haemorrhag^∗^ or bleed^∗^ or anaemi^∗^ or anemi^∗^ or infected or infection^∗^ or sepsis or septic))
S1	TI (((After or following) N2 (birth^∗^ or deliver^∗^)) N2 (complication^∗^ or morbidit^∗^ or mortalit^∗^ or death^∗^ or hemorrhag^∗^ or haemorrhag^∗^ or bleed^∗^ or anaemi^∗^ or anemi^∗^ or infected or infection^∗^ or sepsis or septic))

Update on 10 May 2021: Search as above limited to 20190101 – 20210510 = 677 results

### Web of Science Core Collection

Search conducted: December 20, 2019, with 6151 studies identified; updated on May 10, 2021, with 512 new studies identified after deduplication (using Covidence default duplicate identification)

**Table:**

# 10	#9 AND #8 Indexes=SCI-EXPANDED, SSCI, A&HCI, CPCI-S, CPCI-SSH, ESCI Timespan=2000-2019
# 9	TS=(incidence or prevalence or epidemiolog^∗^ or cohort^∗^ or survey^∗^ or cross-section^∗^ or population or observational or quantitative or longitudinal OR prospective OR retrospective) Indexes=SCI-EXPANDED, SSCI, A&HCI, CPCI-S, CPCI-SSH, ESCI Timespan=2000-2019
# 8	#7 AND #6 Indexes=SCI-EXPANDED, SSCI, A&HCI, CPCI-S, CPCI-SSH, ESCI Timespan=2000-2019
# 7	TS=(time OR timing) Indexes=SCI-EXPANDED, SSCI, A&HCI, CPCI-S, CPCI-SSH, ESCI Timespan=2000-2019
# 6	#5 OR #4 OR #3 OR #2 OR #1 Indexes=SCI-EXPANDED, SSCI, A&HCI, CPCI-S, CPCI-SSH, ESCI Timespan=2000-2019
# 5	TS=((Perinatal or neonat^∗^ or newborn or “new born”) NEAR/2 (mortalit^∗^ or death^∗^ or infection^∗^ or sepsis or septic or asphyxia or jaundice or fever^∗^ or hypothermi^∗^ or anaemi^∗^ or anemi^∗^)) Indexes=SCI-EXPANDED, SSCI, A&HCI, CPCI-S, CPCI-SSH, ESCI Timespan=2000-2019
# 4	TS=((Postnatal^∗^ or “post natal^∗^” or “post partum” or postpartum or puerperal) NEAR/2 (complication^∗^ or morbidit^∗^ or mortalit^∗^ or death^∗^ or hemorrhag^∗^ or haemorrhag^∗^ or bleed^∗^ or anaemi^∗^ or anemi^∗^ or infected or infection^∗^ or septic or sepsis)) Indexes=SCI-EXPANDED, SSCI, A&HCI, CPCI-S, CPCI-SSH, ESCI Timespan=2000-2019
# 3	TS=((emergency or unplanned) NEAR/2 (caesarean or cesarean or “c-section”)) Indexes=SCI-EXPANDED, SSCI, A&HCI, CPCI-S, CPCI-SSH, ESCI Timespan=2000-2019
# 2	TS=((Maternal or mother^∗^ or pregnan^∗^) NEAR/2 (mortalit^∗^ or death^∗^)) Indexes=SCI-EXPANDED, SSCI, A&HCI, CPCI-S, CPCI-SSH, ESCI Timespan=2000-2019
# 1	TS=(((After or following) NEAR/2 (birth^∗^ or deliver^∗^)) NEAR/2 (complication^∗^ or morbidit^∗^ or mortalit^∗^ or death^∗^ or hemorrhag^∗^ or haemorrhag^∗^ or bleed^∗^ or anaemi^∗^ or anemi^∗^ or infected or infection^∗^ or sepsis or septic)) Indexes=SCI-EXPANDED, SSCI, A&HCI, CPCI-S, CPCI-SSH, ESCI Timespan=2000-2019

10 May 2021 update: limited to years 2019-2021 on 20210510 = 1888 results

### Embase

Search conducted: December 23, 2019, with 4555 studies identified; updated on May 10, 2021, with 842 new studies identified after deduplication (using Covidence default duplicate identification).

(((((after OR following) NEAR/2 (birth^∗^ OR deliver^∗^) NEAR/2 (complication^∗^ OR morbidit^∗^ OR mortalit^∗^ OR death^∗^ OR hemorrhag^∗^ OR haemorrhag^∗^ OR bleed^∗^ OR anaemi^∗^ OR anemi^∗^ OR infected OR infection^∗^ OR sepsis OR septic)):ti,ab,kw OR ((postnatal^∗^ OR ‘post natal^∗^’ OR ‘post partum’ OR postpartum OR puerperal) NEAR/2 (complication^∗^ OR morbidit^∗^ OR mortalit^∗^ OR death^∗^ OR hemorrhag^∗^ OR haemorrhag^∗^ OR bleed^∗^ OR anaemi^∗^ OR anemi^∗^ OR infected OR infection^∗^ OR septic OR sepsis)):ti,ab,kw OR ((perinatal OR neonat^∗^ OR newborn OR ‘new born’) NEAR/2 (mortalit^∗^ OR death^∗^ OR infection^∗^ OR sepsis OR septic OR asphyxia OR jaundice OR fever^∗^ OR hypothermi^∗^ OR anaemi^∗^ OR anemi^∗^)):ti,ab,kw OR ((maternal OR mother^∗^ OR pregnan^∗^) NEAR/2 (mortalit^∗^ OR death^∗^)):ti,ab,kw OR ((emergency OR unplanned) NEAR/2 (caesarean OR cesarean OR ‘c-section’)):ti,ab,kw OR (‘perinatal mortality’/exp/mj OR ‘perinatal death’/exp/mj OR ‘newborn morbidity’/exp/mj OR ‘eclampsia and preeclampsia’/exp/mj OR ‘postpartum hemorrhage’/exp/mj OR ‘postpartum anemia’/exp OR ‘maternal death’/exp/mj OR ‘maternal mortality’/exp/mj OR ‘puerperal infection’/exp/mj OR ‘puerperal disorder’/mj OR ‘maternal morbidity’/exp/mj OR ‘newborn hypoxia’/exp/mj OR ‘newborn anemia’/exp/mj OR ‘newborn jaundice’/exp/mj OR ‘newborn sepsis’/exp/mj OR ‘maternal sepsis’/exp)) AND ((time:ti,ab,kw OR timing:ti,ab,kw) OR (‘time factor’/exp OR ‘timing’/exp)) AND ((incidence:ti,ab,kw OR prevalence:ti,ab,kw OR epidemiolog^∗^:ti,ab,kw OR cohort^∗^:ti,ab,kw OR survey^∗^:ti,ab,kw OR ‘cross-section^∗^’:ti,ab,kw OR population:ti,ab,kw OR observational:ti,ab,kw OR quantitative:ti,ab,kw) OR (‘epidemiology’/exp OR ‘epidemiological data’/exp))) NOT ((‘animal’/exp NOT ‘human’/exp) OR (‘letter’/exp OR ‘editorial’/exp OR ‘note’/exp))) AND [embase]/lim NOT ([embase]/lim AND [medline]/lim) AND [2000-2020]/py

10 May 2021 update: Search rerun AND [1-1-2019]/sd NOT [10-5-2021]/sd = 921 results

## Appendix II: Studies ineligible following full-text review

**Table:**

Title	Authors	Year	Journal	Reason for exclusion
Risk factors for readmission for phototherapy due to jaundice in healthy newborns: a retrospective, observational study	Blumovich A et al	2021	Neonatal Intensive Care	Duplicate
A one year review of eclampsia in an Ethiopian tertiary care center (Saint Paul's hospital millennium medical college)	Abdulkadir A	2017	Journal of Perinatal Medicine	Duplicate
Post-cesarean surgical site infections according	Opoien HK et al	2007	Acta Obstet. Gynecol. Scand.	Duplicate
Timing of elective repeat cesarean delivery at term and neonatal outcomes	Tita ATN et al	2009	New England Journal of Medicine	Duplicate
Neonatal outcome following primary elective caesarean section beyond 37 weeks of gestation: a 7-year retrospective analysis of a national registry	Wilmink FA et al	2009	American Journal of Obstetrics and Gynecology	Duplicate
Maternal near-miss and death among women with postpartum haemorrhage: a secondary analysis of the Nigeria Near-miss and Maternal Death Survey	Sotunsa JO et al	2019	BJOG	Duplicate
Early-onset neonatal infections in Australia and New Zealand, 2002-2012	Singh T et al	2019	Archives of Disease in Childhood	Duplicate
Eclampsia: still a dreadful situation	Memon RAD	2012	International Journal of Gynecology and Obstetrics	Duplicate
Infant mortality in three population-based cohorts in Southern Brazil: trends and differentials	Santos IS et al	2008	Cadernos de Saude Publica	Duplicate
The impact of postpartum hemorrhage on hospital length of stay and inpatient mortality: a National Inpatient Sample-based analysis.	Marshall AL et al	2017	American Journal of Obstetrics and Gynecology	Duplicate
The impact of postpartum haemorrhage (PPH) on maternal morbidity	Mackeen A & Khong SY	2012	BJOG	Duplicate
Survey of care environment and mortality in a tertiary neonatal intensive care unit.	Lee Y & Chou Y	2005	Clinical Neonatology	Duplicate
Eclampsia: still a dreadful situation	Memon RAD. et al	2011	Medical Forum Monthly	Duplicate
Severe group A streptococcal infections in mothers and their newborns in London and the South East, 2010-2016: assessment of risk and audit of public health management	Leonard A et al	2018	BJOG	Duplicate
Global, regional, and national levels and causes of maternal mortality during 1990-2013: a systematic analysis for the Global Burden of Disease Study 2013	Kassebaum NJ et al	2014	Lancet	Duplicate
Monitoring maternal and newborn health outcomes in Bauchi state, Nigeria: an evaluation of a standards based quality improvement intervention	Kabo I et al	2015	International Journal of Gynecology and Obstetrics	Duplicate
Trends in severe adverse outcomes following postpartum haemorrhage, 2003-2011	Ford JB et al	2015	BJOG	Duplicate
Severe secondary postpartum hemorrhage: a historical cohort study	Debost-Legrand A et al	2015	International Journal of Gynecology and Obstetrics	Duplicate
A 3-year retrospective review of neonatal morbidity and mortality data at the hospital national guido valadares (HNGV), Dili, Timor-Leste	Bucens IK et al	2012	Journal of Paediatrics and Child Health	Duplicate
Should delivery timing for repeat cesarean be reconsidered based on pregnancy dating criteria?	Brookfield K et al	2016	American Journal of Obstetrics and Gynecology	Duplicate
Trends in perinatal deaths from 2010 to 2013 in the Guatemalan Western Highlands	Garces A et al	2015	Reproductive Health	Duplicate
[Epidemiology of maternal mortality in France, 2010-2012]	Deneux-Tharaux, C & Saucedo M	2017	Gynecologie, Obstetrique, Fertilite & Senologie	Duplicate
Emergency department visits for postpartum complications	Brousseau EC et al	2018	Journal of Women's Health	Duplicate
Risk factors for maternal death and trends in maternal mortality in low- and middle-income countries: a prospective longitudinal cohort analysis	Bauserman, M et al	2015	Reproductive Health	Duplicate
Identification of bacterial pathogens and their antimicrobial susceptibility of culture proven early onset neonatal sepsis	Bystricka A et al	2016	Journal of Maternal-Fetal and Neonatal Medicine	Duplicate
Effect of timing of first postnatal care home visit on neonatal mortality in Bangladesh: a prospective cohort study	Baqui AH et al	2009	BMJ	Duplicate
Causes of neonatal and child mortality in India: a nationally representative mortality survey	Bassani DG et al	2010	Lancet	Duplicate
Population-based rates, timing, and causes of maternal deaths, stillbirths, and neonatal deaths in south Asia and sub-Saharan Africa: a multi-country prospective cohort study	Baqui AH et al	2018	Lancet Global Health	Duplicate
A 5-year review of maternal mortality in FMH	Ambreen A et al	2015	BJOG	Duplicate
Incidence and risk factors of sepsis mortality in labor, delivery and postpartum: a population-based study on 5 million births	Al-Ostad G et al	2015	American Journal of Obstetrics and Gynecology	Duplicate
Incidence of and risk factors for sepsis mortality in labor, delivery, and postpartum	Al-Ostad G et al	2015	Obstetrics and Gynecology	Duplicate
Emergency peripartum hysterectomy: a multicenter study of incidence, indications and outcomes in Southwestern Nigeria	Akintayo A et al	2015	International Journal of Gynecology and Obstetrics	Duplicate
Non-obstetric causes of severe maternal complications: a secondary analysis of the Nigeria Near-miss and Maternal Death Survey	Adeniran AS et al	2019	BJOG	Duplicate
[On perinatal and infant mortality in the Arkhangelsk Region]	Ul'ianovskaia SA et al	2013	Arkhiv Patologii	Ineligible language
[Epidemiological analysis of maternal death in Beijing from 1995 to 2010].	Yang, H et al	2011	Chinese Journal of Preventive Medicine	Ineligible language
[Study on maternal deaths in Beijing, from 1996 to 2010]	Yang, H et al	2011	Zhonghua liu xing bing xue za zhi	Ineligible language
Eclampsia and perinatal outcome: a retrospective study in a teaching hospital	Yaliwal RG et al	2011	Journal of Clinical and Diagnostic Research	Ineligible language
[Clinical features of neonatal enterovirus infection]	Shen X-X et al	2020	Chinese Journal of Contemporary Pediatrics	Ineligible language
[An investigation of severe neonatal hyperbilirubinemia in 13 hospitals of Jiangsu Province, China]	Li Q-Q et al	2020	Chinese Journal of Contemporary Pediatrics	Ineligible language
[Maternal deaths at a public maternity Hospital in Fortaleza: an epidemiological study]	Herculano MMS et al	2012	Revista da Escola de Enfermagem da U S P	Ineligible language
[Hospital-acquired infections after caesarean delivery in selected hospitals in the southern Poland]	Wojkowska-Mach J et al	2008	Ginekologia Polska	Ineligible language
Evaluation of maternal and neonatal complications of HELLP syndrome and its risk factors	Sohrabi N et al	2010	Iranian Journal of Obstetrics, Gynecology and Infertility	Ineligible language
[Maternal mortality due to pre-eclampsia/eclampsia in a state in southern Brazil]	Soares VMN et al	2009	Revista Brasileira de Ginecologia e Obstetricia	Ineligible language
[Early neonatal mortality in the Russian Federation in 2010]	Shchegolev AI et al	2013	Arkhiv Patologii	Ineligible language
[Nosocomial infections in a neonatology department, 1995-2002]	Rudnicki J et al	2003	Ginekologia Polska	Ineligible language
Hospitalizations due to complications of pregnancy and maternal and perinatal outcomes in a cohort of pregnant women in the Brazilian Unified National Health System in Sao Paulo, Brazil	Moura BLA et al	2018	Cad. Saude Publica	Ineligible language
[Trends of maternal mortality ratio during 1996-2010 in China]	Zhou Y-Y et al	2011	Chinese Journal of Preventive Medicine	Ineligible language
[Analysis of maternal deaths in Shanghai from 1996 to 2015]	Qin M	2017	Zhonghua fu chan ke za zhi	Ineligible language
[Neonatal mortality in campania region: analysis of causes of death by current data]	Pugliese A et al	2007	Epidemiologia e Prevenzione	Ineligible language
[The analysis of neonatal deaths based on autopsy protocols of the Department of Forensic Medicine in Bialystok in the years 1955-2009]	Ptaszynska-Sarosiek I et al	2011	Archiwum medycyny sadowej i kryminologii	Ineligible language
[Determinants of neonatal mortality: a case-control study in Fortaleza, Ceara State, Brazil]	Nascimento RM et al	2012	Cadernos de Saude Publica	Ineligible language
[The perinatal mortality in the Omskaya Oblast]	Lopushanskii VG & Kravchenko EN	2008	Problemy sotsial'noi gigieny, zdravookhraneniia i istorii meditsiny	Ineligible language
[Neonatal mortality in the Czech Republic 1998-1999]	Plavka R	2000	Ceska Gynekologie	Ineligible language
Maternal deaths in forensic autopsies	Karayel F et al	2005	Jinekoloji ve Obstetrik Dergisi	Ineligible language
Evaluation of causes and therapeutic methods of controlling of postpartum hemorrhage in two governmental hospital of Mashhad, Iran	Lotfalizadeh M et al	2013	Iranian Journal of Obstetrics, Gynecology and Infertility	Ineligible language
[Characteristics of maternal mortality in the university hospital of Pleven for the period of 1977-2001 years]	Markova S et al	2004	Akusherstvo i ginekologiia	Ineligible language
[Peculiarities of maternal mortality in the University Hospital of Pleven for period 1977-2001]	Markova S et al	2007	Akusherstvo i ginekologiia	Ineligible language
Causes of Death in Neonates and Children in 17-Shahrivar Training Hospital of Rasht	Hashemian H et al	2014	Journal of Guilan University of Medical Sciences	Ineligible language
[Impact of vaginal delivery after a previous cesarean section on perinatal outcomes]	Madi JM et al	2013	Revista brasileira de ginecologia e obstetricia	Ineligible language
Incidence and clinical significance of neonatal nosocomial infections	Christova E et al	2001	Pediatriya	Ineligible language
Analysis of influencing factors for pregnancy induced hypertension retinopathy and its influence on pregnancy outcome of mothers and infants	Huang C-M & Yang J-D	2018	International Eye Science	Ineligible language
[Maternal mortality in Sweden underestimated. Registry study of death in connection with pregnancy, delivery and postpartum]	Grunewald C et al	2008	Lakartidningen	Ineligible language
[Perinatal morbidity and mortality in children born to mothers with gestational hypertension]	Galanti B et al	2000	Acta bio-medica de L'Ateneo parmense	Ineligible language
[Causes of neonatal death in the Xiaogan region of Hubei Province between 2007 and 2010]	Fu H-D et al	2012	Chinese journal of contemporary pediatrics	Ineligible language
The causes of perinatal deaths in Croatia in the year 2005	Dražančić A et al	2007	Gynaecologia et Perinatologia	Ineligible language
[Epidemiological profile of maternal deaths in Rio Grande do Sul, Brazil: 2004-2007]	Carreno I et al	2012	Brazilian Journal of Epidemiology	Ineligible language
Evaluation of infant mortality rate in Sakarya Province in 2008: a cross-sectional study	Demir F et al	2015	Nobel Med.	Ineligible language
[Epidemiological features of maternal deaths occurred in Recife, PE, Brazil (2000-2006)]	Correia RA et al	2011	Revista brasileira de enfermagem	Ineligible language
Incidence of the hypothermia in neonates	Palyzyan P et al	2004	HAYAT	Ineligible language
[Dynamics of perinatal and neonatal mortality rate in the period 1990-2005 in Bulgaria]	Zhekova N et al	2007	Akusherstvo i ginekologiia	Ineligible language
[Clinical characteristics and outcomes of cerebral venous sinus thrombosis during pregnancy and puerperium]	Zhou Q et al	2010	Zhonghua fu chan ke za zhi	Ineligible language
Evaluation of the causes of neonatal jaundice, based on the infant's age at disease onset and age at hospital admission	Boskabadi H et al	2016	Tehran University Medical Journal	Ineligible language
[Severe maternal morbidity in an obstetric ICU in Recife, Northeast of Brasil]	Dr Amorim MMR et al	2008	Revista da Associacao Medica Brasileira	Ineligible language
[Spatial analysis of neonatal mortality in the state of Sao Paulo, 2006-2010]	Almeida MCS et al	2014	Revista paulista de pediatria	Ineligible language
[Epidemiology of postpartum hemorrhages in the Umbrian population in the years 2006-2017]	Abraha I et al	2019	Recenti progressi in medicina	Ineligible language
Determinants of neonatal jaundice among neonates admitted to five referral hospitals in Amhara region, Northern Ethiopia: an unmatched case-control study	Bizuneh AD et al	2020	BMJ Paediatrics Open	Ineligible objective
Risk factors for readmission for phototherapy due to jaundice in healthy newborns: a retrospective, observational study	Blumovich A	2020	BMC Pediatrics	Ineligible objective
Changes in infant and neonatal mortality and associated factors in eight cohorts from three Brazilian cities	Carvalho CA et al	2020	Scientific Reports	Ineligible objective
Maternal mortality in an Iraqi tertiary hospital: lessons from the years of the crisis	Obeid RS et al	2020	International Journal of Women's Health and Reproduction Sciences	Ineligible objective
Pobreza y Mortalidad Materna en Chuquisaca Poverty and maternal mortality in Chuquisaca	De La A &; Murillo G	2009	Cuadernos del Hospital de Clínicas	Ineligible objective
Two year audit of perinatal mortality at Kathmandu Medical College Teaching Hospital	Shrestha M et al	2006	Kathmandu University medical journal (KUMJ)	Ineligible objective
[Omission of causes of maternal death in death certificates in Argentina: nationwide observational study Omissao do registro de causas maternas de morte na Argentina: estudo observacional de alcance nacional]	Abalos E et al	2019	Pan American Journal of Public Health	Ineligible objective
Delivery care utilisation and care-seeking in the neonatal period: a population-based study in Vietnam	Malqvist, M et al	2008	Annals of Tropical Paediatrics	Ineligible objective
Effect of timing of first postnatal care home visit on neonatal mortality in Bangladesh: a observational cohort study	Baqui AH et al	2009	BMJ	Ineligible objective
Root causes for late presentation of severe neonatal hyperbilirubinaemia in Egypt	Iskander I et al	2012	Eastern Mediterranean Health Journal	Ineligible objective
Care seeking for fatal illness episodes in neonates: a population-based study in rural Bangladesh	Chowdhury HR et al	2011	BMC Pediatrics	Ineligible objective
The effects of standardised protocols of obstetric and neonatal care on perinatal and early neonatal mortality at a rural hospital in Tanzania	Kruger C et al	2012	International Health	Ineligible objective
Early onset perinatal infection due to group B streptococcus (GBS) in thessaly greece during 2003-2008	Kalaitzi A et al	2010	Journal of Maternal-Fetal and Neonatal Medicine	Ineligible objective
Early discharge of Alberta mothers post-delivery and the relationship to potentially preventable newborn readmissions	Johnson D et al	2002	Canadian Journal of Public Health	Ineligible objective
Predictive factors of hyperbilirubinemia in newborns at University hospital in northern Iran	Jalali SZ et al	2017	Indian J. Exp. Biol.	Ineligible objective
Duration and magnitude of mortality after pregnancy in rural Bangladesh	Hurt LS et al	2008	International Journal of Epidemiology	Ineligible objective
Clinical characteristics of women captured by extending the definition of severe postpartum haemorrhage with 'refractoriness to treatment': a cohort study	Henriquez DDCA et al	2019	BMC Pregnancy and Childbirth	Ineligible objective
Trends in postpartum haemorrhage	Cameron CA et al	2006	Australian and New Zealand Journal of Public Health	Ineligible objective
Transfers to hospital in planned home birth in four Nordic countries - a prospective cohort study	Blix E et al	2016	Acta obstetricia et gynecologica Scandinavica	Ineligible objective
Maternal and neonatal outcomes after caesarean delivery in the African Surgical Outcomes Study: a 7-day prospective observational cohort study	Bishop D et al	2019	Lancet Glob. Health	Ineligible objective
Trends in all-cause mortality across gestational age in days for children born at term	Wu CS et al	2015	PLoS One	Ineligible objective
Is there a difference in the maternal and neonatal outcomes between patients discharged after 24 h versus 72 h following cesarean section? A prospective randomized observational study on 2998 patients	Bayoumi YA et al	2016	The Journal of Maternal-Fetal & Neonatal Medicine	Ineligible objective
Risk factors for maternal death and trends in maternal mortality in low- and middle-income countries: a prospective longitudinal cohort analysis	Bauserman M et al	2015	Reprod. Health	Ineligible objective
A study of maternal mortality in 8 principal hospitals in Pakistan in 2009	Bano N et al	2011	International journal of gynaecology and obstetrics	Ineligible objective
Obstetric admissions to the intensive care unit: a 10-year review	Valgeirsdottir I et al	2012	Acta Obstetricia et Gynecologica Scandinavica	Ineligible objective
Maternal and neonatal outcome in deliveries complicated by intrapartum fever-does time to delivery matter?	Salman L et al	2017	American Journal of Obstetrics and Gynecology	Ineligible objective
Pattern, causes and outcome of neonatal admissions in a teaching hospital, Multan, Pakistan	Rasheed J et al	2018	Rawal Medical Journal	Ineligible objective
Clinical course and prognosis of hemolytic jaundice in neonates in North East of Iran	Boskabadi H et al	2011	Macedonian Journal of Medical Sciences	Ineligible objective
Epidemiological, clinical and delaying characteristics in the process of attention of maternal death in Lambayeque. 2011 - 2016	Verona-Balcazar M et al	2019	Revista Del Cuerpo Medico Del Hospital Nacional Almanzor Aguinaga Asenjo	Ineligible objective
Travel time from home to hospital and adverse perinatal outcomes in women at term in the Netherlands	Ravelli ACJ et al	2011	BJOG	Ineligible objective
[Analysis of neonatal mortality in the University Hospital La Fe Valencia. Years 1971-2009]	Morcillo Sopena, F et al	2012	Anales de pediatria	Ineligible objective
Vaginal breech delivery at term and neonatal morbidity and mortality - a population-based cohort study in Sweden	Ekéus C et al	2019	Journal of Maternal-Fetal & Neonatal Medicine	Ineligible objective
The effect of timing of cord clamping on neonatal venous hematocrit values and clinical outcome at term: a randomized, controlled trial	Ceriani Cernadas JM et al	2006	Pediatrics	Ineligible objective
Mortality among Guarani Indians in Southeastern and Southern Brazil	Cardoso AM et al	2011	Cad. Saude Publica	Ineligible objective
Maternal morbidity associated with cesarean section	Anaya-Prado R et al	2008	Cir. Cir.	Ineligible objective
An opportunity to reduce morbidity in delayed postpartum hemorrhage: multicentre analysis of tranexamic utilization in the emergency department	Amat C et al	2019	Canadian Journal of Emergency Medicine	Ineligible objective
Surgical site infection after caesarean section in relation to operative time	Alkadhim HK & Albdairi AAH.	2019	Indian Journal of Forensic Medicine and Toxicology	Ineligible objective
Assessment of coagulation profile, fibrinogen, protein c, protein s, antithrombin, and vitamin K levels among sudanese neonates with proven sepsis in Omdurman Maternity Hospital, Sudan	Ahmed A et al	2017	Leukemia Research	Ineligible objective
Assessment of maternal near-miss and quality of care in a hospital-based study in Accra, Ghana	Tuncalp O et al	2013	International Journal of Gynaecology and Obstetrics	Ineligible objective
Maternal near-miss and death among women with postpartum haemorrhage: a secondary analysis of the Nigeria Near-miss and Maternal Death Survey	Sotunsa JO et al	2019	BJOB	Ineligible objective
Integration of maternal postpartum services in maternal and child health services in Kaya health district (Burkina Faso): an intervention time trend analysis	Yugbare Belemsaga D	2017	Tropical Medicine and International Health	Ineligible objective
Self-reported pregnancy-related health problems and self-rated health status in Rwandan women postpartum: a population-based cross-sectional study	Semasaka JPS et al	2016	BMC Pregnancy and Childbirth	Ineligible objective
Mortality related to caesarean section in rural Matebeleland North Province, Zimbabwe.	Rutgers RAK et al	2008	The Central African journal of medicine	Ineligible objective
Time of birth and risk of neonatal death at term: retrospective cohort study	Pasupathy D et al	2010	BMJ	Ineligible objective
Maternal deaths from hypertensive disorders: lessons learnt	Nyfløt Tet al	2018	Acta Obstetricia et Gynecologica Scandinavica	Ineligible objective
Risk of mortality subsequent to umbilical cord infection among newborns of southern Nepal: cord infection and mortality	Mullany LC et al	2009	The Pediatric Infectious Disease Journal	Ineligible objective
Perinatal mortality by gestational week and size at birth in singleton pregnancies at and beyond term: a nationwide population-based cohort study	Morken N-H et al	2014	BMC Pregnancy and Childbirth	Ineligible objective
Second-stage vs first-stage caesarean delivery: comparison of maternal and perinatal outcomes	Asicioglu O et al	2014	Journal of Obstetrics and Gynaecology	Ineligible objective
Rates of intrauterine fetal demise and respiratory morbidity at term: determining optimal timing of delivery	Alimena S et al	2016	Obstetrics and Gynecology	Ineligible objective
Pertussis in the newborn: certainties and uncertainties in 2014	Rocha G et al	2015	Paediatr. Respir. Rev.	Ineligible objective
Maternal mortality secondary to acute respiratory failure in Colombia: a population-based analysis	Rojas-Suarez J et al	2015	Lung	Ineligible objective
Hypothermia in Iranian newborns. Incidence, risk factors and related complications	Zayeri F et al	2005	Saudi Medical Journal	Ineligible objective
Causes of maternal deaths in a tertiary care hospital in Larkana, Pakistan	Soomro S et al	2013	Rawal Medical Journal	Ineligible objective
Determinants and causes of maternal mortality in Iran based on ICD-MM: a systematic review	Zalvand R et al	2019	Reproductive Health	Ineligible objective
[The puerperal infection in a delivery center: occurrence and predisposing factors]	Machado NXdS et al	2005	Revista brasileira de enfermagem	Ineligible objective
Eclampsia in Dar es Salaam, Tanzania -- incidence, outcome, and the role of antenatal care	Urassa DP et al	2006	Acta obstetricia et gynecologica Scandinavica	Ineligible objective
Primary post partum hemorrhage an obstetric catastrophe: a review of 270 cases	Usmani I & Bakhsh FM	2013	Medical Forum Monthly	Ineligible objective
Neonatal bacteremia and early onset sepsis-frequency, spectrum of organisms and correlation between clinical symptoms and laboratory abnormalities	Vakrilova L et al	2013	Journal of Perinatal Medicine	Ineligible objective
Maternal mortality 1991-007. Why mothers die in a third level hospital	Valle L et al	2010	Clinica e Investigacion en Ginecologia y Obstetricia	Ineligible objective
Timing of elective repeat cesarean delivery at term and neonatal outcomes	Tita ATN et al	2009	Obstet. Gynecol. Surv.	Ineligible objective
A comparison of morbidity rates attributable to conditions originating in the perinatal period among newborns discharged from United States hospitals, 1989-90 and 1999-2000	Tomashek KM et al	2006	Paediatr. Perinat. Epidemiol.	Ineligible objective
The prevalence of maternal near miss: a systematic review	Tuncalp O et al	2012	BJOG	Ineligible objective
Pregnancy outcomes of multiple repeated cesarean sections in King Chulalongkorn Memorial Hospital	Wuttikonsammakit P & Sukcharoen N	2006	Journal of the Medical Association of Thailand	Ineligible objective
Maternal and fetal outcome in patients with eclampsia at Murtala Muhammad specialist Hospital Kano, Nigeria	Yakasai IA & Gaya SA	2011	Annals of African Medicine	Ineligible objective
Progress on the maternal mortality ratio reduction in Wuhan, China in 2001-2012	Yang S et al	2014	PLoS One	Ineligible objective
Perinatal outcome in women with severe chronic hypertension during the second half of pregnancy	Vigil-De Gracia P et al	2004	International Journal of Gynaecology and Obstetrics	Ineligible objective
A community based case control study on determinants of perinatal mortality in a tribal population of southern India.	Viswanath K et al	2015	Rural and Remote Health	Ineligible objective
Epidemiological characterization of patients with Neonatal Sepsis in a Hospital of Cali city (Colombia), 2014	Vivas MC et al	2017	Arch. Med.	Ineligible objective
The burden of severe maternal outcomes and indicators of quality of maternal care in Nigerian hospitals: a secondary analysis comparing two large facility-based surveys	Vogel JP et al	2019	BJOG	Ineligible objective
Viral infections: contributions to late fetal death, stillbirth, and infant death	Williams EJ et al	2013	The Journal of Pediatrics	Ineligible objective
Antibiotic treatment of suspected and confirmed neonatal sepsis within 28 days of birth: a retrospective analysis	Wagstaff JS et al	2019	Front. Pharmacol.	Ineligible objective
Clinical study on the factors affecting the post-partum recovery of patients with hypertensive pregnancy disorders at a Chinese hospital	Wei J et al	2017	The journal of obstetrics and gynaecology research	Ineligible objective
The changing profile of infant mortality from bacterial, viral and fungal infection over two decades	Williams EJ et al	2013	Acta Paediatr.	Ineligible objective
Maternal near miss: a cross-sectional study in a tertiary hospital in the state of Goias	Wachholz A et al	2018	International Journal of Gynecology and Obstetrics	Ineligible objective
Uterine rupture: trends over 40 years	Al-Zirqi I et al	2016	BJOG	Ineligible objective
Early imaging and adverse neurodevelopmental outcome in asphyxiated newborns treated with hypothermia	Al Amrani F et al	2017	Pediatric Neurology	Ineligible objective
Maternal and fetal outcome of eclamptic patients in a tertiary hospital	Akhtar R et al	2011	Bangladesh Journal of Obstetrics and Gynecology	Ineligible objective
Public-private differences in short-term neonatal outcomes following birth by prelabour caesarean section at early and full term	Adams N et al	2017	The Australian & New Zealand Journal of Obstetrics & Gynaecology	Ineligible objective
Time trends of neonatal mortality by causes of death in Shenyang, 1997-2014	Wu Q-J et al	2016	Oncotarget	Ineligible objective
Epidemiology of obstetric-related ICU admissions in Maryland: 1999-2008*	Wanderer JP et al	2013	Critical Care Medicine	Ineligible objective
Trends of preeclampsia/eclampsia and maternal and neonatal outcomes among women delivering in addis ababa selected government hospitals, Ethiopia: a retrospective cross-sectional study	Wagnew M et al	2016	The Pan African Medical Journal	Ineligible objective
Increasing neonatal mortality among Palestine refugees in the Gaza trip	van den Berg MM et al	2015	PLoS One	Ineligible objective
Obstetric critical care in south-west Uganda: an 18-month survery of maternal critical care admissions and outcomes	Webster K et al	2012	International Journal of Obstetric Anesthesia	Ineligible objective
Prevalence of concomitant acute bacterial meningitis in neonates with febrile urinary tract infection: a retrospective cross-sectional study	Wallace SS et al	2017	The Journal of Pediatrics	Ineligible objective
The burden of severe maternal outcomes and indicators of quality of maternal care in Nigerian hospitals: a secondary analysis comparing two large facility-based surveys	Vogel JP et al	2019	BJOG	Ineligible objective
[Jaundice and urinary tract infection in neonates: simple coincidence or real consequence?]	Abourazzak S et al	2013	Archives de pediatrie	Ineligible objective
Changes in cause of neonatal death over a decade	Wong A et al	2008	The New Zealand Medical Journal	Ineligible objective
Validating the WHO maternal near miss tool: comparing high- and low-resource settings	Witteveen T et al	2017	BMC Pregnancy and Childbirth	Ineligible objective
Missed opportunities in neonatal deaths in Rwanda: applying the three delays model in a cross-sectional analysis of neonatal death	Wilmot, E et al	2017	Maternal and Child Health Journal	Ineligible objective
Effects of caesarean section on maternal health in low risk nulliparous women: a prospective matched cohort study in Shanghai, China	Wang BS et al	2010	BMC Pregnancy Childbirth	Ineligible objective
Neonatal outcome of singleton term breech deliveries in Norway from 1991 to 2011	Vistad I et al	2015	Acta obstetricia et gynecologica Scandinavica	Ineligible objective
Maternal and neonatal individual risks and benefits associated with caesarean delivery: multicentre prospective study	Villar J et al	2007	BMJ	Ineligible objective
Risk of neonatal mortality according to gestational age after elective repeat cesarean delivery	Vilchez G et al	2016	Archives of Gynecology and Obstetrics	Ineligible objective
Multi-country measurement of maternal morbidity	Van Den Broek N	2015	International Journal of Gynecology and Obstetrics	Ineligible objective
[Inequality regarding maternal mortality in Colombian departments in 2000-2001, 2005-2006 and 2008-2009]	Sandoval-Vargas YG et al	2013	Revista de salud publica	Ineligible objective
Determinant factors of maternal mortality from 2016 to 2017 a case-control study in Banjar regency	Palimbo A et al	2019	Indian Journal of Public Health Research and Development	Ineligible objective
Female mortality in reproductive age in Piaui, Brazil, 2008-2012: causes of deaths and associated factors	Madeiro AP et al	2018	Rev. Epidemiol. Control. Infecc.	Ineligible objective
[Medical audit of neonatal deaths with the "three delay" model in a pediatric hospital in Ouagadougou]	Koueta F et al	2011	Sante	Ineligible objective
[Situation of maternal mortality in Peru, 2000 - 2012]	dl Carpio Ancaya L	2013	Revista peruana de medicina experimental y salud publica	Ineligible objective
Impact of the new guidelines for management of newborns at risk of early sepsis due to Group B streptococcus	Diaz MFG et al	2017	Bol. Pediatr.	Ineligible objective
Severe preeclampsia: characteristics and consequences	Alvarez A et al	2015	Finlay	Ineligible objective
Incidence and risk factors of neonatal hypothermia at referral hospitals in Tehran, Islamic Republic of Iran	Zayeri F et al	2007	Eastern Mediterranean Health Journal	Ineligible objective
Hospital-acquired neonatal infections in developing countries	Zaidi AKM et al	2005	Lancet	Ineligible objective
Time trends and regional differences in maternal mortality in China from 2000 to 2005.	Yanqiu G et al	2009	Bulletin of the World Health Organization	Ineligible objective
Intrapartum interventions that affect maternal and neonatal outcomes for vaginal birth after cesarean section	Wu SW et al		J. Int. Med. Res.	Ineligible objective
Trends in maternal mortality in medical college Jabalpur, India in the last 15 years	Tiwari P et al	2017	Journal of SAFOG	Ineligible objective
Maternal sepsis during pregnancy or the postpartum period requiring intensive care admission	Timezguid N et al	2012	International Journal of Obstetric Anesthesia	Ineligible objective
Are we increasing serious maternal morbidity by postponing termination of pregnancy in severe pre-eclampsia/eclampsia?	Thomas T et al	2005	Journal of Obstetrics and Gynaecology	Ineligible objective
Prevalence of postpartum urinary incontinence: a systematic review	Thom DH & Rortveit G	2010	Acta obstetricia et gynecologica Scandinavica	Ineligible objective
The relationship between the five minute Apgar score, mode of birth and neonatal outcomes	Thavarajah H et al	2018	The Journal of Maternal-Fetal & Neonatal Medicine	Ineligible objective
Assessment and comparison of bacterial load levels determined by quantitative amplifications in blood culture-positive and negative neonatal sepsis	Stranieri I et al	2018	Revista do Instituto de Medicina Tropical de Sao Paulo	Ineligible objective
Improvements in US maternal obstetrical outcomes from 1992 to 2006	Srinivas SK et al	2010	Med. Care	Ineligible objective
Maternal and perinatal mortality and complications associated with caesarean section in low-income and middle-income countries: a systematic review and meta-analysis	Sobhy S et al	2019	Lancet	Ineligible objective
Nature of socioeconomic inequalities in neonatal mortality: population based study	Smith LK et al	2010	BMJ	Ineligible objective
Community based maternal death review: lessons learned from ten districts in Andhra Pradesh, India	Singh S et al	2015	Maternal and Child Health Journal	Ineligible objective
The incidence of deep vein thrombosis in women undergoing cesarean delivery	Sia WW et al	2009	Thromb. Res.	Ineligible objective
Perinatal mortality in eastern Uganda: a community based prospective cohort study	Nankabirwa V et al	2011	PLoS One	Ineligible objective
Retrospective review on obstetric cases of critically ill and dead patients in Dongguan	Shen L-H et al	2015	Cell Biochemistry and Biophysics	Ineligible objective
Postpartum haemorrhage management, risks, and maternal outcomes: findings from the World Health Organization Multicountry Survey on Maternal and Newborn Health	Sheldon WR et al	2014	BJOG	Ineligible objective
Risk factors for postpartum emergency department visits in an urban population	Sheen J-J et al	2019	Maternal and Child Health Journal	Ineligible objective
Infant Outcomes After Elective Early-Term Delivery Compared With Expectant Management.	Salemi JL et al	2016	Obstetrics & Gynecology	Ineligible objective
Hospital transmission of community-acquired methicillin-resistant *Staphylococcus aureus* among postpartum women	Saiman L et al	2003	Clinical infectious diseases	Ineligible objective
Ethnic disparity in maternal and infant mortality and its health-system determinants in Sichuan province, China, 2002-14: an observational study of cross-sectional data	Ren Y et al	2017	Lancet	Ineligible objective
Obstetric patients in a surgical intensive care unit: prognostic factors and outcome	Mjahed K et al	2006	Journal of Obstetrics and Gynaecology	Ineligible objective
Neonatal herpes morbidity and mortality in California, 1995-2003	Morris SR et al	2008	Sexually Transmitted Diseases	Ineligible objective
Emergency peripartum hysterectomy: frequency, indications and maternal outcome	Nisar N et al	2009	Journal of Ayub Medical College	Ineligible objective
Post-cesarean surgical site infections according to CDC standards: rates and risk factors. A prospective cohort study	Opoien HK et al	2007	Acta obstetricia et gynecologica Scandinavica	Ineligible objective
Contemporary trends of reported sepsis among maternal decedents in Texas: a population-based study	Oud L	2015	Infectious Diseases and Therapy	Ineligible objective
Severe maternal morbidity and the mode of delivery	Pallasmaa N et al	2008	Acta obstetricia et gynecologica Scandinavica	Ineligible objective
Eclampsia-scenario in a hospital--a ten years study	Pal A et al	2011	Bangladesh Medical Research Council Bulletin	Ineligible objective
Avoidable maternal mortality in Enugu, Nigeria	Ozumba BC et al	2008	Public Health	Ineligible objective
Associated factors and quality of care received among maternal deaths at a regional hospital in Ghana: maternal death audit review	Owusu-Sarpong A et al	2017	African Journal of Reproductive Health	Ineligible objective
Adverse neonatal and maternal outcome following vacuum-assisted vaginal delivery: does indication matter?	Salman L et al	2017	Archives of Gynecology and Obstetrics	Ineligible objective
Women receiving massive transfusion due to postpartum hemorrhage: a comparison over time between two nationwide cohort studies	Ramler PI et al	2019	Acta obstetricia et gynecologica Scandinavica	Ineligible objective
The role of infection and sepsis in the Brazilian Network for Surveillance of Severe Maternal Morbidity	Pfitscher LC et al	2016	Tropical Medicine & International Health	Ineligible objective
A prospective cause of death classification system for maternal deaths in low and middle-income countries: results from the Global Network Maternal Newborn Health Registry	Pasha O et al	2018	BJOG	Ineligible objective
Timing of prophylactic antibiotic administration in term cesarean section: a randomized clinical trial	Nokiani FA et al	2009	Iranian Journal of Clinical Infectious Diseases	Ineligible objective
Emergency obstetric hysterectomy - a five year review	Verma A et al	2017	JK Science	Ineligible objective
Comparison of in-hospital maternal mortality between hospital systems in Queensland, Australia and Louisiana, United States	Morong JJ et al	2017	The Ochsner Journal	Ineligible objective
Maternal and fetal death on weekends	Moaddab A et al	2019	American Journal of Perinatology	Ineligible objective
Postpartum hemorrhage following vaginal delivery: risk factors and maternal outcomes	Miller CM et al	2017	Journal of Perinatology	Ineligible objective
The impact of obstetric unit closures on maternal and infant pregnancy outcomes	Lorch SA et al	2013	Health Services Research	Ineligible objective
Elective cesarean section or not? Maternal age and risk of adverse outcomes at term: a population-based registry study of low-risk primiparous women	Herstad L et al	2016	BMC Pregnancy & Childbirth	Ineligible objective
Maternal mortality in Brazil from 2001 to 2012: time trends and regional differences	Da Silva BGC et al	2016	Brazilian Journal of Epidemiology	Ineligible objective
Audit on management of eclampsia at Sultan Abdul Halim Hospital	Suan MAM et al	2015	Medical Journal of Malaysia	Ineligible objective
Causes of maternal death in the callao region, Peru. Descriptive study, 2000-2015	Tarqui-Mamani C et al	2019	Revista colombiana de obstetricia y ginecologia	Ineligible objective
An analysis of direct causes of maternal mortality	Rahim R et al	2006	Journal of Postgraduate Medical Institute	Ineligible objective
Infection remains a leading cause of neonatal mortality among infants delivered at a tertiary hospital in Karachi, Pakistan.	Mustufa MA et al	2014	Journal of Infection in Developing Countries	Ineligible objective
Severe maternal morbidity for 2004-2005 in the three Dublin maternity hospitala	Murphy CM et al	2009	European Journal of Obstetrics, Gynecology, and Reproductive Biology	Ineligible objective
Impact of different antiseptics on umbilical cord colonization and cord separation time	Ozdemir H et al	2017	J. Infect. Dev. Ctries.	Ineligible objective
The impact of postpartum hemorrhage on hospital length of stay and inpatient mortality: a nationwide inpatient sample (NIS)-based analysis	Marshall AL et al	2017	Thrombosis Research	Ineligible objective
Stillbirth, newborn and infant mortality: trends and inequalities in four population-based birth cohorts in Pelotas, Brazil, 1982-2015	Menezes AMB et al	2019	International Journal of Epidemiology	Ineligible objective
Deliveries, mothers and newborn infants in Sweden, 1973-2000. Trends in obstetrics as reported to the Swedish Medical Birth Register	Odlind V et al	2003	Acta obstetricia et gynecologica Scandinavica	Ineligible objective
Births should not cause deaths: a retrospective analysis of maternal mortality at a tertiary care hospital in eastern India	Lal R et al	2015	Int. J. Sci. Study	Ineligible objective
Leading causes of maternal mortality at an inner-city Hospital, 1949-2017	Manley C et al	2019	American Journal of Obstetrics and Gynecology	Ineligible objective
Systemic inflammatory response syndrome in home delivered neonates: a prospective observational study	Mathur NB et al	2010	Indian Journal of Pediatrics	Ineligible objective
Perinatal audit using the 3-delays model in western Tanzania	Mbaruku G et al	2009	International Journal of Gynaecology and Obstetrics	Ineligible objective
Postpartum hemorrhage in low risk population	Malabarey O et al	2011	Journal of Perinatal Medicine	Ineligible objective
Trends in maternal mortality by sociodemographic characteristics and cause of death in 27 states and the District of Columbia	MacDorman MF et al	2017	Obstetrics and Gynecology	Ineligible objective
Optimal timing for elective cesarean delivery in a Chinese population	Liu X et al	2016	American Journal of Obstetrics and Gynecology	Ineligible objective
Maternal and newborn outcomes of care from community midwives in Pakistan: a retrospective analysis of routine maternity data	Mubeen K et al	2019	Midwifery	Ineligible objective
Preeclampsia in Jordan: incidence, risk factors, and its associated maternal and neonatal outcomes	Khader YS et al	2018	Journal of Maternal-Fetal & Neonatal Medicine	Ineligible objective
Maternal mortality: a ten year review in a tertiary care setup	Khan B et al	2012	Journal of Ayub Medical College	Ineligible objective
Maternal mortaility in a tertiary care hospital a continuing tregedy	Khanum F et al	2013	Journal of Medical Sciences	Ineligible objective
Preventable maternal mortality: geographic/rural-urban differences and associated factors from the population-based Maternal Mortality Surveillance System in China	Liang J et al	2011	BMC Public Health	Ineligible objective
Trends in pregnancy hospitalizations that included a stroke in the United States from 1994 to 2007 reasons for concern?	Kuklina EV et al	2011	Stroke	Ineligible objective
Maternal near-miss and death incidences - frequencies, causes and the referral chain in Somaliland: a pilot study using the WHO near-miss approach	Kiruja J eta l	2017	Sexual & Reproductive Healthcare	Ineligible objective
Bio-social characteristics as determinants of maternal death: a community based case-control study	Khanna D et al	2019	Indian Journal of Public Health Research and Development	Ineligible objective
Clinical course and complications of HELLP syndrome according to time of onset	Gulec UK et al	2012	Clinical and Experimental Obstetrics & Gynecology	Ineligible objective
Maternal mortality in France: epidemiological study, prevalence and characteristics	Bouvier-Colle M-H	2007	Reanimation	Ineligible objective
Pattern of neonatal sepsis in Dubai hospital	Khan A	2016	Journal of Maternal-Fetal and Neonatal Medicine	Ineligible objective
The role of intrapartum fever in identifying asymptomatic term neonates with early-onset neonatal sepsis	Chen KT et al	2002	Journal of Perinatology	Ineligible objective
Delayed cord clamping during elective cesarean deliveries: results of a pilot safety trial	Chantry CJ et al	2018	Maternal Health, Neonatology and Perinatology	Ineligible objective
Infectious diseases are a larger contributor than obstetric causes to maternal mortality in rural western Kenya	Desai M et al	2012	American Journal of Tropical Medicine and Hygiene	Ineligible objective
Length of postnatal stay in healthy newborns and re-hospitalization following their early discharge	Gupta P et al	2006	Indian Journal of Pediatrics	Ineligible objective
Association of mode of delivery with urinary incontinence and changes in urinary incontinence over the first year postpartum	Chang S-R et al	2014	Obstetrics and Gynecology	Ineligible objective
Emergency peripartum hysterectomies: an Australian audit	Balaba K et al	2015	BJOG	Ineligible objective
Association of maternal age with severe maternal morbidity and mortality in Canada	Aoyama K et al	2019	JAMA Network Open	Ineligible objective
The effectiveness and safety of introducing condom-catheter uterine balloon tamponade for postpartum haemorrhage at secondary level hospitals in Uganda, Egypt and Senegal: a stepped wedge, cluster-randomised trial	Anger HA et al	2019	BJOG	Ineligible objective
The WHO application of ICD-10 to deaths during the perinatal period (ICD-PM): results from pilot database testing in South Africa and United Kingdom	Allanson ER et al	2016	BJOG	Ineligible objective
Maternal outcomes of cesarean deliveries at different gestational ages	Zhou CG et al	2018	American Journal of Obstetrics and Gynecology	Ineligible objective
Presence of obstetric risk factors in a late preterm newborn group compared to full-term newborn	Veiga AJMO et al	2017	European Journal of Pediatrics	Ineligible objective
Maternal and newborn outcomes at a tertiary care hospital in Lusaka, Zambia, 2008-2012	Vwalika B et al	2017	International Journal of Gynaecology and Obstetrics	Ineligible objective
Intrapartum fetal deaths and unexpected neonatal deaths in the Republic of Ireland: 2011 - 2014; a descriptive study	McNamara K et al	2018	BMC Pregnancy and Childbirth	Ineligible objective
Neonatal outcomes in early term birth	Parikh LI et al	2014	American Journal of Obstetrics & Gynecology	Ineligible objective
Moving beyond essential interventions for reduction of maternal mortality (the WHO Multicountry Survey on Maternal and Newborn Health): a cross-sectional study	Souza JP et al	2013	Lancet	Ineligible objective
Mortality after near-miss obstetric complications in Burkina Faso: medical, social and health-care factors	Storeng KT et al	2012	Bulletin of the World Health Organization	Ineligible objective
Magnitude, trends and causes of maternal mortality among reproductive aged women in Kersa health and demographic surveillance system, eastern Ethiopia.	Tesfaye G et al	2018	BMC Women's Health	Ineligible objective
Maternal near miss and quality of care in a rural Rwandan hospital.	Kalisa R et al	2016	BMC Pregnancy and Childbirth	Ineligible objective
Neonatal hypothermia levels and risk factors for mortality in a tropical country	Kambarami R & Chidede O	2003	The Central African Journal of Medicine	Ineligible objective
Trends in maternal mortality ratio in a tertiary referral hospital and the effects of various maternity schemes on it	Kaur H et al	2015	Journal of Family and Reproductive Health	Ineligible objective
Obstetrical trauma to the genital tract following vaginal delivery	Khaskheli M et al	2012	Journal of the College of Physicians and Surgeons	Ineligible objective
Unplanned out-of-hospital birth and risk factors of adverse perinatal outcome: findings from a prospective cohort	Javaudin F et al	2019	Scandinavian Journal of Trauma, Resuscitation and Emergency Medicine	Ineligible objective
Maternal risk factors in early neonatal sepsis at a tertiary care teaching hospital	Javed M et al	2009	Saudi Medical Journal	Ineligible objective
Population-based surveillance of neonatal herpes simplex virus infection in Australia, 1997-2011	Jones CA et al	2014	Clinical Infectious Diseases	Ineligible objective
Essential ten life-saving skills preventing maternal death	Jesmin Z	2017	BJOG	Ineligible objective
The maternal mortality rate in al-diwaniyah province in iraq: Retrospective data retrieval of four years	Jabir HH et al	2018	International Journal of Research in Pharmaceutical Sciences	Ineligible objective
Obstetric admissions to tertiary level intensive care unit - prevalence, clinical characteristics and outcomes	Joseph CM et al	2018	Indian Journal of Anaesthesia	Ineligible objective
Incidence of death from congenital toxoplasmosis in 0-4-year-old children in Japan	Hoshino T et al	2014	Pediatrics International	Ineligible objective
Severe maternal morbidity and comorbid risk in hospitals performing <1000 deliveries per year	Hehir MP et al	2017	American Journal of Obstetrics and Gynecology	Ineligible objective
Peripartum bacteremia in the era of group B streptococcus prophylaxis	Cape A et al	2013	Obstetrics and Gynecology	Ineligible objective
The tip of the iceberg: evidence of seasonality in institutional maternal mortality and implications for health resources management in Burkina Faso	Hounton SH et al	2008	Scandinavian Journal of Public Health	Ineligible objective
Perinatal health outcomes and care among asylum seekers and refugees: a systematic review of systematic reviews	Heslehurst N et al	2018	BMC Medicine	Ineligible objective
Reducing maternal deaths in a low resource setting in Nigeria	Ezugwu EC et al	2014	Niger. J. Clin. Pract.	Ineligible objective
Time of delivery and neonatal morbidity and mortality	Caughey AB et al	2008	American Journal of Obstetrics and Gynecology	Ineligible objective
Prevalence of respiratory pathogens during two consecutive respiratory syncytial virus seasons at a tertiary medical care center	Celik K et al	2019	Arch. Argent. Pediatr.	Ineligible objective
Trends in maternal mortality in resident vs. migrant women in Shanghai, China, 2000-2009: a register-based analysis	Du L et al	2012	Reproductive Health Matters	Ineligible objective
Maternal and neonatal outcomes of adolescent pregnancy	Karatasli V et al	2019	Journal of Gynecology Obstetrics and Human Reproduction	Ineligible objective
Disparities and trends in birth outcomes, perinatal and infant mortality in Aboriginal vs. non-Aboriginal populations: a population-based study in Quebec, Canada 1996-2010	Chen L et al	2015	PLoS One	Ineligible objective
Maternal and fetal morbidity and mortality following multiple caesarean sections in northern Jordan	Hatamleh R et al	2017	Evidence Based Midwifery	Ineligible objective
Revisit of risk factors for major obstetric hemorrhage: insights from a large medical center	Helman S et al	2015	Archives of Gynecology and Obstetrics	Ineligible objective
The relationship between timing of postpartum hemorrhage interventions and adverse outcomes	Howard TF et al	2015	American Journal of Obstetrics and Gynecology	Ineligible objective
Timing of planned caesarean section and the morbidities of the newborn	Hourani M et al	2011	North American Journal of Medical Sciences	Ineligible objective
Misoprostol for prevention of postpartum hemorrhage at home birth in Afghanistan: program expansion experience	Haver J et al	2016	Journal of Midwifery & Women's Health	Ineligible objective
Application effect of sterile normal saline ice for post-partum hemorrhage at the time of cesarean delivery: a retrospective review	Cheng W et al	2016	The Journal of Obstetrics and Gynaecology Research	Ineligible objective
Early discharge and readmission to hospital in first six days of life	Dizdarevic J et al	2011	HealthMED	Ineligible objective
Outcomes of patients admitted to the intensive care unit for complications of hypertensive disorders of pregnancy at a South African tertiary hospital -- a 4-year retrospective review	Gama S et al	2019	Southern African Journal of Critical Care	Ineligible objective
Neonatal outcomes following elective caesarean delivery at term: a hospital-based cohort study	Finn D et al	2016	The Journal of Maternal-Fetal & Neonatal Medicine	Ineligible objective
Maternity wards or emergency obstetric rooms? Incidence of near-miss events in African hospitals	Filippi V et al	2005	Acta obstetricia et gynecologica Scandinavica	Ineligible objective
Maternal mortality due to hemorrhage in Brazil	de Souza MdL et al	2013	Revista latino-americana de enfermagem	Ineligible objective
Outcomes of second-line therapies for stage 3 postpartum hemorrhage at a tertiary care center	Clure C et al	2018	Obstetrics and Gynecology	Ineligible objective
Maternal death in the 21st century: causes, prevention, and relationship to cesarean delivery	Clark SL et al	2008	American Journal of Obstetrics and Gynecology	Ineligible objective
Trends in maternal mortality over 29 years in a Kuwait tertiary teaching hospital: signs of progress?	Chibber R et al	2012	The Journal of Maternal-Fetal & Neonatal Medicine	Ineligible objective
Duplex ultrasound screening for deep vein thrombosis in Chinese after cesarean section	Chan LY-S et al	2005	Acta obstetricia et gynecologica Scandinavica	Ineligible objective
Shock progression and survival after use of a condom uterine balloon tamponade package in women with uncontrolled postpartum hemorrhage	Burke TF et al	2017	International Journal of Gynaecology and Obstetrics	Ineligible objective
Maternal mortality in the Gaza strip: a look at causes and solutions	Bottcher B et al	2018	BMC Pregnancy and Childbirth	Ineligible objective
Ranitidine and late-onset sepsis in the neonatal intensive care unit	Bianconi S et al	2007	J. Perinat. Med.	Ineligible objective
Blood glucose levels in neonatal sepsis and probable sepsis and its association with mortality	Ahmad S & Khalid R	2012	Journal of the College of Physicians and Surgeons	Ineligible objective
Experience of maternal and perinatal death surveillance response in Nigeria using an e-platform	Galadanci et al	2018	International Journal of Gynecology and Obstetrics	Ineligible objective
Trends and outcomes of postpartum haemorrhage, 2003-2011	Ford JB et al	2015	BMC Pregnancy and Childbirth	Ineligible objective
Increased postpartum hemorrhage rates in Australia	Ford JB et al	2007	International Journal of Gynaecology and Obstetrics	Ineligible objective
Thirty seven weeks and beyond maternal and foetal outcome by week of gestation	Doppa GJ et al	2016	J. Evol. Med. Dent. Sci.	Ineligible objective
Birth in Brazil: national survey into labour and birth	do Carmo Leal M et al	2012	Reproductive Health	Ineligible objective
Antibiotic prophylaxis for caesarean section at a Ugandan hospital: a randomised clinical trial evaluating the effect of administration time on the incidence of postoperative infections	Dlamini LD et al	2015	BMC Pregnancy and Childbirth	Ineligible objective
Trends in maternal and newborn health characteristics and obstetric interventions among Aboriginal and Torres Strait Islander mothers in Western Australia from 1986 to 2009	Diouf I et al	2016	The Australian & New Zealand Journal of Obstetrics & Gynaecology	Ineligible objective
Rapid diagnosis of sepsis and bacterial meningitis in children with real-time fluorescent quantitative polymerase chain reaction amplification in the bacterial 16S rRNA gene	Chen L et al	2009	Clinical Pediatrics	Ineligible objective
Timing of delivery and adverse outcomes in term singleton repeat cesarean deliveries	Chiossi G et al	2013	Obstetrics and Gynecology	Ineligible objective
Mapping of research on maternal health interventions in low- and middle-income countries: a review of 2292 publications between 2000 and 2012	Chersich M et al	2016	Globalization and Health	Ineligible objective
Can a mortality excess in remote areas of Australia be explained by indigenous status? A case study using neonatal mortality in Queensland	Coory M	2003	Australian & New Zealand Journal of Public Health	Ineligible objective
Maternal mortality trends at the Princess Marina and Nyangabwe referral hospitals in Botswana	Nkhwalume,L & Mashalla Y	2019	Afr. Health Sci.	Ineligible objective
Changing risks of stillbirth and neonatal mortality associated with maternal age in Western Australia 1984-2003	O'Leary CM et al	2007	Paediatric and Perinatal Epidemiology	Ineligible objective
Maternal and newborn outcomes in Pakistan compared to other low and middle income countries in the Global Network's Maternal Newborn Health Registry: an active, community-based, pregnancy surveillance mechanism	Pasha O et al	2015	Reprod. Health	Ineligible objective
Perinatal mortality at Frontier Hospital, Queenstown - a 6-year audit using the Perinatal Problem Identification Programme [PPIP]	Patrick ME	2007	South African Journal of Obstetrics and Gynaecology	Ineligible objective
Maternal mortality at Muhimbili National Hospital in Dar-es-Salaam, Tanzania in the year 2011	Pembe AB et al	2014	BMC Pregnancy and Childbirth	Ineligible objective
Timing of initiation of breastfeeding and early-newborn sepsis: evidence from rural Bangladesh	Raihana S et al	2017	Annals of Nutrition and Metabolism	Ineligible objective
Early initiation of breastfeeding and severe illness in the early newborn period: an observational study in rural Bangladesh	Raihana S et al	2019	PLoS Medicine	Ineligible objective
A comprehensive assessment of maternal deaths in Argentina: translating multicentre collaborative research into action	Ramos S et al	2007	Bulletin of the World Health Organization	Ineligible objective
Maternal mortality over the last decade: a changing pattern of death due to alarming rise in hepatitis in the latter five-year period	Rana A et al	2009	The Journal of Obstetrics and Gynaecology Research	Ineligible objective
Somali women's use of maternity health services and the outcome of their pregnancies: a descriptive study comparing Somali immigrants with native-born Swedish women	Rassjo EV et al	2013	Sexual & Reproductive Healthcare	Ineligible objective
Surgical management of postpartum hemorrhage at in a tertiary hospital, Karnataka-a retrospective study	Ravipati P et al	2014	BJOG	Ineligible objective
Eclampsia: a neurological perspective	Shah AK et al	2008	Journal of the Neurological Sciences	Ineligible objective
Maternal and infant mortality in Mahottari district of Nepal	Shah R & Maskey MK	2010	Journal of Nepal Health Research Council	Ineligible objective
Frequency and outcome of eclampsia	Shaikh F et al	2016	Gomal J. Med. Sci.	Ineligible objective
Maternal deaths associated with hypertension in South Africa: lessons to learn from the Saving Mothers report, 2005-2007	Moodley J et al	2011	Cardiovascular Journal of Africa	Ineligible objective
When getting there is not enough: a nationwide cross-sectional study of 998 maternal deaths and 1451 near-misses in public tertiary hospitals in a low-income country	Oladapo OT et al	2016	BJOG	Ineligible objective
Implementation of the Alliance for Innovation on Maternal Health Program to Reduce Maternal Mortality in Malawi	Chang OH et al	2019	Obstetrics and Gynecology	Ineligible objective
Automated determination of neutrophil VCS parameters in diagnosis and treatment efficacy of neonatal sepsis	Celik IH et al	2012	Pediatric Research	Ineligible objective
Hypertensive disorders in pregnancy and maternal and neonatal outcomes in Haiti: the importance of surveillance and data collection	Bridwell M et al	2019	BMC Pregnancy Childbirth	Ineligible objective
Higher rate of serious perinatal events in non-Western women in Denmark	Brehm Christensen M et al	2016	Danish Medical Journal	Ineligible objective
A postpartum hemorrhage package with uterine balloon tamponade: a prospective multi-center case series in Kenya, Sierra Leone, Senegal, and Nepal	Burke T et al	2015	International Journal of Gynecology and Obstetrics	Ineligible objective
Comparison of subcuticular suture type in post-cesarean wound complications: a randomized controlled trial	Buresch A et al	2017	American Journal of Obstetrics and Gynecology	Ineligible objective
Should delivery timing for repeat cesarean be reconsidered based on dating criteria?	Brookfield KF et al	2019	The Journal of Maternal-Fetal & Neonatal Medicine	Ineligible objective
A systems approach for neonatal hyperbilirubinemia in term and near-term newborns	Bhutani VK et al	2006	Journal of Obstetric, Gynecologic & Neonatal Nursing	Ineligible objective
Impact of syndrome evaluaton system (SES) on outcomes of neonatal sepsis-a randomized-controlled trial	Bhat V et al	2015	Indian Journal of Critical Care Medicine	Ineligible objective
Effect of community-based newborn care on cause-specific neonatal mortality in Sylhet district, Bangladesh: findings of a cluster-randomized controlled trial	Baqui AH et al	2016	Journal of Perinatology	Ineligible objective
Cesarean delivery skin closure technique: comparison between staples and antibacterial knotless suture	Bleicher I et al	2019	American Journal of Obstetrics and Gynecology	Ineligible objective
AFLP versus HELLP syndrome: pregnancy outcomes and recovery	Byrne JJ et al	2019	American Journal of Obstetrics and Gynecology	Ineligible objective
Maternal mortality in New York City 1995-2003: disparities and risk factors	Campbell KH et al	2012	American Journal of Obstetrics and Gynecology	Ineligible objective
Maternal mortality at time of delivery hospitalization in large university-based hospitals in England, Australia, and the United States, 2007-2013	Campbell KH et al	2016	American Journal of Obstetrics and Gynecology	Ineligible objective
Maternal morbidity and risk of death at delivery hospitalization	Campbell KH et al	2013	Obstetrics and Gynecology	Ineligible objective
Trends in maternal mortality in Switzerland among Swiss and foreign nationals, 1969-2006	Bollini P et al	2011	International Journal of Public Health	Ineligible objective
Prevalence and severity of thrombocytopenia in blood culture proven neonatal sepsis: a prospective study	Bhat YR et al	2018	Archives of Pediatric Infectious Diseases	Ineligible objective
Dehydration and hypernatremia in breast-fed term healthy neonates	Bhat SR et al	2006	Indian Journal of Pediatrics	Ineligible objective
Prevalence and risk factors for early postpartum anemia	Bergmann RL et al	2010	European Journal of Obstetrics, Gynecology, and Reproductive Biology	Ineligible objective
Review of maternal mortality in Ethiopia: a story of the past 30 years	Berhan Y & Berhan A	2014	Ethiopian Journal of Health Sciences	Ineligible objective
Uterine compression sutures for postpartum hemorrhage: efficacy, morbidity, and subsequent pregnancy	Baskett TF	2007	Obstetrics and gynecology	Ineligible objective
Magnitude of maternal and neonatal mortality in Tanzania: a systematic review	Armstrong CE	2015	International Journal of Gynaecology and Obstetrics	Ineligible objective
Evaluation of measured postpartum blood loss after vaginal delivery using a collector bag in relation to postpartum hemorrhage management strategies: a prospective observational study	Bamberg C	2016	Journal of Perinatal Medicine	Ineligible objective
Secular trends in preeclampsia incidence and outcomes in a large Canada database: a longitudinal study over 24 years	Auger N et al	2016	The Canadian Journal of Cardiology	Ineligible objective
Results from the helping mothers survive study in Tanzania and Uganda	Baleke SA	2018	International Journal of Gynecology and Obstetrics	Ineligible objective
Near miss maternal morbidity - experience at a tertiary referral centre	Anandakrishnan S et al	2010	International Journal of Obstetric Anesthesia	Ineligible objective
Transporting newborns with subgaleal haemorrhage-the NSW experience	Amanda D et al	2016	Journal of Paediatrics and Child Health	Ineligible objective
Effects of delayed compared with early umbilical cord clamping on maternal postpartum hemorrhage and cord blood gas sampling: a randomized trial	Andersson O et al	2013	Acta obstetricia et gynecologica Scandinavica	Ineligible objective
Lowered national cesarean section rates after a concerted action	Ayres-De-Campos D et al	2015	Acta obstetricia et gynecologica Scandinavica	Ineligible objective
A retrospective comparison of waterbirth outcomes in two United States hospital settings	Bailey JM et al	2019	Birth	Ineligible objective
Vaginal birth after cesarean section	Bangal VB et al	2013	North American Journal of Medical Sciences	Ineligible objective
Effect of predelivery vaginal antisepsis on maternal and neonatal morbidity and mortality in Egypt	Bakr AF & Karkour T	2005	Journal of Women's Health	Ineligible objective
The effect of house staff working hours on the quality of obstetric and gynecologic care	Bailit JL & Blanchard MH	2004	Obstetrics and Gynecology	Ineligible objective
A randomised controlled trial of antibiotic prophylaxis in elective caesarean delivery	Bagratee JS et al	2001	BJOG	Ineligible objective
Identifying maternal deaths in Texas using an enhanced method, 2012	Baeva S et al	2018	Obstetrics and Gynecology	Ineligible objective
Short-course postpartum (6-h) magnesium sulfate therapy in severe preeclampsia	Anjum S et al	2016	Archives of Gynecology and Obstetrics	Ineligible objective
A nationwide descriptive study of obstetric claims for compensation in Norway	Andreasen S et al	2012	Acta obstetricia et gynecologica Scandinavica	Ineligible objective
Intra-hospital mortality among neonates transported by ambulance in Colombia	Alvarado-Socarras J et al	2014	Pediatrics International	Ineligible objective
Maternal outcomes in birth centers: an integrative review of the literature	Alliman J & Phillippi JC	2016	Journal of Midwifery & Women's Health	Ineligible objective
Obstetric and perinatal outcome of women para > or = 5 including one lower segment cesarean section	Ali AM & Abu-Heija AT	2002	The Journal of Obstetrics and Gynaecology Research	Ineligible objective
Maternal and perinatal outcomes with increasing duration of the second stage of labor	Allen VM et al	2009	Obstetrics and Gynecology	Ineligible objective
Comparison of maternal and infant outcomes from primary cesarean delivery during the second compared with first stage of labor	Alexander JM et al	2007	Obstetrics and Gynecology	Ineligible objective
Prevalence and risk factors of severe obstetric haemorrhage	Al-Zirqi I et al	2008	BJOG	Ineligible objective
Liver enzyme patterns in maternal deaths due to eclampsia: a South African cohort	Alese OM et al	2019	Pregnancy Hypertension	Ineligible objective
The neighbourhood method for measuring differences in maternal mortality, infant mortality and other rare demographic events	Alam N & Townend J	2014	PLoS One	Ineligible objective
Epidemiological characterization of serotype group B Streptococci neonatal infections associated with interleukin-6 level as a sensitive parameter for the early diagnosis	Al Hazzani AA et al	2018	Saudi Journal of Biological Sciences	Ineligible objective
Monitoring maternal, newborn, and child health interventions using lot quality assurance sampling in Sokoto State of northern Nigeria	Abegunde D et al	2015	Global Health Action	Ineligible objective
Maternal near miss: a valuable contribution in maternal care	Abha S et al	2016	Journal of Obstetrics and Gynaecology of India	Ineligible objective
Pelvic floor distress symptoms within 9 weeks of childbirth among Nigerian women	Adaji SE & Olajide FM	2014	European Journal of Obstetrics, Gynecology, and Reproductive Biology	Ineligible objective
Disparities between Aboriginal and non-Aboriginal perinatal mortality rates in Western Australia from 1980 to 2015	Adane AA et al	2019	Paediatric and Perinatal Epidemiology	Ineligible objective
An hour-specific transcutaneous bilirubin nomogram for Mongolian neonates	Akahira-Azuma M et al	2015	European Journal of Pediatrics	Ineligible objective
Multiple organ dysfunction score is superior to the obstetric-specific sepsis in obstetrics score in predicting mortality in septic obstetric patients	Aarvold ABR et al	2017	Critical Care Medicine	Ineligible objective
Level, causes and risk factors of neonatal mortality, in Jordan: results of a national prospective study	Batieha et al	2016	Matern Child Health J	Ineligible outcome
Neonatal mortality in a referral hospital in Cameroon over a seven year period: Trends, associated factors and causes	Mah EM et al	2014	African Health Sciences	Ineligible outcome
[Neonatal and perinatal mortality in hospitals of the Basque Country-Navarre Neonatal Study Group (GEN-VN) during the period 2000-2006]	Rada Fernandez de Jauregui D et al	2009	Anales de pediatria	Ineligible outcome
Assessment of incidence and factors associated with severe maternal morbidity after delivery discharge among women in the US	Chen J et al	2021	JAMA Network Open	Ineligible outcome
Near miss and maternal mortality at the Jos University Teaching Hospital	Samuels E et al	2020	Nigerian Medical Journal	Ineligible outcome
Adverse maternal and neonatal outcomes among low-risk women with obesity at 37-41 weeks gestation	Bicocca, Matthew J et al	2020	European Journal of Obstetrics, Gynecology, and Reproductive Biology	Ineligible outcome
Pregnancy outcomes in facility deliveries in Kenya and Uganda: a large cross-sectional analysis of maternity registers illuminating opportunities for mortality prevention	Waiswa P et al	2020	PLoS One	Ineligible outcome
[Early neonatal mortality and its determinants in a Level 1 maternity in Yaounde, Cameroon]	Chelo D et al	2012	The Pan African Medical Journal	Ineligible outcome
[The perinatal mortality in a general hospital]	Castaneda-Casale G et al	2010	Revista medica del Instituto Mexicano del Seguro Social	Ineligible outcome
Maternal and perinatal outcomes by mode of delivery in Senegal and Mali: a cross-sectional epidemiological survey	Briand V et al	2012	PLoS One	Ineligible outcome
Maternal mortality in the main referral hospital in Angola, 2010-2014: understanding the context for maternal deaths amidst poor documentation	Umar A	2016	International Journal of MCH and AIDS	Ineligible outcome
Emergency department visits for postpartum complications	Brousseau EC et al	2016	Obstetrics and Gynecology	Ineligible outcome
Maternal mortality at the Central Hospital, Benin City Nigeria: a ten year review	Abe E & Omo-Aghoja LO	2008	African Journal of Reproductive Health	Ineligible outcome
Postpartum venous thromboembolism readmissions in the United States	Wen T et al	2018	American Journal of Obstetrics and Gynecology	Ineligible outcome
Timing of postpartum readmissions and risk for severe maternal morbidity	Wen T et al	2019	American Journal of Obstetrics and Gynecology	Ineligible outcome
Timing and risk factors of postpartum stroke	Too G et al	2018	Obstetrics and Gynecology	Ineligible outcome
Incidence of neonatal hyperbilirubinemia: a population-based prospective study in Pakistan	Tikmani SS et al	2010	Trop. Med. Int. Health	Ineligible outcome
The incidence and outcome of bilirubin encephalopathy in Nigeria: a bi-centre study	Ogunlesi TA et al	2007	Nigerian Journal of Medicine	Ineligible outcome
Delayed postpartum preeclampsia: an experience of 151 cases.	Matthys LA et al	2004	American Journal of Obstetrics and Gynecology	Ineligible outcome
Prospective surveillance study of severe hyperbilirubinaemia in the newborn in the UK and Ireland	Manning D et al	2007	Archives of Disease in Childhood	Ineligible outcome
Study of changing trend in maternal mortality	Jyothi GS et al	2012	Perinatology	Ineligible outcome
Maternal and neonatal survival and mortality in the Upper West Region of Ghana	Issah K et al	2011	International Journal of Gynecology and Obstetrics	Ineligible outcome
Severe neonatal hyperbilirubinemia and adverse short-term consequences in Baghdad, Iraq	Hameed NN et al	2011	Neonatology	Ineligible outcome
Impact of discharge timings of healthy newborns on the rates and etiology of neonatal hospital readmissions	Habib HS	2013	Journal of the College of Physicians and Surgeons	Ineligible outcome
A multi-state analysis of postpartum readmissions in the United States	Clapp MA et al	2016	American Journal of Obstetrics and Gynecology	Ineligible outcome
Delayed postpartum preeclampsia and eclampsia: Demographics, clinical course, and complications	Al-Safi Z et al	2011	Obstetrics and Gynecology	Ineligible outcome
Maternal mortality at a referral centre: a five year study	Purandare N et al	2007	Journal of Obstetrics and Gynaecology of India	Ineligible outcome
Grim face of maternal mortality at tertiary care hospital of rural India: a 16 years study	Bangal Vidyadhar B et al	2013	Indian Journal of Public Health Research and Development	Ineligible outcome
Severe maternal morbidity in Canada, 1991-2001	Wen SW et al	2005	Can. Med. Assoc. J.	Ineligible outcome
Maternal death reviews at a rural hospital in Malawi.	Vink NM et al	2013	International journal of gynaecology and obstetrics	Ineligible outcome
Incidence of immediate postpartum hemorrhages in French maternity units: a prospective observational study (HERA study)	Vendittelli F et al	2016	BMC Pregnancy and Childbirth	Ineligible outcome
Changing trends in the causes of maternal mortality over the past 4 years in a tertiary care centre	Uma D et al	2017	J. Evol. Med. Dent. Sci.	Ineligible outcome
Incidence and outcomes of eclampsia: a single-center 30-year study	Uludag SZ et al	2019	Hypertension in pregnancy	Ineligible outcome
A five year retrospective study of maternal mortality at Rajendra Institute of Medical Sciences, Ranchi, Jharkhand in the year 2011 to 2015	Trivedi K & Prakash R	2016	J. Evol. Med. Dent. Sci.	Ineligible outcome
Birth outcomes among First Nations, Inuit and Metis populations	Sheppard AJ et al	2017	Health Rep.	Ineligible outcome
Venous thromboembolism during pregnancy and the post-partum period: incidence and risk factors in a large Victorian health service.	Sharma S & Monga D	2008	The Australian & New Zealand Journal of Obstetrics & Gynaecology	Ineligible outcome
Puerperal sepsis--still a major threat for parturient	Shamshad et al	2010	Journal of Ayub Medical College	Ineligible outcome
Population-based study of early-onset neonatal sepsis in Canada	Sgro M et al	2019	Paediatrics & Child Health	Ineligible outcome
A cross sectional study of maternal near miss and mortality at a rural tertiary centre in southern Nigeria	Mbachu II et al	2017	BMC Pregnancy and Childbirth	Ineligible outcome
Quantifying severe maternal morbidity in Scotland: a continuous audit since 2003	Marr L et al	2014	Current Opinion in Anaesthesiology	Ineligible outcome
Severe acute maternal morbidity: use of the Brazilian Hospital Information System	Magalhaes MD & Bustamante-Teixeira MT	2012	Rev. Saude Publica	Ineligible outcome
Incidence and determinants of severe maternal morbidity: a transversal study in a referral hospital in Teresina, Piaui, Brazil	Madeiro AP et al	2015	BMC Pregnancy Childbirth	Ineligible outcome
A critical analysis of maternal morbidity and mortality in Liberia, West Africa	Lori JR & Starke AE	2012	Midwifery	Ineligible outcome
Maternal near-miss and death and their association with caesarean section complications: a cross-sectional study at a university hospital and a regional hospital in Tanzania	Litorp H et al	2014	BMC Pregnancy Childbirth	Ineligible outcome
[A survey of neonatal births in maternity departments in urban China in 2005]	Li J et al	2012	Chinese Journal of Contemporary Pediatrics	Ineligible outcome
Incidence and causes of maternal mortality in the USA	Kuriya A et al	2016	The Journal of Obstetrics and Gynaecology Research	Ineligible outcome
Maternal mortality ratio and its causes in a district headquarter hospital of NWFP	Jabeen M et al	2005	Journal of Postgraduate Medical Institute	Ineligible outcome
[Analysis of death maternal cases during a 10-year period]	Hernandez Penafiel JA et al	2007	Ginecologia y obstetricia de Mexico	Ineligible outcome
Trends in caesarean section rates between 2007 and 2013 in obstetric risk groups inspired by the Robson classification: results from population-based surveys in a low-resource setting	Hanson C et al	2019	BJOG	Ineligible outcome
Maternal death: audit in a tertiary hospital	Guha K & Ashraf F	2019	Mymensingh Medical Journal	Ineligible outcome
Incidence, trends and severity of primary postpartum haemorrhage in Australia: a population-based study using Victorian Perinatal Data Collection data for 764 244 births	Flood M et al	2019	The Australian & New Zealand Journal of Obstetrics & Gynaecology	Ineligible outcome
[Analysis of trends in maternal mortality during a 10 year-follow up in a urban region]	Ferrer Arreola L et al	2005	Ginecologia y obstetricia de Mexico	Ineligible outcome
Maternal mortality in Italy: a record-linkage study	Donati S et al	2011	BJOG	Ineligible outcome
Causes of neonatal and child mortality in India: a nationally representative mortality survey	Bassani DG et al	2010	Lancet	Ineligible outcome
Maternal morbidity and mortality in San Carlos, Cojedes-Venezuela. 2001-2008	Aure N et al	2011	Salus	Ineligible outcome
Incidence and risk factors of sepsis mortality in labor, delivery and after birth: population-based study in the USA	Al-Ostad G et al	2015	The Journal of Obstetrics and Gynaecology Research	Ineligible outcome
Emergency peripartum hysterectomy: a multicenter study of incidence, indications and outcomes in southwestern Nigeria	Akintayo AA et al	2016	Maternal and Child Health Journal	Ineligible outcome
Health in Myanmar 2008	Suvedi BK et al	2009	Ministry of Health Report	Ineligible outcome
Trends and causes of maternal mortality in Eastern province of Turkey	Çim N et al	2017	Eastern Journal of Medicine	Ineligible outcome
Causes of stillbirths and early neonatal deaths: data from 7993 pregnancies in six developing countries	Ngoc NTN et al	2006	Bulletin of the World Health Organization	Ineligible outcome
Comparison of microbial pattern in early and late onset neonatal sepsis in referral center Haji Adam Malik hospital Medan Indonesia	Hasibuan BS	2018	IOP Science	Ineligible outcome
The most common causative bacteria in maternal sepsis-related deaths in Japan were group A Streptococcus: a nationwide survey	Tanaka H et al	2019	Journal of Infection and Chemotherapy	Ineligible outcome
Infant mortality in the Federal District, Brazil: time trend and socioeconomic inequalities	Monteiro RA et al	2007	Cadernos de saude publica	Ineligible outcome
Causes of child deaths in India, 1985-2008: a systematic review of literature.	Lahariya C et al	2010	Indian Journal of Pediatrics	Ineligible outcome
Sudden unexpected postnatal collapse of newborn infants: a review of cases, definitions, risks, and preventive measures	Herlenius E & Kuhn P	2013	Transl. Stroke Res.	Ineligible outcome
When do newborns die? A systematic review of timing of overall and cause-specific neonatal deaths in developing countries.	Sankar MJ et al	2016	Journal of Perinatology	Ineligible outcome
Still births, neonatal deaths and neonatal near miss cases attributable to severe obstetric complications: a prospective cohort study in two referral hospitals in Uganda	Nakimuli A et al	2015	BMC Pediatrics	Ineligible outcome
Early discharge of infants and risk of readmission for jaundice	Lain SJ et al	2015	Pediatrics	Ineligible outcome
Saving mothers' lives: reviewing maternal deaths to make motherhood safer: 2006-2008. The Eighth Report of the Confidential Enquiries into Maternal Deaths in the United Kingdom	Cantwell R et al	2011	BJOG	Ineligible outcome
Neonatal sepsis in rural India: timing, microbiology and antibiotic resistance in a population-based prospective study in the community setting	Panigrahi P et al	2017	Journal of Perinatology	Ineligible outcome
A prospective study of maternal mortality rate in tertiary care centre from 2010 to 2013 (a three year study)	Jabeen F et al	2016	BJOG	Ineligible outcome
Severe maternal morbidity at delivery and risk of hospital encounters within 6 weeks and 1 year postpartum	Harvey EM et al	2018	Journal of Women's Health	Ineligible outcome
Late-onset neonatal sepsis-a 10-year review from North Queensland, Australia.	Gowda H et al	2017	The Pediatric Infectious Disease Journal	Ineligible outcome
Neonatal hypothermia in Uganda: prevalence and risk factors	Byaruhanga R et al	2005	Journal of Tropical Pediatrics	Ineligible outcome
Causes, timing and place of neonatal deaths in rural Bangladesh	Azad K et al	2012	Journal of Paediatrics and Child Health	Ineligible outcome
Impact of risk factors on the timing of first postpartum venous thromboembolism: a population-based cohort study from England	Abdul Sultan A et al	2014	Blood	Ineligible outcome
The change of perinatal mortality over three decades in a reference centre in the aegean region: neonatal mortality has decreased but foetal mortality remains unchanged	Kultursay N et al	2017	Balkan Medical Journal	Ineligible outcome
[Perinatal mortality in the municipality of Salvador, Northeastern Brazil: evolution from 2000 to 2009]	Jacinto E et al	2013	Revista de saude publica	Ineligible outcome
Prevalence, serotype distribution and mortality risk associated with group B Streptococcus colonization of newborns in rural Bangladesh	Islam MS et al	2016	Pediatr. Infect. Dis. J.	Ineligible outcome
Thrombocytopenia in neonates: causes and outcomes	Ulusoy E et al	2013	Annals of Hematology	Ineligible outcome
Eclampsia in the period from 1983-2000: clinical aspects and maternal-perinatal health	Rodríguez Barredo M & Miguel JR	2003	Acta Ginecologica	Ineligible outcome
Neonatal sepsis: mortality in a municipality in southern Brazil, 2000 TO 2013	Alves JB et al	2018	Revista paulista de pediatria	Ineligible outcome
Incidence of neonatal sepsis in a sample of Iraqi newborns	Al-Mayah QS et al	2017	Pakistan Journal of Biotechnology	Ineligible outcome
Epidemiology of maternal mortality in France, 2010-2012	Deneux-tharaux C & Saucedo M	2018	Anesthesie et Reanimation	Ineligible outcome
Towards an inclusive and evidence-based definition of the maternal mortality ratio: an analysis of the distribution of time after delivery of maternal deaths in Mexico, 2010-2013	Lamadrid-Figueroa H et al	2016	PLoS One	Ineligible outcome
Incidence and risk factors for neonatal tetanus in admissions to Kilifi County Hospital, Kenya	Ibinda F et al	2015	PLoS One	Ineligible outcome
Factors associated with maternal deaths in district and Upazila hospitals of Bangladesh	Halim A et al	2016	Bangladesh Journal of Obstetrics and Gynecology	Ineligible outcome
Neonatal mortality and its risk factors in Eastern Ethiopia: a prospective cohort study in Kersa Health and Demographic Surveillance System (Kersa HDSS)	Desta BN et al	2016	Epidemiol. Biostat. Public Health	Ineligible outcome
Bacteriological profile of neonatal sepsis in neonatal intermediate care unit of central paediatric referral hospital in Nepal	Chapagain RH et al	2015	Journal of Nepal Health Research Council	Ineligible outcome
Changes in fetal and neonatal mortality during 40 years by offspring sex: a national registry-based study in Norway	Carlsen F et al	2013	BMC Pregnancy and Childbirth	Ineligible outcome
Temporal variations in incidence andoutcomes of critical illness among pregnant and postpartum women in Canada: a population-based observational study	Aoyama K et al	2019	Journal of Obstetrics and Gynaecology Canada	Ineligible outcome
Skilled attendant at birth and newborn survival in Sub-Saharan Africa	Amouzou A et al	2017	Journal of Global Health	Ineligible outcome
Place of birth or place of death: an evaluation of 1139 maternal deaths in Nigeria	Adegoke AA et al	2013	Midwifery	Ineligible outcome
An investigation of maternal mortality at a tertiary hospital of the Limpopo province of South Africa	Ntuli ST et al	2017	Southern African Journal of Infectious Diseases	Ineligible outcome
Verbal autopsy of neonatal deaths in Khatauli Block of District Muzaffarnagar, Uttar Pradesh, India	Muzammil K et al	2014	Nepal Journal of Epidemiology	Ineligible outcome
Maternal mortality in Central Province, Kenya, 2009-2010	Muchemi OM et al	2014	The Pan African Medical Journal	Ineligible outcome
Characteristics and outcomes of patients with eclampsia and severe pre-eclampsia in a rural hospital in Western Tanzania: a retrospective medical record study	Mooij R et al	2015	BMC Pregnancy and Childbirth	Ineligible outcome
[Maternal mortality in Libreville, Gabon: assessment and challenges]	Mayi-Tsonga S et al	2008	Sante	Ineligible outcome
Neonatal bacteraemia among 112,360 live births	Huggard D et al	2016	Irish Medical Journal	Ineligible outcome
Maternal mortality and derivations from the WHO near-miss tool: an institutional experience over a decade in Southern India	Halder A et al	2014	Journal of the Turkish-German Gynecological Association	Ineligible outcome
Maternal mortality in Herat Province, Afghanistan, in 2002: an indicator of women's human rights	Amowitz LL et al	2002	JAMA	Ineligible outcome
Pre-eclampsia-eclampsia admitted to critical care unit	Rojas-Suarez J & Vigil-De Gracia P	2012	The Journal of Maternal-Fetal & Neonatal Medicine	Ineligible outcome
The obstetric outcomes in women with preeclampsia and superimposed preeclampsia	Simsek A et al	2017	Turkiye Klinikleri Jinekoloji Obstetrik	Ineligible outcome
[Analysis of maternal deaths in Mexico occurred during 2009]	Fajardo-Dolci G et al	2013	Revista medica del Instituto Mexicano del Seguro Social	Ineligible outcome
Surveillance for incidence and etiology of early-onset neonatal sepsis in Soweto, South Africa	Velaphi SC et al	2019	PLoS One	Ineligible outcome
[Maternal mortality at the Centre De Sante Roi Baudouin (Dakar - Senegal): About 308 Cases]	Thiam O et al	2014	Le Mali Medical	Ineligible outcome
Fetal, neonatal, and post-neonatal mortality in the 2015 Pelotas (Brazil) birth cohort and associated factors	Varela AR et al	2019	Cadernos De Saude Publica	Ineligible outcome
Intraventricular hemorrhage in asphyxiated newborns treated with hypothermia: a look into incidence, timing and risk factors	Al Yazidi G et al	2015	BMC Pediatrics	Ineligible outcome
Early-onset neonatal infections in Australia and New Zealand, 2002-2012	Singh T et al	2019	Arch. Dis. Child.-Fetal Neonatal Ed.	Ineligible outcome
Infant mortality in three population-based cohorts in Southern Brazil: trends and differentials	Santos IS et al	2008	Cad. Saude Publica	Ineligible outcome
Prevalence of maternal morbidity and its association with socioeconomic factors: a population-based survey of a city in Northeastern Brazil	Rosendo TS et al	2017	Rev. Bras. Ginecol. Obstet.	Ineligible outcome
Descriptive epidemiology of neonatal mortality in Gowa District 2015	Putri AR et al	2018	International Conference on Healthcare Service Management	Ineligible outcome
Incidence, causes and correlates of maternal near-miss morbidity: a multi-centre cross-sectional study	Oppong, SA et al	2019	BJOG	Ineligible outcome
Primary postpartum haemorrhage in federal medical centre, Owerri, Nigeria: a six year review	Onyema OA et al	2015	Nigerian Journal of Medicine	Ineligible outcome
Postpartum hemorrhage: incidence, risk factors, and outcomes in a low-resource setting	Ngwenya S	2016	International Journal of Women's Health	Ineligible outcome
Maternal deaths due to hypertensive disorders of pregnancy: data from the 2014-2016 Saving Mothers' Report	Moodley J	2018	Obstetrics and Gynaecology Forum	Ineligible outcome
Infant mortality trends in the State of Rio Grande do Sul, Brazil, 1994-2004: a multilevel analysis of individual and community risk factors	Zanini RR et al	2009	Cad. Saude Publica	Ineligible outcome
Perinatal outcomes of severe preeclampsia/eclampsia and associated factors among mothers admitted in Amhara Region referral hospitals, North West Ethiopia, 2018	Melese MF et al	2019	BMC Research Notes	Ineligible outcome
A one year review of eclampsia in an Ethiopian Tertiary Care Center (Saint Paul's Hospital Millennium Medical College, SPHMMC)	Mekuria T & Abdosh A	2017	Journal of Perinatal Medicine	Ineligible outcome
Trends in postpartum hemorrhage from 2000 to 2009: a population-based study	Mehrabadi A et al	2012	BMC Pregnancy and Childbirth	Ineligible outcome
Maternal death audit in Rwanda 2009-2013: a nationwide facility-based retrospective cohort study	Sayinzoga F et al	2016	BMJ Open	Ineligible outcome
Serious bacterial infections in neonates presenting afebrile with history of fever	Ramgopal S et al	2019	Pediatrics	Ineligible outcome
Early neonatal streptococcal infection	Niduvaje K et al	2006	Indian Journal of Pediatrics	Ineligible outcome
Changing trends in maternal mortality in a developing country	Onakewhor JUE & Gharoro EP	2008	Nigerian Journal of Clinical Practice	Ineligible outcome
Frequency and timing of symptoms in infants screened for sepsis: effectiveness of a sepsis-screening pathway	Madan A et al	2003	Clinical Pediatrics	Ineligible outcome
[Time-course of neonatal precocious mortality between 1994 and 2003 at the Dakar University Teaching Hospital]	Cisse CT et al	2006	Journal de gynecologie, obstetrique et biologie de la reproduction	Ineligible outcome
Trends and causes of maternal mortality in jimma university specialized hospital, southwest ethiopia: a matched case-control study	Legesse T et al	2017	International Journal of Women's Health	Ineligible outcome
Epidemiological analysis of maternal deaths in Hunan province in China between 2009 and 2014	Lili X et al	2018	PLoS One	Ineligible outcome
[Maternal mortality. Experience of five years in Northern Veracruz IMSS Delegation]	Leal LAC et al	2009	Ginecologia y obstetricia de Mexico	Ineligible outcome
Impact and risk factors for early-onset group B streptococcal morbidity: analysis of a national, population-based cohort in Sweden 1997-2001.	Håkansson S et al	2006	BJOG	Ineligible outcome
Maternal mortality after cesarean section in the Netherlands	Kallianidis AF et al	2018	European Journal of Obstetrics & Gynecology & Reproductive Biology	Ineligible outcome
High maternal and neonatal mortality rates in northern Nigeria: an 8-month observational study	Guerrier G et al	2013	International Journal of Women's Health	Ineligible outcome
Trends in the modes of delivery and their impact on perinatal mortality rates	Duarte G et al	2004	Revista de saude publica	Ineligible outcome
Maternal mortality at a teaching hospital of rural India: a retrospective study	Das R & Mukherjee A	2014	BJOG	Ineligible outcome
Anesthetic management as a risk factor for postpartum hemorrhage after cesarean deliveries	Chang CC et al	2011	American Journal of Obstetrics & Gynecology	Ineligible outcome
Three years of neonatal morbidity and mortality at the national hospital in Dili, East Timor	Bucens IK et al	2013	Journal of Paediatrics and Child Health	Ineligible outcome
What about the mothers? An analysis of maternal mortality and morbidity in perinatal health surveillance systems in Europe	Bouvier-Colle M-H et al	2012	BJOG	Ineligible outcome
Venous thromboembolism during pregnancy, postpartum or during contraceptive use Findings from the RIETE Registry	Blanco-Molina A et al	2010	Thromb. Haemost.	Ineligible outcome
Rate and time trend of perinatal, infant, maternal mortality, natality and natural population growth in Kosovo	Azemi M et al	2012	Materia Socio-medica	Ineligible outcome
Cesarean section with relative indications versus spontaneous vaginal delivery: short-term outcomes of maternofetal health	Arikan I et al	2012	Clinical and Experimental Obstetrics & Gynecology	Ineligible outcome
Prevalence and associated factors of neonatal mortality in North Gondar Zone, Northwest Ethiopia	Kebede B et al	2012	Ethiop. J. Health Dev.	Ineligible outcome
A glance into the hidden burden of maternal morbidity and patterns of management in a Palestinian governmental referral hospital	Hassan SJ et al	2015	Women & Birth	Ineligible outcome
Maternal mortality in Pakistan--compilation of available data	Jafarey SN	2002	The Journal of the Pakistan Medical Association	Ineligible outcome
Prevalence and etiology of perinatal period mortality rates in hospitals, Iran	Jahani MA et al	2016	Research Journal of Medical Sciences	Ineligible outcome
Eclampsia: ten-years of experience in a rural tertiary hospital in the Niger delta, Nigeria	Igberase GO & Ebeigbe PN	2006	Journal of Obstetrics and Gynaecology	Ineligible outcome
Incidence, indications, and predictors of adverse outcomes of postpartum hysterectomies: 20-year experience in a tertiary care centre	Ibrahim M et al	2014	Journal of Obstetrics and Gynaecology Canada	Ineligible outcome
Trends and determinants of perinatal mortality in Bangladesh	Hossain MB et al	2019	PLoS One	Ineligible outcome
Prevalence of neutropenia in cases of neonatal sepsis	Ahmad MS et al	2017	Pakistan Paediatric Journal	Ineligible outcome
Trends in perinatal deaths from 2010 to 2013 in the Guatemalan Western Highlands	Garces A et al	2015	Reprod. Health	Ineligible outcome
Autopsy-certified maternal mortality at Ile-Ife, Nigeria	Dinyain A et al	2013	International Journal of Women's Health	Ineligible outcome
The clinical and bacteriogical spectrum of neonatal sepsis in a tertiary hospital in Yaounde, Cameroon	Chiabi A et al	2011	Iranian Journal of Pediatrics	Ineligible outcome
Eclapmsia: the major cause of maternal mortality in Eastern India	Das R & Biswas S	2015	Ethiopian Journal of Health Sciences	Ineligible outcome
Rates of obstetric intervention and associated perinatal mortality and morbidity among low-risk women giving birth in private and public hospitals in NSW (2000-2008): a linked data population-based cohort study	Dahlen HG et al	2014	BMJ Open	Ineligible outcome
The etiology of maternal mortality in developed countries: a systematic review of literature	Cristina Rossi A & Mullin P	2012	Archives of Gynecology and Obstetrics	Ineligible outcome
Ten years of confidential inquiries into maternal deaths in France, 1998-2007	Saucedo M et al	2013	Obstetrics and gynecology	Ineligible outcome
Changing epidemiology of maternal mortality in rural India: time to reset strategies for MDG-5	Shah P et al	2014	Tropical Medicine & International Health	Ineligible outcome
Maternal mortality in Andaman and Nicobar Group of Islands: 10 years retrospective study	Chawla I et al	2014	Indian Journal of Community Medicine	Ineligible outcome
Maternal morbidity associated with cesarean delivery without labor compared with spontaneous onset of labor at term	Allen VM et al	2003	Obstetrics & Gynecology	Ineligible outcome
Non-obstetric causes of severe maternal complications: a secondary analysis of the Nigeria Near-miss and Maternal Death Survey	Adeniran AS et al	2019	BJOG	Ineligible outcome
Pre- eclampsia, eclampsia and adverse maternal and perinatal outcomes: a secondary analysis of the World Health Organization Multicountry Survey on Maternal and Newborn Health	Abalos E et al	2014	BJOG	Ineligible outcome
The Spanish National Network "Grupo Castrillo": 22 Years of Nationwide Neonatal Infection Surveillance	Fernandez Colomer B et al	2020	American Journal of Perinatology	Ineligible population
Maternal and neonatal characteristics in obstetric intensive care unit admissions	Seppanen PM et al	2020	Int. J. Obstet. Anesth.	Ineligible population
Mortality at the pediatric emergency unit of the Mohammed VI teaching hospital of Marrakech	Lahmini W;& Bourrous M	2020	BMC Emergency Medicine	Ineligible population
Causes of neonatal death in ayder comprehensive specialized hospital, Ethiopia	Hadgu FB & Gebrekidan GB	2020	Iranian Journal of Neonatology	Ineligible population
Neonatal near-misses in Ghana: a prospective, observational, multi-center study	Bakari A et al	2019	BMC Pediatrics	Ineligible population
Timing and causes of neonatal mortality in Tamale Teaching Hospital, Ghana: a retrospective study	Abdul-Mumin A et al	2021	PLoS One	Ineligible population
[Eclampsia: epidemiological aspects and management of 28 patients]	Boudaya F et al	2008	La Tunisie medicale	Ineligible population
[Nine cases of HELLP syndrome (hemolysis, elevated liver enzymes and low platelets]	Capellino MF et al	2003	Medicina	Ineligible population
Etiology, antibiotic resistance and risk factors for neonatal sepsis in a large referral center in Zambia	Kabwe M et al	2016	The Pediatric Infectious Disease Journal	Ineligible population
Eclampsia in Finland; 2006 to 2010	Jaatinen N & Ekholm E	2016	Acta obstetricia et gynecologica Scandinavica	Ineligible population
Overview of maternal morbidity during hospitalization for labor and delivery in the United States: 1993-1997 and 2001-2005	Berg CJ et al	2009	Obstetrics and Gynecology	Ineligible population
Emergency department care in the postpartum period: California Births, 2009-2011	Batra P et al	2017	Obstetrics and Gynecology	Ineligible population
Abdominal massage: another cause of maternal mortality	Ugboma HAA & Akani CI	2004	Nigerian Journal of Medicine	Ineligible population
Clinical evaluation of severe neonatal hyperbilirubinaemia in a resource-limited setting: a 4-year longitudinal study in south-East Nigeria	Osuorah CDI et al	2018	BMC Pediatrics	Ineligible population
Maternal morbidity associated with early-onset and late-onset preeclampsia	Lisonkova S et al	2014	Obstetrics and Gynecology	Ineligible population
Infant outcome after complete uterine rupture	Al-Zirqi I et al	2018	American Journal of Obstetrics and Gynecology	Ineligible population
Neonatal nosocomial infections in Bahrami Children Hospital	Salamati P et al	2006	Indian Journal of Pediatrics	Ineligible population
A survey of the incidence of neonatal sepsis by group B Streptococcus during a decade in a Brazilian maternity hospital	Vaciloto E et al	2002	The Brazilian Journal of Infectious Diseases	Ineligible population
Length of rupture of membranes in the setting of premature rupture of membranes at term and infectious maternal morbidity	Tran SH et al	2008	Am. J. Obstet. Gynecol.	Ineligible population
Neonatal infections in England: the NeonIN surveillance network	Vergnano S et al	2011	Archives of Disease in Childhood	Ineligible population
Neonatal outcome following elective cesarean section beyond 37 weeks of gestation: a 7-year retrospective analysis of a national registry	Wilmink FA et al	2010	American Journal of Obstetrics and Gynecology	Ineligible population
Role of vascularization in determining the time of hypoxic-ischemic encephalopathy in the neonate	Aktas EO et al	2003	Analytical and Quantitative Cytology and Histology	Ineligible population
Admissions to a sick new born care unit in a secondary care hospital: profile and outcomes	Sinha RS et al	2019	Indian Journal of Public Health	Ineligible population
Patients with high-risk pregnancies and complicated deliveries have an increased risk of maternal postpartum readmissions	Sharvit M et al	2014	Archives of Gynecology and Obstetrics	Ineligible population
A cohort analysis of neonatal hospital mortality rate and predictors of neonatal mortality in a sub-urban hospital of Cameroon	Ndombo PK et al	2017	Italian Journal of Pediatrics	Ineligible population
Outcome of neonates with meconium aspiration syndrome at the University Hospital of the West Indies, Jamaica: a resource-limited setting	Panton L & Trotman H	2017	American Journal of Perinatology	Ineligible population
Post-operative management in uncomplicated caesarean delivery: a randomised trial of short-stay versus traditional protocol at the Lagos University Teaching Hospital, Nigeria	Oyeyemi N et al	2019	The Nigerian Postgraduate Medical Journal	Ineligible population
Pattern and outcome of obstetric admissions into the intensive care unit of a Southeast Nigerian hospital	Ozumba BC et al	2018	Indian Journal of Critical Care Medicine	Ineligible population
Early-onset neonatal sepsis: rate and organism pattern between 2003 and 2008	Sgro M et al	2011	Journal of Perinatology	Ineligible population
The burden of maternal morbidity and mortality attributable to hypertensive disorders in pregnancy: a prospective cohort study from Uganda	Nakimuli A et al	2016	BMC Pregnancy & Childbirth	Ineligible population
Teenage pregnancy: incidence and outcomes in a rural Shropshire district general hospital trust	Moores KL et al	2015	BJOG	Ineligible population
Duration of passive and active phases of the second stage of labour and risk of severe postpartum haemorrhage in low-risk nulliparous women	Le Ray C et al	2011	European Journal of Obstetrics, Gynecology, and Reproductive Biology	Ineligible population
Global, regional, and national causes of child mortality in 2000-13, with projections to inform post-2015 priorities: an updated systematic analysis	Liu L et al	2015	Lancet	Ineligible population
Clinical sepsis in neonates and young infants, United States, 1988-2006	Lukacs SL & Schrag SJ	2012	The Journal of Pediatrics	Ineligible population
Severe maternal morbidity during childbirth hospitalisation: a comparative analysis between the Republic of Ireland and Australia	Lutomski JE et al	2012	Eur. J. Obstet. Gynecol. Reprod. Biol.	Ineligible population
Comparison of clinical and perinatal outcomes in early- and late-onset preeclampsia	Madazli R et al	2014	Archives of Gynecology and Obstetrics	Ineligible population
Pregnancy-related mortality in California: causes, characteristics, and improvement opportunities	Main EK et al	2015	Obstetrics and Gynecology	Ineligible population
Treatment patterns and short-term outcomes in ischemic stroke in pregnancy or postpartum period	Leffert LR et al	2016	American Journal of Obstetrics and Gynecology	Ineligible population
A comparative study between the pioneer cohort of waterbirths and conventional vaginal deliveries in an obstetrician-led unit in Singapore	Lim KMX et al	2016	Taiwan. J. Obstet. Gynecol.	Ineligible population
Survery of care environment and mortality in a tertiary neonatal intensive care unit	Lee Y-S & Chou Y-H	2005	Clinical Neonatology	Ineligible population
Cause of death among infants in rural Western China: a community-based study using verbal autopsy	Ma Y et al	2014	J. Pediatr.	Ineligible population
Evaluation of infants with neonatal cholestasis: experience of a tertiary referral center in Turkey	Gürlek Gökçebay D et al	2015	Turkiye Klinikleri Tip Bilimleri Dergisi	Ineligible population
Early onset neonatal sepsis	Chacko B et al	2005	Indian Journal of Pediatrics	Ineligible population
Causes of perinatal mortality and associated maternal complications in a South African province: challenges in predicting poor outcomes	Allanson EM et al	2015	BMC Pregnancy and Childbirth	Ineligible population
Pattern of admissions to neonatal unit	Parkash J & Das N	2005	Journal of the College of Physicians and Surgeons	Ineligible population
Emergency peripartum hysterectomy: a 10-year review at the Royal Hospital for Women, Sydney	Awan N et al	2011	The Australian & New Zealand Journal of Obstetrics & Gynaecology	Ineligible population
Neonatal septic arthritis in a tertiary care hospital: a descriptive study	Sreenivas T et al	2016	European Journal of Orthopaedic Surgery & Traumatology	Ineligible population
Vasa previa diagnosis, clinical practice, and outcomes in Australia	Sullivan EA et al	2017	Obstetrics and Gynecology	Ineligible population
Maternal mortality and associated near-misses among emergency intrapartum obstetric referrals in Mulago Hospital, Kampala, Uganda	Kaye D et al	2003	East African Medical Journal	Ineligible population
Diurnal variation in decision-to-delivery intervals and correlation with adverse outcomes at emergency caesarean section in urban Uganda: a prospective cohort study	Hughes N et al	2019	BJOG	Ineligible population
Monitoring maternal and newborn health outcomes in Bauchi State, Nigeria: an evaluation of a standards-based quality improvement intervention	Kabo I et al	2016	International Journal for Quality in Health Care	Ineligible population
The chasm in neonatal outcomes in relation to time of birth in Lebanon	Badr LK et al	2007	Neonatal Network	Ineligible population
Abnormal bleeding associated with preeclampsia: a population study of 315,085 pregnancies	Eskild A & Vatten LJ	2009	Acta obstetricia et gynecologica Scandinavica	Ineligible population
WHO systematic review of randomised controlled trials of routine antenatal care	Carroli G et al	2001	Lancet	Ineligible population
The timing of elective caesarean deliveries and early neonatal outcomes in singleton infants born 37-41 weeks' gestation	Doan E et al	2014	The Australian & New Zealand Journal of Obstetrics & Gynaecology	Ineligible population
Neonatal nosocomial bloodstream infections at a referral hospital in a middle-income country: burden, pathogens, antimicrobial resistance and mortality	Dramowski A et al	2015	Paediatrics and International Child Health	Ineligible population
Balloon catheter for induction of labor in women with one previous cesarean and an unfavorable cervix.	Huisman CMA et al	2019	Acta obstetricia et gynecologica Scandinavica	Ineligible population
Neonatal complications in women with premature rupture of membranes (PROM) at term and near term and its correlation with time lapsed since PROM to delivery	Gupta S et al	2019	Tropical Doctor	Ineligible population
The evaluation of reasons for early or late onset neonatal thrombocytopenia	Guzoglu N et al	2015	Journal of Perinatal Medicine	Ineligible population
Incidence and organisam pattern in early onset neonatal sepsis	Hajnal Avramovic LZ et al	2012	Archives of Disease in Childhood	Ineligible population
Eclampsia: feto-maternal outcomes in a tertiary care centre in Eastern Nepal	Ghimire S	2016	Journal of the Nepal Medical Association	Ineligible population
Patterns of Infant Mortality from 1993 to 2007 in Belgrade (Serbia)	Gazibara T et al	2013	Maternal & Child Health Journal	Ineligible population
Neonatal hypoxic-ischaemic encephalopathy: most deaths followed end-of-life decisions within three days of birth	Garcia-Alix A et al	2013	Acta Paediatrica	Ineligible population
Patterns of admission and factors associated with neonatal mortality among neonates admitted to the neonatal intensive care unit of University of Gondar Hospital, Northwest Ethiopia	Demisse AG et al	2017	Pediatric Health, Medicine and Therapeutics	Ineligible population
Causes and risk factors for infant mortality in Nunavut, Canada 1999-2011	Collins SA et al	2012	BMC Pediatrics	Ineligible population
The burden of indirect causes of maternal morbidity and mortality in the process of obstetric transition: a cross-sectional multicenter study	Cirelli JF et al	2018	Rev. Bras. Ginecol. Obstet.	Ineligible population
Incidence of maternal near miss in the public health sector of Harare, Zimbabwe: a prospective descriptive study	Chikadaya H et al	2018	BMC Pregnancy and Childbirth	Ineligible population
Maternal morbidity in the first year after childbirth in Mombasa Kenya; a needs assessment	Chersich MF et al	2009	BMC Pregnancy and Childbirth	Ineligible population
Clinical analysis of emergency exploratory laparotomy in patients with intractable postpartum hemorrhage	Chen LC et al	2020	J. Int. Med. Res	Ineligible population
Maternal death and delays in accessing emergency obstetric care in Mozambique	Chavane LA et al	2018	BMC Pregnancy and Childbirth	Ineligible population
Maternal deaths: a 22-year forensic retrospective study (1987-2009)	Charlier P et al	2011	Revue de Medecine Legale	Ineligible population
The assessment of time-dependent myocardial changes in infants with perinatal hypoxia	Cetin I et al	2012	The Journal of Maternal-Fetal & Neonatal Medicine	Ineligible population
Neonatal outcomes after introduction of a national intrapartum fetal surveillance education program: a retrospective cohort study	Brown LD et al	2017	The Journal of Maternal-Fetal & Neonatal Medicine	Ineligible population
Eclampsia: still a problem in Bangladesh	Begum MR et al	2004	MedGenMed	Ineligible population
Post operative neonatal survival a real challenge in a setup with no intensive care unit!	Akhter N et al	2016	Rawal Medical Journal	Ineligible population
Epidemiology and microbiology of sepsis in mainland China in the first decade of the 21st century	Chen X-C et al	2015	International Journal of Infectious Diseases	Ineligible population
Adherence to hypothermia guidelines: a French multicenter study of fullterm neonates	Chevallier M et al	2013	PLoS One	Ineligible population
Incidence of catheter-related bloodstream infections in neonates following removal of peripherally inserted central venous catheters	Casner M et al	2014	Pediatric Critical Care Medicine	Ineligible population
The epidemiology of methicillin-susceptible and methicillin-resistant Staphylococcus aureus in a neonatal intensive care unit, 2000-2007	Carey AJ et al	2010	Journal of Perinatology	Ineligible population
New insights into Citrobacter freundii sepsis in neonates	Chen D & Ji Y	2019	Pediatrics International	Ineligible population
Timing of neonatal seizures and intrapartum obstetrical factors	Scher MS et al	2008	Journal of Child Neurology	Ineligible population
Planned early birth versus expectant management (waiting) for prelabour rupture of membranes at term (37 weeks or more)	Middleton P et al	2017	Cochrane Database of Systematic Reviews	Ineligible population
Factors associated with maternal death in women admitted to an intensive care unit with severe maternal morbidity	Oliveira Neto AF et al	2009	International Journal of Gynaecology and Obstetric	Ineligible population
Maternal and perinatal complications with uterine rupture in 142,075 patients who attempted vaginal birth after cesarean delivery: a review of the literature	Chauhan SP et al	2003	American Journal of Obstetrics and Gynecology	Ineligible population
Ventilator-associated pneumonia in newborn infants diagnosed with an invasive bronchoalveolar lavage technique: a prospective observational study	Cernada,M et al	2013	Pediatric Critical Care Medicine	Ineligible population
Nosocomial infections in a Brazilian neonatal intensive care unit: a 4-year surveillance study	Brito DV et al	2010	Rev. Soc. Bras. Med. Trop.	Ineligible population
Maternal and neonatal outcome after failed ventouse delivery: comparison of forceps versus cesarean section	Bhide A et al	2007	J. Matern.-Fetal Neonatal Med.	Ineligible population
A multicentre, randomised controlled trial of position during the late stages of labour in nulliparous women with an epidural: clinical effectiveness and an economic evaluation (BUMPES)	Bick D et al	2017	Health Technology Assessment	Ineligible population
Changing patterns in neonatal Escherichia coli sepsis and ampicillin resistance in the era of intrapartum antibiotic prophylaxis	Bizzarro MJ et al	2008	Pediatrics	Ineligible population
Seventy-five years of neonatal sepsis at Yale: 1928-2003	Bizzarro MJ et al	2005	Pediatrics	Ineligible population
Neonatal sepsis 2004-2013: the rise and fall of coagulase-negative staphylococci	Bizzarro MA et al	2015	The Journal of Pediatrics	Ineligible population
Approach to an obstetric prognosis scale: the modified SOFA scale	Blanco Esquivel LA et al	2016	Ghana Medical Journal	Ineligible population
Epidemiology of UK neonatal infections: the neonIN infection surveillance network	Cailes B et al	2018	Archives of Disease in Childhood	Ineligible population
Isolated proteinuria in Chinese pregnant women with pre-eclampsia: results of retrospective observational study	Cai J et al	2017	Biomedical Research	Ineligible population
Catheter-related infections in neonatal intensive care units: a prospective multicentre surveillance	Bellemin K et al	2011	BMC Proceedings	Ineligible population
A population-based study of perinatal infection risk in women with and without systemic lupus erythematosus and their infants	Bender Ignacio RA et al	2018	Paediatric & Perinatal Epidemiology	Ineligible population
Trends in mortality in a regional neonatal unit over 21 years demonstrate a halving of neonatal deaths	Benham VJI & Richards GJ	2014	Archives of Disease in Childhood	Ineligible population
Complications and maternal mortality from severe pre-eclampsia during the first 48 hours in an intensive care unit in Morocco	Bentata Y et al	2015	International Journal of Gynaecology and Obstetrics	Ineligible population
Epinephrine versus dopamine in neonatal septic shock: a double-blind randomized controlled trial	Baske K et al	2018	European Journal of Pediatrics	Ineligible population
Maternal and perinatal outcomes of eclampsia with and without HELLP syndrome in a teaching hospital in western Turkey	Asicioglu O et al	2014	Journal of Obstetrics and Gynaecology	Ineligible population
Determinants of nosocomial infection in 6 neonatal intensive care units: an Italian multicenter prospective cohort study	Auriti C et al	2010	Infection Control and Hospital Epidemiology	Ineligible population
Neonatal coronary artery thrombosis in the era of delayed umbilical cord clamping category:pPediatric	Aljohani O et al	2018	Catheterization and Cardiovascular Interventions	Ineligible population
Impact of cesarean section in a private health service in Brazil: indications and neonatal morbidity and mortality rates	Almeida MA et al	2018	Ceska gynekologie	Ineligible population
The correlation between invasive care procedures and the occurrence of neonatal sepsis	Andrade Medeiros F et al	2016	Acta Paulista de Enfermagem	Ineligible population
Neonatal hypothermia among hospitalized high risk newborns in a developing country	Ali R et al	2012	Pakistan Journal of Medical Sciences	Ineligible population
MR imaging and outcome of term neonates with perinatal asphyxia: value of diffusion-weighted MR imaging and H-1 MR spectroscopy	Alderliesten T et al	2011	Radiology	Ineligible population
Feto-maternal risk factor associated to the moderately and extremely obese pregnant woman in comparison to the normal weighted pregnant cases (primigravida and multigravida cases): a comparative cohort research	Alamgir S et al	2018	Indo Am. J. Pharm. Sci.	Ineligible population
Early onset conjugated hyperbilirubinemia in newborn infants	Tiker F et al	2006	Indian Journal of Pediatrics	Ineligible setting
Neonatal mortality at Olabisi Onabanjo University Teaching Hospital, Sagamu	Ogunlesi TA et al	2008	Nigerian Journal of Paediatrics	Ineligible setting
Epidemiology and antimicrobial susceptibility of invasive Escherichia coli infection in neonates from 2012 to 2019 in Xiamen, China	Lai J et al	2021	BMC Infectious Diseases	Ineligible setting
Determinants de la mortality neonatale, dans une population tunisienne	Nouaili Hamida EB et al	2020	La tenisie Medicale	Ineligible setting
Evolution de la mortalitity neoatale au CHU de Blida (Alerie) de 1999-2006	Bezzaoucha A et al	2010	Bulletin de la Societe de Pathologie Exotique	Ineligible setting
Morbidities & outcomes of a neonatal intensive care unit in a complex humanitarian conflict setting, Hajjah Yemen: 2017-2018	Eze P et al	2020	Confl. Health	Ineligible setting
Time to death and its predictors among neonates admitted in the intensive care unit of the University of Gondar Comprehensive Specialized Hospital, Northwest Ethiopia	Gudayu TW et al	2020	Res. Rep. Neonatol.	Ineligible setting
Risk factors for neonatal mortality at St Camille Hospital in Ouagadougou, Burkina Faso	Ouedraogo P et al	2020	Int. J. Pediatr.-Masshad	Ineligible setting
Incidence and predictors of neonatal mortality among neonates admitted in Amhara regional state referral hospitals, Ethiopia: prospective follow up study	Mengistu BA et al	2020	BMC Pediatrics	Ineligible setting
Survival status and predictors of mortality among newborns admitted with neonatal sepsis at public hospitals in Ethiopia	Dessu S et al	2020	International Journal of Pediatrics	Ineligible setting
When do newborns die? Timing and cause-specific neonatal death in neonatal intensive care unit at referral hospital in Gedeo Zone: a prospective cohort study	Eshete A & Abiy S	2020	International Journal of Pediatrics	Ineligible setting
Neonatal morbidity and morality in Calabara, Nigeria: a hospital-based study	Udo JJ et al	2008	Nigerian Journal of Clinical Practice	Ineligible setting
Morbiditiy et mortaliy neonatales au CHU Kara (Togo)	Azoumah K et al	2010	Med Afr Noire	Ineligible setting
[Ten years morbidity and mortality of newborns hospitalized at the Clinic El-Fateh Suka (Ouagadougou, Burkina Faso)]	Nagalo K et al	2013	The Pan African Medical Journal	Ineligible setting
[Neonatal morbidity and mortality in 2002-2006 at the Charles de Gaulle pediatric hospital in Ouagadougou (Burkina Faso)]	Koueta F et al	2007	Sante	Ineligible setting
[National reference unit of neonatology: state of play]	Dicko-Traore F et al	2014	Sante publique	Ineligible setting
Clinico-aetiological profile of neonatal seizures and their outcomes in a tertiary care hospital	Babu MC et al	2018	J. Evol. Med. Dent. Sci.	Ineligible setting
Bacteriological profiles of septicaemia in neonates at tertiary care hospital, Gujarat, India	Assudani HJ et al	2015	J. Res. Med. Dent. Sci.	Ineligible setting
Characteristics of neonatal Sepsis at a tertiary care hospital in Saudi Arabia	Al-Matary A et al	2019	J. Infect. Public Health	Ineligible setting
Trends in cause-specific mortality at a Canadian outborn NICU	Simpson CDA et al	2010	Pediatrics	Ineligible setting
Hypoglycaemia in the newborn	Stomnaroska O et al	2017	Prilozi	Ineligible setting
Dynamics and structure of the neonatal mortality rate during 2001-2003 in specialized maternity hospital "Maichin Dom"	Jekova N & Kalaijieva M	2005	Pediatriya	Ineligible setting
Identification of bacterial pathogens and their antimicrobial susceptibility of early onset neonatal sepsis	Bystricka A et al	2014	Journal of Maternal-Fetal and Neonatal Medicine	Ineligible setting
Competing risk survival analysis of time to in-hospital death or discharge in a large urban neonatal unit in Kenya	Aluvaala J et al	2019	Wellcome Open Research	Ineligible setting
Perinatal mortality and severe morbidity in low and high risk term pregnancies in the Netherlands: prospective cohort study	Evers ACC et al	2010	Br. Med. J.	Ineligible setting
Risk factors of mortality in neonatal illness	Gandhi J & Varadarajan P	2016	J. Evol. Med. Dent. Sci	Ineligible setting
Later rather than sooner: the impact of clinical management on timing and modes of death in the last decade	Dupont-Thibodeau A et al	2014	Acta paediatrica	Ineligible setting
Group B Streptococcus and Escherichia coli infections in the intensive care nursery in the era of intrapartum antibiotic prophylaxis	Bauserman MS et al	2013	The Pediatric Infectious Disease Journal	Ineligible setting
Intravenous lines-related sepsis in newborn babies admitted to NICU in a developing country	Bakr AF	2003	Journal of Tropical Pediatrics	Ineligible setting
Neonatal respiratory distress in Misan: causes, risk factors, and outcomes	Aljawadi HFM & Ali EA	2019	Iran. J. Neonatol.	Ineligible setting
Comparison study of causes and neonatal mortality rates of newborns admitted in neonatal intensive care unit of Al-Sadder Teaching Hospital in Al-Amara City, Iraq	Al-Sadi EK	2017	Int. J. Pediatr.-Masshad	Ineligible setting
Epidemiology and outcomes of maternal sepsis in the US	Hensley M & Prescott HC	2019	American Journal of Respiratory and Critical Care Medicine	Ineligible study design
Maternal mortality at a referral hospital in south western Uganda: a 5 year descriptive analysis	Lugobe HM et al	2021	American Journal of Obstetrics and Gynecology	Ineligible study design
The timing of eclampsia in the postpartum period using the nationwide readmission database	Yoselevsky E et al	2020	American Journal of Obstetrics and Gynecology	Ineligible study design
The relationship between severe maternal morbidity and a risk of postpartum readmission among Korean women: a nationwide population-based cohort study	Nam JY & Park EC	2020	BMC Pregnancy Childbirth	Ineligible study design
Obstetric critical care in Victoria, Australia	Duke G et al	2018	Anaesthesia and Intensive Care	Ineligible study design
The WOMAN Trial: clinical and contextual factors surrounding the deaths of 483 women following post-partum hemorrhage in developing countries COMMENT	Picetti R et al	2020	Obstet. Gynecol. Surv.	Ineligible study design
Obstetric critical care admissions in Australia and New Zealand	Maiden M et al	2018	Anaesthesia and Intensive Care	Ineligible study design
Timing of DEATH AND RATES of IVH, RDS, and NEC among infants with neonatal sepsis	Birch MN et al	2019	Obstetrics and Gynecology	Ineligible study design
Caracteristicas epidemiogicas de la mortalidad neonatatal en el Peru, 2011-2012	Avila J et al	2015	Rev Peru Med Exp Salud Puclica	Ineligible study design
Postpartum fever: study of cases in a tertiary hospital	Mejia Jimenez I et al	2016	Journal of Maternal-Fetal and Neonatal Medicine	Ineligible study design
Early complications and management of newborns during the first month of life	Gascoin G	2015	Journal de Gynecologie Obstetrique et Biologie de la Reproduction	Ineligible study design
Burden, differentials, and causes of child deaths in India	Lahariya C & Paul, VK	2010	Indian Journal of Pediatrics	Ineligible study design
Audit on intrapartum and postpartum sepsis	Tan MY et al	2014	BJOG	Ineligible study design
Incidence and risk factors of pregnancy-associated venous thromboembolism in singhealth, a major healthcare cluster in Singapore	Jaya-Bodestyne SL et al	2017	Research and Practice in Thrombosis and Haemostasis	Ineligible study design
Maternal mortality in a rural referral hospital in the Niger Delta, Nigeria	Igberase GO & Ebeigbe PN	2007	J. Obstet. Gynaecol.	Ineligible study design
Timing of elective repeat cesarean delivery at term and maternal outcomes	Tita A	2009	American Journal of Obstetrics and Gynecology	Ineligible study design
Overview of eclampsia at paropakar maternity and women's hospital, Kathmandu, Nepal	Shakya B & Vaidya A	2012	International Journal of Gynecology and Obstetrics	Ineligible study design
Maternal near-miss and quality of care in a rural Rwandan hospital	Richard K et al	2016	BJOG	Ineligible study design
Initiation of breastfeeding and mortality risk for newborn in rural Bangladesh	Rahman MM et al	2017	Annals of Nutrition and Metabolism	Ineligible study design
How fast did newborns die in Nigeria from 2009-2013: a time-to-death analysis using Verbal /Social Autopsy data	Koffi AK et al	2019	Journal of Global Health	Ineligible study design
Rapid deterioration after the first symptom in maternal death	Katsuragi S et al	2014	American Journal of Obstetrics and Gynecology	Ineligible study design
Trends in maternal mortality in a Gambian tertiary health centre	Idoko P et al	2015	International Journal of Gynecology and Obstetrics	Ineligible study design
Risk for postpartum venous thromboembolism readmissions	Wen T et al	2018	American Journal of Obstetrics and Gynecology	Ineligible study design
Neonatal morbidity associated with duration of labor induction	Teal EN et al	2018	Obstetrics and Gynecology	Ineligible study design
Early-onset neonatal infection in Lithuania	Tameliene R et al	2015	J. Pediatr. Neonatal Individ. Med.	Ineligible study design
Risk and benefits of a natural cesarean section: a retrospective cohort study	Posthuma S et al	2015	American Journal of Obstetrics and Gynecology	Ineligible study design
Perinatal asphyxia in term infants and presence of changes in the serial cranial ultrasound: 1, 3 and 28 days old	Orozco Vargas NS et al	2011	Journal of Perinatal Medicine	Ineligible study design
The impact of postpartum haemorrhage (PPH) on maternal morbidity	Mackeen A & Khong SY	2013	Journal of Health and Translational Medicine	Ineligible study design
Epidemiological trends of neonatal sepsis in a county referral hospital in central Kenya	Le Geyt J & Hauck S	2016	Archives of Disease in Childhood	Ineligible study design
Neonatal and maternal outcomes with prolonged second stage of labor	Laughon SK et al	2013	American Journal of Obstetrics and Gynecology	Ineligible study design
Obstetric admissions to critical care: a retrospective audit	Lane S et al	2019	Journal of the Intensive Care Society	Ineligible study design
Neonatal jaundice and its main risk factors - a cross-sectional study	Reis E Melo A et al	2017	Cogent Medicine	Ineligible study design
An analysis of the obstetric admissions to the intensive care unit [ICU] in a large teaching hospital in the UK	Saiq Z et al	2012	International Journal of Gynecology and Obstetrics	Ineligible study design
High maternal mortality in Jigawa State, Northern Nigeria estimated using the sisterhood method	Sharma V et al	2017	BMC Pregnancy and Childbirth	Ineligible study design
Differences in infant and child mortality in 7 counties in North-rhein-Westfalia	Shmuilovich N et al	2011	Rechtsmedizin	Ineligible study design
Autopsy review of neonatal deaths by disseminated herpesvirus infection	Sloan EA et al	2016	Laboratory Investigation	Ineligible study design
A study of a clinical profile of secondary postpartum haemorrhage in Central Women Hospital (Yangon)	Soe S et al	2012	BJOG	Ineligible study design
Maternal mortality factors: a cross sectional study in 8 leading tertiary care hospitals of Lahore, Pakistan	Zareen S & Mursalin SM	2015	International Journal of Gynecology and Obstetrics	Ineligible study design
Maternal deaths due to amniotic fluid embolism. Results from the French confidential enquiry into maternal deaths, 2010-2012	Morau E et al	2018	Anesthesie et Reanimation	Ineligible study design
Timing of delivery and pregnancy outcomes among laboring nulliparous women	Tita A	2010	Reproductive Sciences	Ineligible study design
Maternal mortality in Ethiopia: Most recent national MDSR data	Usmael A et al	2017	BJOG	Ineligible study design
Comparison of epidemiology and clinical characteristics of enterovirus and parechovirus central nervous system infections in infants during the first three weeks of life: a 6-year single-center retrospective study from 2011-2016	Vaidyanathan V & Selvarangan R	2017	Annals of Neurology	Ineligible study design
Human fetal growth is constrained below optimal for perinatal survival	Vasak B et al	2015	Ultrasound in Obstetrics & Gynecology	Ineligible study design
Hypertention and pregnancy in Africa: a real challenge for the doctors with a great burden for the mothers and the newborns in Africa	Toure IA	2018	Journal of Hypertension	Ineligible study design
Saving mother and newborns in Morropon Chulucanas, health region of Piura, Peru	Trelles J et al	2009	International Journal of Gynecology and Obstetrics	Ineligible study design
Neonatal jaundice surveillance - are we winning?	Yasmeen T et al	2019	Archives of Disease in Childhood	Ineligible study design
Clinical characteristics and outcomes of infants with group b streptococcus (GBS) infection in New South Wales (NSW)	Yeo KT et al	2015	Journal of Paediatrics and Child Health	Ineligible study design
Risk factors for venous thromboembolism during pregnancy and the puerperal period. A national cohort study including 900,000 pregnancies in Denmark 1995.2009	Virkus R et al	2012	Acta Obstetricia et Gynecologica Scandinavica	Ineligible study design
Etiologic and clinical features of bacterial meningitis in infants	Vixüan CA et al	2016	BMC Infectious Diseases	Ineligible study design
Initial death notification results from the child health and mortality prevention surveillance (champs) Sierra Leone pilot phase, October 2017 to february 2018	Worrell MC et al	2018	American Journal of Tropical Medicine and Hygiene	Ineligible study design
Peripartum hemorrhage: risk for readmission and costs	Wen T et al	2018	Reproductive Sciences	Ineligible study design
The use of verbal autopsy to determine leading causes of neonatal death in rural Tibet	Westmoreland K et al	2011	Journal of Investigative Medicine	Ineligible study design
Late maternal deaths: a neglected responsibility	Sliwa K & Anthony J	2016	Lancet	Ineligible study design
Maternal near miss in a tertiary care hospital	Sheriar Z & Patil S	2018	International Journal of Gynecology and Obstetrics	Ineligible study design
Perinatal mortality in Suba, Bogota, Colombia. For the year 2008	Restrepo C et al	2011	Journal of Perinatal Medicine	Ineligible study design
Timing of maternal death: levels, trends, and ecological correlates using sibling data from 34 sub-Saharan African countries	Merdad L & Ali, MM	2018	PLoS One	Ineligible study design
The effect of timing of removal of wound dressing on surgical site infection rate after cesarean delivery	Nesrallah M et al	2017	Obstetrics and Gynecology	Ineligible study design
Eclampsia: Incidence, effectiveness of magnesium sulphate and perinatal outcomes at Mpilo Central Hospital, Bulawayo, Zimbabwe	Ngwenya S	2017	BJOG	Ineligible study design
Neonatal cause-of-death estimates for the early and late neonatal periods for 194 countries: 2000-2013	Oza S et al	2015	Bulletin of the World Health Organization	Ineligible study design
The burden of maternal critical care in 365 days at the university of portharcourt teaching hospital Nigeria	Otokwala J	2019	Journal of the Intensive Care Society	Ineligible study design
Maternal and perinatal post-cesarean morbidity and mortality in Benin in 2013	Mongbo V et al	2015	Tropical Medicine and International Health	Ineligible study design
Implementation and outcomes of a national maternal mortality monitoring system in Morocco 2008-2009	Rachid B et al	2011	Tropical Medicine and International Health	Ineligible study design
Maternal and perinatal outcomes in patients with acute pulmonary edema hospitalized in an intensive care unit	Pordeus ACB et al	2016	Obstetrics and Gynecology	Ineligible study design
Evaluation of intensive care mangement on maternal and fetal outcome of severe preeclampsia and eclampsia (El-Minia maternity hospital experience)	Noreldin N et al	2015	International Journal of Gynecology and Obstetrics	Ineligible study design
Eclampsia in a third level Tunisian hospital: from January 2004 to December 2016	Mouna K et al	2018	Annals of Intensive Care	Ineligible study design
Impact of hypertensive disorders of pregnancy on adverse outcomes: a 10-year retrospective double cohort study in Shanghai, China	Miaomiao Z & Li J	2016	Journal of the American College of Cardiology	Ineligible study design
Maternal mortality in an academic hospital in Sao Paulo, Brazil: 10 years experience	Lopes C et al	2009	International Journal of Gynecology and Obstetrics	Ineligible study design
Estimation of daily risk of neonatal death, including the day of birth, in 186 countries in 2013: a vital-registration and modelling-based study	Oza S et al	2014	The Lancet. Global health	Ineligible study design
Obstetric intensive care admissions in a London district general hospital between 2005-2011	Ma L et al	2014	Journal of the Intensive Care Society	Ineligible study design
Description of factors cause indirect death maternal in the district Lebak Banten Province in 2012	Mariana A & Saefuddin H	2017	Journal of Obstetrics and Gynaecology Research	Ineligible study design
Early and late puerperal complications associated with the mode of delivery in a cohort in Brazil	Mascarello KC et al	2018	Brazilian Journal of Epidemiology	Ineligible study design
A review of postnatal readmissions to a busy obstetrics unit	McClean S et al	2017	BJOG	Ineligible study design
Global, regional, and national causes of child mortality: an updated systematic analysis for 2010 with time trends since 2000	Liu L et al	2012	Lancet	Ineligible study design
Maternal safety in South East Asia	Morris J	2012	Journal of Paediatrics and Child Health	Ineligible study design
Causes of mortality in a Sierra Leonean district hospital neonatal unit	Kirolos S & Sesay J	2018	Archives of Disease in Childhood	Ineligible study design
Preeclampsia and the risk of renal disease	Kristensen J et al	2018	Nephrology Dialysis Transplantation	Ineligible study design
Caesarean sections in a national referral hospital in Ethiopia: trends, predictors and outcomes	Kuzma T	2018	International Journal of Gynecology and Obstetrics	Ineligible study design
Implementing statewide severe maternal morbidity review: the Illinois experience.	Koch AR et al	2018	Journal of Public Health Management and Practice	Ineligible study design
Maternal and neonatal outcomes of american indian and alaskan native women living on vs off-reservations in washington state, 2003-2012	Lai J et al	2015	American Journal of Obstetrics and Gynecology	Ineligible study design
Risk factors for neonatal sepsis	Lekic E et al	2017	Journal of Perinatal Medicine	Ineligible study design
A view from the UK: the UK and Ireland confidential enquiry into maternal deaths and morbidity	Knight M & Tuffnell D	2018	Clinical obstetrics and Gynecology	Ineligible study design
Trends in postpartum hemorrhage in high resource countries: a review and recommendations from the International Postpartum Hemorrhage Collaborative Group	Knight M et al	2009	BMC Pregnancy and Childbirth	Ineligible study design
Obstetric hemorrhage management and maternal morbidity among non-hispanic black women	Jayaprakash P et al	2018	Obstetrics and Gynecology	Ineligible study design
Perinatal mortality of the last twenty years in a tertiary Greek hospital	Goudeli C et al	2014	Journal of Maternal-Fetal and Neonatal Medicine	Ineligible study design
Evaluation of community maternal death surveillance and response in saving mothers, giving lives districts-Uganda, 2012-2013	Petersen E et al	2015	International Journal of Gynecology and Obstetrics	Ineligible study design
Medical complications associated with sepsis in obstetric patients	Wood A et al	2016	American Journal of Obstetrics and Gynecology	Ineligible study design
Incidence and clinical presentation of invasive neonatal group B streptococcal infections in Germany	Fluegge K et al	2006	Pediatrics	Ineligible study design
Survival analysis in an obstetric intensive care unit, according diagnosis at admission	Lopes AP et al	2011	Journal of Perinatal Medicine	Ineligible study design
Acute admission for neonatal jaundice screens-time for a rethink?	Mirza M et al	2017	Archives of Disease in Childhood	Ineligible study design
Bangladesh's matlab safe motherhood programme-does it reduce stillbirths, early neonatal deaths and late neonatal deaths?	Roy S & Ronsmans C	2012	International Journal of Gynecology and Obstetrics	Ineligible study design
Severe maternal morbidity and mortality due to postpartum infection: a cross-sectional analysis from Rwanda	Rulisa S et al	2015	International Journal of Gynecology and Obstetrics	Ineligible study design
Assessing maternal death causes in developing countries; comparing internal death audit to external confidential enquiries into maternal deaths at a referral hospital in Tanzania	Sorensen BL	2012	International Journal of Gynecology and Obstetrics	Ineligible study design
Contemporary trends in adverse neonatal outcomes	Stahl C-LV et al	2019	American Journal of Obstetrics and Gynecology	Ineligible study design
Time from diagnosis to hospitalization for preeclampsia (PE): patient characteristics and outcomes in a multicenter nulliparous cohort	Tita A	2016	American Journal of Obstetrics and Gynecology	Ineligible study design
Risk factors for readmission due to infection after cesarean delivery	Kawakita T & Tefera E	2018	Obstetrics and Gynecology	Ineligible study design
Maternal mortality in central India: Where are we lacking?	Kedar K	2018	International Journal of Gynecology and Obstetrics	Ineligible study design
Global, regional, and national levels of maternal mortality, 1990-2015: a systematic analysis for the Global Burden of Disease Study 2015	Kassebaum NJ et al	2016	Lancet	Ineligible study design
Maternal 'near miss' at Hospital National Guido Valadares (HNGV) - an audit of maternal mortality and morbidity at a tertiary hospital in Timor-Leste	Jayaratnam S et al	2017	Journal of Obstetrics and Gynaecology Research	Ineligible study design
Prognosis score and maternal outcome of eclampsia in a teaching hospital	Jesmin Z	2015	International Journal of Gynecology and Obstetrics	Ineligible study design
National, regional, and global levels and trends in neonatal mortality between 1990 and 2017, with scenario-based projections to 2030: a systematic analysis	Hug L et al	2019	The Lancet. Global health	Ineligible study design
Stillbirths and neonatal deaths among women with postpartum haemorrhage: an analysis of rates and risks in the WOMAN trial	Hough A et al	2019	BJOG	Ineligible study design
Intentional search for maternal deaths in Mexico: socio-demographic disparities between indirect and direct obstetric deaths	Hogan MC et al	2015	International Journal of Gynecology and Obstetrics	Ineligible study design
Publicly funded homebirth in Australia: a review of maternal and neonatal outcomes over 6 years	Catling-Paull C et al	2013	The Medical Journal of Australia	Ineligible study design
Predictors of maternal sepsis: a population-based cohort study	Cassidy AG et al	2019	American Journal of Obstetrics and Gynecology	Ineligible study design
Delivery approach from 37 weeks of gestation in preeclampsia without gravity signals: Maternal and N	Ferreira L et al	2018	International Journal of Gynecology and Obstetrics	Ineligible study design
Escaped maternal deaths in a remote district of Sri Lanka	Fernando TRN	2012	BJOG	Ineligible study design
Glucose-6-phosphate dehydrogenase deficiency in neonatal hyperbilirubinemia: Hacettepe University experience	Celik HT et al	2010	Early Human Development	Ineligible study design
Time trends and causes of maternal mortality in Ceara State, Brazil from 2010 to 2014: necropsy study and lessons from pathology	CarneiroMelo J et al	2017	Virchows Archiv	Ineligible study design
Parto-analgesia and post-partum blood loss	Driul L et al	2011	Zeitschrift fur Geburtshilfe und Neonatologie	Ineligible study design
Neonatal pneumonia in developing countries	Duke T	2005	Archives of Disease in Childhood	Ineligible study design
Induction of labor for gestational hypertension at term: a look at outcomes	Durst J et al	2015	American Journal of Obstetrics and Gynecology	Ineligible study design
Timing of uterine tamponade and associated morbidity in patients with stage 3 postpartum hemorrhage	Ernst A et al	2018	Obstetrics and Gynecology	Ineligible study design
Postpartum readmission and severe maternal morbidity in California	Girsen AI et al	2017	American Journal of Obstetrics and Gynecology	Ineligible study design
Impact of implementing an obstetric hemorrhage consensus bundle in a large health system	Hacker FM et al	2019	American Journal of Obstetrics and Gynecology	Ineligible study design
Maternal and neonatal complications of severe preeclampsia: preliminary prospective study	Garcia Garcia C et al	2012	European Journal of Anaesthesiology	Ineligible study design
Maternal death audit reviews at three hospitals in Uganda	Frank K et al	2012	International Journal of Gynecology and Obstetrics	Ineligible study design
Fetal and neonatal deaths-evaluation of prevention, quality and shortcomings in newborn and maternal health care	Fonseca C et al	2016	European Journal of Pediatrics	Ineligible study design
Incidence and causes of maternal mortality in Montenegro	Colakovic-Popovic V et al	2011	Journal of Perinatal Medicine	Ineligible study design
Unprecedented rates of PPH: a prospective observational cohort study of blood loss in childbirth (the stop study)	Briley A et al	2012	Archives of Disease in Childhood: Fetal and Neonatal Edition	Ineligible study design
Serious peripartum complications needing admission in obstetrical ICU: retrospective study about 127 cases	Brahim A et al	2016	Annals of Intensive Care	Ineligible study design
Does an increasing elective caesarean section rate protect against hypoxic ischaemic encephalopathy?	Battersby AH & Morris SA	2015	Journal of Paediatrics and Child Health	Ineligible study design
Prolonged jaundice in infants	Cartledge P & McClean P	2009	Community Practitioner	Ineligible study design
Countdown to 2015 for maternal, newborn, and child survival: the 2008 report on tracking coverage of interventions	Countdown Coverage Writing Group et al.	2008	Lancet	Ineligible study design
Impact of maternal age and parity in management and outcome of major obstetric haemorrhage	Oconnor H et al	2012	American Journal of Obstetrics and Gynecology	Ineligible study design
Maternal mortality in teritiary care centre-3-year study	Sangabathula H et al	2014	BJOG	Ineligible study design
Cause-specific mortality at INDEPTH Health and Demographic Surveillance System Sites in Africa and Asia: concluding synthesis	Sankoh O & Byass P	2014	Global Health Action	Ineligible study design
Obstetric admissions to the intensive care unit: the role of pre-eclampsia	Sass N et al	2010	Pregnancy Hypertension	Ineligible study design
Maternal death surveillance and response: opportunities to reduce maternal mortality in Uganda	Serbanescu F et al	2018	International Journal of Gynecology and Obstetrics	Ineligible study design
An audit of primary postpartum haemorrhage at tertiary care hospital	Shafi F et al	2013	BJOG	Ineligible study design
Maternal admissions to critical care - a 10 year review	Anderson FJ & Joss JA	2011	International Journal of Obstetric Anesthesia	Ineligible study design
National audit of maternal morbidity in Scotland	Cameron A	2013	Journal of Perinatal Medicine	Ineligible study design
What is the most appropriate timing for prophylactic antibiotics during caesarean section? A literature review	Baker H et al	2018	BJOG	Ineligible study design
Incidence, characteristics and outcomes of pregnancy-related critical illness over time in Canada	Aoyama K et al	2012	Intensive Care Medicine	Ineligible study design
Induction for nonmedical indications compared with expectant management	Bailit J	2014	American Journal of Obstetrics and Gynecology	Ineligible study design
Biochemical changes in eclampsia patients in a tertiary level hospital of Bangladesh	Banu L	2009	International Journal of Gynecology and Obstetrics	Ineligible study design
A 5 years review of maternal mortality in FMH	Ambreen A et al	2015	BJOG	Ineligible study design
Perinatal mortality rate of Kutahya province and the analysis of etiological factors, Turkey	Aksaz Z et al	2010	Journal of Maternal-Fetal and Neonatal Medicine	Ineligible study design
Reduction of maternal and fetal mortality and morbidity in hospitals in Nigeria by quality management in obstetrics - results of a pilot project	Adams S.et al	2012	Journal of Maternal-Fetal and Neonatal Medicine	Ineligible study design
Maternal sepsis and associated mortality: a population-based cohort of 13 million births	Akim V et al	2019	American Journal of Obstetrics and Gynecology	Ineligible study design
Rates of postpartum hemorrhage and related interventions: United States, 2000-2012	Ahmadzia HK et al	2016	American Journal of Obstetrics and Gynecology	Ineligible study design
Hypoxic ischaemic encephalopathy in a tertiary obstetric unit: a review of the obstetric, anaesthetic and neonatal factors	Agarwal DK et al	2015	International Journal of Obstetric Anesthesia	Ineligible study design
Clinical and epidemiological aspects of stroke associated with pregnancy and the puerperium	Abassova G et al	2017	Journal of the Neurological Sciences	Ineligible study design
Epidemiology of neonatal jaundice at the University Hospital of the West Indies	Henny-Harry C & Trotman H	2012	The West Indian Medical Journal	Ineligible time frame
Changes in incidence and etiology of early-onset neonatal infections 1997-2017 - a retrospective cohort study in western Sweden	Johansson Gudjonsdottir M et al	2019	BMC pediatrics	Ineligible time frame
Verbal autopsy to ascertain causes of neonatal deaths in a community setting: a study from Morang, Nepal	Khana S et al	2011	Journal of the Nepal Medical Association	Ineligible time frame
Determinants of neonatal mortality in Pakistan: secondary analysis of Pakistan Demographic and Health Survey 2006-07	Nisar YB et al	2014	BMC Public Health	Ineligible time frame
Determinants of neonatal mortality in Indonesia	Titaley CR et al	2008	BMC Public Health	Ineligible time frame
Association of Unexpected Newborn Deaths With Changes in Obstetric and Neonatal Process of Care.	Han D et al	2020	JAMA Network Open	Ineligible time frame
Screening for early-onset invasive group B Streptococcal disease in neonates in an Irish hospital (2001-2014): a retrospective audit	Nielsen M et al	2017	Infectious diseases	Ineligible time frame
Challenge of reducing perinatal mortality in rural Congo: findings of a prospective, population-based study	Matendo RM et al	2011	Journal of Health, Population and Nutrition	Ineligible time frame
Causes of community stillbirths and early neonatal deaths in low-income countries using	Engmann C et al	2012	Journal of Perinatology	Ineligible time frame
Stillbirths and early neonatal mortality in rural Northern Ghana	Engmann C et al	2012	Tropical Medicine & International Health	Ineligible time frame
Surveillance of surgical site infection after cesarean section and time of notification	Lima J et al	2016	American Journal of Infection Control	Ineligible time frame
Maternal and obstetric factors associated with delayed postpartum eclampsia: a national study population	Kayem G et al	2011	Acta obstetricia et gynecologica Scandinavica	Ineligible time frame
Clinical course, associated factors, and blood pressure profile of delayed-onset postpartum preeclampsia	Redman EK et al	2019	Obstetrics and Gynecology	Ineligible time frame
Early post partum discharge: is it possible?	Sadeh-Mestechkin D et al	2007	Archives of Gynecology and Obstetrics	Ineligible time frame
Les morts maternelles en France: mieux comprendre pour mieux prevenir	INSERM Sante Publique France	2017	N/A	Ineligible time frame
Causes and timing of maternal death in Mizan - Tepi university teaching and Bonga general hospital from 2011-2015: a case control study and using propensity score matching analysis	Dadi TL et al	2017	Open Public Health Journal	Ineligible time frame
Saving lives, improving mother's care report	Outcome, Infant Clinical; Programme, Review	2015	Midwifery	Ineligible time frame
The WOMAN trial: clinical and contextual factors surrounding the deaths of 483 women following post-partum haemorrhage in developing countries	Picetti R et al	2020	BMC Pregnancy and Childbirth	Ineligible time frame
Have maternal mortalities been decreased since last decade with improving maternity care?	Işik H et al	2016	Journal of Clinical and Analytical Medicine	Ineligible time frame
Epidemiology of pregnancy-associated pulmonary embolism in South Asian multi-ethnic country: mortality trends over the last four decades	Tan TC et al	2021	The Journal of Obstetrics and Gynaecology Research	Ineligible time frame
Review of causes of maternal deaths in Botswana in 2010	Ray S et al	2013	South African Medical Journal	Ineligible time frame
Effect of training traditional birth attendants on neonatal mortality (Lufwanyama Neonatal Survival Project): randomised controlled study	Gill CJ et al	2011	BMJ	Ineligible time frame
Etiologies and contributing factors of perinatal mortality: a report from southeast of Iran	Hadavi M et al	2011	Taiwan. J. Obstet. Gynecol.	Ineligible time frame
[Application of the international classification of diseases for perinatal mortality (ICD-PM) to vital statistics records for the purpose of classifying perinatal deaths in antioquia, Colombia]	Salazar-Barrientos M et al	2019	Revista colombiana de obstetricia y ginecologia	Ineligible time frame
Risk factors and isolated microorganisms in patients with neonatal sepsis	Morales LP et al	2021	Medisur-Rev. Cienc. Med. Cienfuegos	Ineligible time frame
Neonatal mortality within 24 hours of birth in six low- and lower-middle-income countries	Baqui AH et al	2016	Bulletin of the World Health Organization	Ineligible time frame
Maternal and perinatal outcomes by planned place of birth in Australia 2000-2012: a linked population data study	Homer CSE et al	2019	BMJ Open	Ineligible time frame
Trend in infant mortality rate caused by sepsis in Brazil from 2009 to 2018	Rodrigues LDS et al	2021	Revista do Instituto de Medicina Tropical de Sao Paulo	Ineligible time frame
The impact of implementing the 2016 WHO Recommendations on Antenatal Care for a Positive Pregnancy Experience on perinatal deaths: an interrupted time-series analysis in Mpumalanga province, South Africa	Lavin T et al	2020	BMJ Global Health	Ineligible time frame
The application of WHO ICD-PM: feasibility for the classification of timing and causes of perinatal deaths in a busy birth centre in a low-income country	Housseine N et al	2021	PLoS One	Ineligible time frame
Trends, patterns and cause-specific neonatal mortality in Tanzania: a hospital-based retrospective survey	Mangu CD et al	2020	International Health	Ineligible time frame
Neonatal mortality in the urban and rural China between 1996 and 2013: a retrospective study	Lu R et al	2016	Pediatric Research	Ineligible time frame
Neonatal mortality and causes of death in Kersa Health and Demographic Surveillance System (Kersa HDSS), Ethiopia, 2008-2013	Assefa N et al	2016	Maternal Health, Neonatology and Perinatology	Ineligible time frame
Trend and causes of neonatal mortality in the Kassena-Nankana district of northern Ghana, 1995-2002	Baiden F et al	2006	Tropical Medicine and International Health	Ineligible time frame
[Perinatal mortality at Hospital de Ginecoobstetricia No. 23 of Monterrey, Nuevo Leon, 2002-2006 period]	Gutierrez Saucedo ME et al	2008	Ginecologia y obstetricia de Mexico	Ineligible time frame
A case series study of perinatal deaths at one referral center in rural post-conflict Liberia	Lori JR et al	2014	Maternal and Child Health Journal	Ineligible time frame
Neonatal mortality in Argentina. Situation analysis from 2005 to 2014	Finkelstein JZ et al	2017	Archivos argentinos de pediatria	Ineligible time frame
Tracking progress on the health status and service delivery outcomes for neonates and children in the metro west geographic service area of the cape metropole, 2010 - 2015	Hendricks MK et al	2019	South African Journal of Child Health	Ineligible time frame
Prospective community-based cluster census and case-control study of stillbirths and neonatal deaths in the West Bank and Gaza Strip	Kalter HD et al	2008	Paediatric and Perinatal Epidemiology	Ineligible time frame
Maldives Health Statistics 2015-16	Ministry of Health	2019	N/A	Ineligible time frame
The study of etiological and demographic characteristics of neonatal mortality and morbidity - a consecutive case series study from Pakistan	Manzar N et al	2012	BMC Pediatrics	Ineligible time frame
Differences in mortality between late-preterm and term singleton infants in the United States, 1995-2002	Tomashek KM et al	2007	The Journal of Pediatrics	Ineligible time frame
Neonatal mortality in Sri Lanka: timing, causes and distribution	Rajindrajith S et al	2009	The Journal of Maternal-Fetal & Neonatal Medicine	Ineligible time frame
Neonatal mortality in rural Bangladesh: an exploratory study	Chowdhury ME at al	2005	Journal of Health, Population and Nutrition	Ineligible time frame
Why do neonates die in rural Gadchiroli, India? (Part I): primary causes of death assigned by neonatologist based on prospectively observed records	Bang AT et al	2005	Journal of Perinatology	Ineligible time frame
The Egypt national perinatal/neonatal mortality study 2000	Campbell O et al	2004	Journal of Perinatology	Ineligible time frame
[Verbal autopsy to measure maternal mortality in rural Senegal].	Ba MG et al	2003	Journal de gynecologie, obstetrique et biologie de la reproduction	Ineligible time frame
Postpartum invasive group A streptococcal disease in the modern era.	Aronoff DM & Mulla ZD	2008	Infectious Diseases in Obstetrics and Gynecology	Ineligible time frame
Postpartum stroke: a twenty-year experience	Witlin AG et al	2000	American Journal of Obstetrics and Gynecology	Ineligible time frame
Jaundice noted in the first 24 hours after birth in a managed care organization	Newman TB et al	2002	Archives of Pediatrics & Adolescent Medicine	Ineligible time frame
Expectant management of early onset, severe pre-eclampsia: maternal outcome	Hall DR et al	2000	BJOG	Ineligible time frame
Risk of death following pregnancy in rural Nepal	Pradhan EK et al	2002	Bulletin of the World Health Organization	Ineligible time frame
Maternal and fetal risks associated with prolonged latent phase of labour.	Maghoma J & Buchmann EJ	2002	Journal of Obstetrics and Gynaecology	Ineligible time frame
Maternal mortality in a tertiary care teaching hospital	Akbar N et al	2002	Journal of the College of Physicians and Surgeons Pakistan	Ineligible time frame
One year survey of maternal mortality associated with eclampsia in Dhaka Medical College Hospital	Hussain F et al	2000	Journal of Obstetrics and Gynaecology	Ineligible time frame
Can improvements in breast-feeding practices reduce neonatal mortality in developing countries?	Huffman SL et al	2001	Midwifery	Ineligible time frame
Maternal mortality: only 42 days?	Hoj L et al	2003	BJOG	Ineligible time frame
Association between duration of neonatal hospital stay and morbidity in the first month of life	Hatzidaki EG et al	2001	Clinical and Experimental Obstetrics & Gynecology	Ineligible time frame
No increase in rates of early-onset neonatal sepsis by antibiotic-resistant group B Streptococcus in the era of intrapartum antibiotic prophylaxis	Chen KT et al	2005	American Journal of Obstetrics and Gynecology	Ineligible time frame
An epidemiological survey on neonatal jaundice in China	Ding G et al	2001	Chinese Medical Journal	Ineligible time frame
Factors affecting perinatal mortality in India (perinatal audit)	Shah D et al	2000	Prenat. Neonatal Med.	Ineligible time frame
Follow-up interviews after eclampsia	Andersgaard AB et al	2009	Gynecologic and obstetric investigation	Ineligible time frame
Neonatal sepsis: an etiological study	Anwer SK et al	2000	The Journal of the Pakistan Medical Association	Ineligible time frame
Postpartum haemorrhage in nulliparous women: incidence and risk factors in low and high risk women. A Dutch population-based cohort study on standard (> or = 500 ml) and severe (> or = 1000 ml) postpartum haemorrhage	Bais JMJ et al	2004	European journal of obstetrics, gynecology, and reproductive biology	Ineligible time frame
Neonatal mortality of inborns in the neonatal unit of a tertiary centre in Lagos, Nigeria	Ekure E et al	2008	Nigerian Quarterly Journal of Hospital Medicine	Reports not retrieved
Risk factors associated with mortality in the neonatal nosocomial infection	Coria Lorenzo J et al	2005	Saludarte	Reports not retrieved
Medical audit for the neonatal unit of Dhaka Medical College Hospital	Afroza S et al	2001	Perinatology	Reports not retrieved
Risk factors for neonatal sepsis	Qureshi D et al	2010	Medical Forum Monthly	Reports not retrieved
[Epidemiological characteristics of neonatal mortality in Peru, 2011-2012]	Ávila et al	2015	Rev Peru Med Exp Salud Publica	Ineligible outcome
Severe maternal sepsis in the UK, 2011-2012: a national case-control study	Acosta et al	2014	PLoS Medicine	Reported on maternal outcomes
Maternal deaths in Australia, 2015-2017	Australian Institute of Health and Welfare	2020	N/A	Reported on maternal outcomes
An analysis of pregnancy-related mortality in the KEMRI/CDC health and demographic surveillance system in western Kenya	Desai et al.	2013	PLoS One	Reported on maternal outcomes
Severe secondary postpartum hemorrhage: a historical cohort	Dossou et al	2015	Birth	Reported on maternal outcomes
Socio-economic disparities in maternal mortality in China between 1996 and 2006	Feng et al	2010	BJOG	Reported on maternal outcomes
Risk factors and maternal outcome of secondary post partum haemorrhage in rangpur medical college hospital - a one year study	Ferdousy et al	2020	Bangladesh Journal of Obstetrics and Gynecology	Reported on maternal outcomes
Incidence and risk factors of venous thromboembolism during postpartum period: a population-based cohort-study	Galambosi et al	2017	Acta obstetricia et gynecologica Scandinavica	Reported on maternal outcomes
National Maternal Mortality Study, 2005	Hacettepe University Institute of Population Studies	2006	N/A	Reported on maternal outcomes
Pregnancy-related deaths in rural Rajasthan, India: exploring causes, context, and care-seeking through verbal autopsy	Iyengar et al	2009	Journal of Health, Population and Nutrition	Reported on maternal outcomes
Plan national 2008-2012 pour l’accélération de la réduction de la mortalité maternelle et infantile: rapport national de l’enquête confidentielle sur les décès maternels au Maroc	Kingdom of Morocco	2010	N/A	Reported on maternal outcomes
Plan d’action 2012 – 2016 pour accélérer la réduction de la mortalité maternelle et néonatale: enquête confidentielle sur les décès maternels de 2010 au Maroc	Kingdom of Morocco	2013	N/A	Reported on maternal outcomes
Vital signs: pregnancy-related deaths, United States, 2011-2015, and strategies for prevention, 13 States, 2013-2017.	Petersen et al	2019	Morbidity and Mortality Weekly Report	Reported on maternal outcomes
Preeclampsia-eclampsia and the risk of stroke among peripartum in Taiwan	Tang et al	2009	Stroke	Reported on maternal outcomes
Postpartum venous thromboembolism: incidence and risk factors	Tepper et al	2014	Obstetrics and Gynecology	Reported on maternal outcomes
Incidence and characteristics of pregnancy-related death across ten low- and middle-income geographical regions: secondary analysis of a cluster randomised controlled trial	Vousden et al	2020	BJOG	Reported on maternal outcomes
